# One-Component Nanocomposites Made from Diblock Copolymer Grafted Cellulose Nanocrystals

**DOI:** 10.1021/acs.biomac.3c01196

**Published:** 2024-02-21

**Authors:** Chris Rader, Patrick W. Fritz, Timur Ashirov, Ali Coskun, Christoph Weder

**Affiliations:** † Adolphe Merkle Institute, University of Fribourg, Chemin des Verdiers 4, 1700 Fribourg, Switzerland; ‡ Department of Chemistry, University of Fribourg, Chemin de Musee 9, 1700 Fribourg, Switzerland

## Abstract

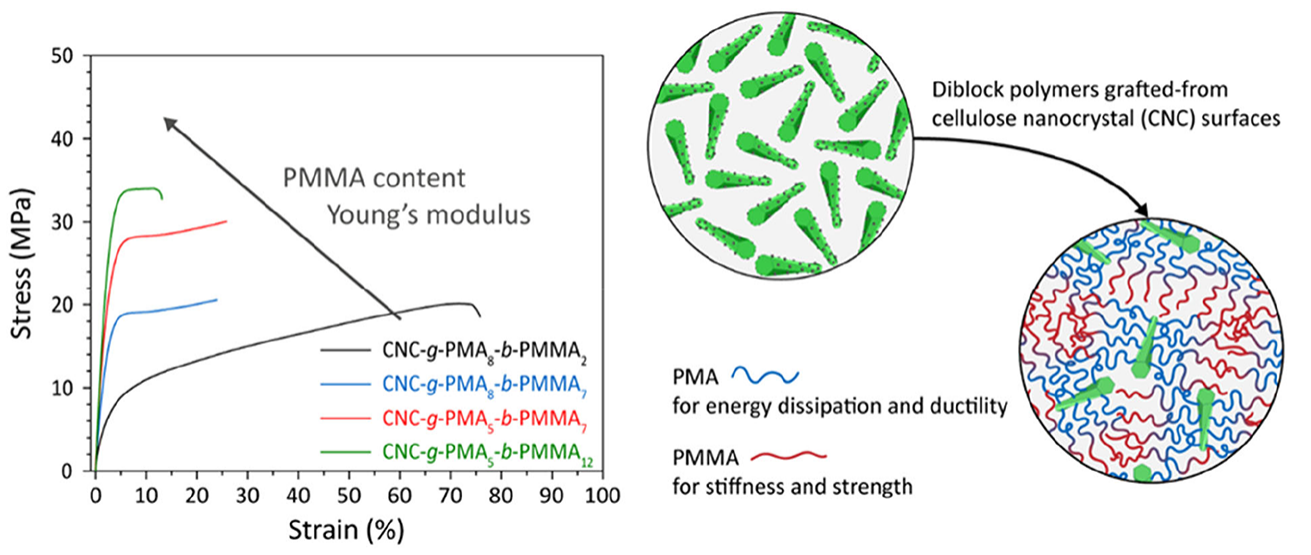

Cellulose nanocrystals (CNCs) are bio-based, rod-like, high-aspect-ratio nanoparticles with high stiffness and strength and are widely used as a reinforcing nanofiller in polymer nanocomposites. However, due to hydrogen-bond formation between the large number of hydroxyl groups on their surface, CNCs are prone to aggregate, especially in nonpolar polymer matrices. One possibility to overcome this problem is to graft polymers from the CNCs’ surfaces and to process the resulting “hairy nanoparticles” (HNPs) into one-component nanocomposites (OCNs) in which the polymer matrix and CNC filler are covalently connected. Here, we report OCNs based on HNPs that were synthesized by grafting gradient diblock copolymers onto CNCs via surface-initiated atom transfer radical polymerization. The inner block (toward the CNCs) is composed of poly(methyl acrylate) (PMA), and the outer block comprises a gradient copolymer rich in poly(methyl methacrylate) (PMMA). The OCNs based on such HNPs microphase separate into a rubbery poly(methyl acrylate) phase that dissipates mechanical energy and imparts toughness, a glassy PMMA phase that provides strength and stiffness, and well-dispersed CNCs that further reinforce the materials. This design afforded OCNs that display a considerably higher stiffness and strength than reference diblock copolymers without the CNCs. At the same time, the extensibility remains high and the toughness is increased up to 5-fold relative to the reference materials.

## Introduction

Cellulose nanocrystals (CNCs) are bio-based, rod-like, high-aspect-ratio nanoparticles, which on account of their high crystallinity and the uniaxial orientation of the macromolecules along the particles’ axis exhibit very high stiffness and strength.^1,2^ The dimensions, mechanical characteristics, and colloidal characteristics of CNCs depend on the biosource and the isolation method.^3−5^ The average diameter and length range from 2 to 30 nm and 500 to 2000 nm, respectively. CNCs have been widely studied as reinforcing nanofillers in polymers.^6−9^ They offer several advantages over, e.g., carbon nanotubes, including sustainable sourcing, low production cost, and low cytotoxicity.^10^ The surface of CNCs features an abundance of hydroxyl groups, which enables their dispersion in water and other polar solvents.^11^ In polymer nanocomposites, the CNCs can form percolating networks, in which interfacial hydrogen bonds promote stress transfer among the particles.^12,13^ These interactions also make CNCs prone to aggregation, and consequently, many nanocomposites comprising such particles have been reported to exhibit mechanical properties that are lower than the values predicted by composite models.^14^ To counterbalance hydrogen bonding, partially sulfonated CNCs, which are obtained by hydrolysis of cellulose pulp with sulfuric acid hydrolysis,^15^ are frequently used. The electrostatic repulsion between anionic surface groups enhances their dispersibility in water and polar solvents such as DMF.^16^ TEMPO-mediated oxidation is another strategy to bestow CNCs with good dispersibility and alternative reactive sites.^17,18^ A prominent approach to improve the CNC dispersibility in polymers, which has been explored in numerous systems with different levels of success, includes the use of polymeric or low-molecular-weight “surfactants”.^11,19−24^

**Figure 1:**
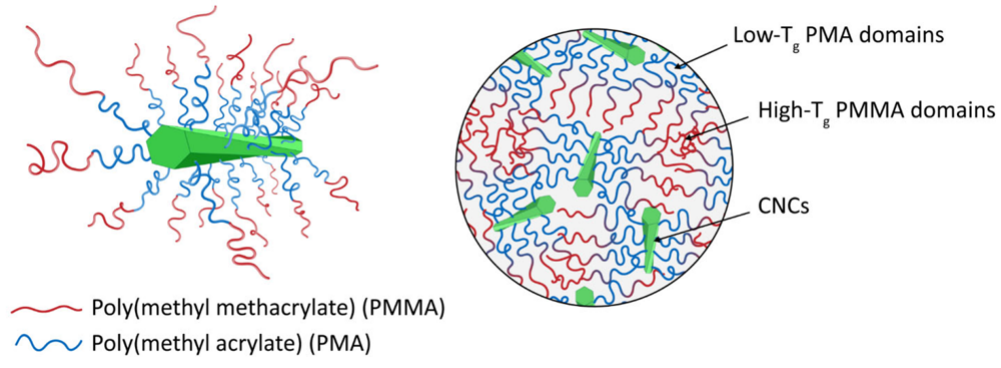
Schematic of hairy nanoparticles (HNPs) made by grafting CNCs with a gradient diblock copolymer (left) and one-component nanocomposites (OCNs) formed by these particles. Microphase separation promotes the formation of a rubbery poly(methyl acrylate) phase that dissipates mechanical energy and imparts toughness, a glassy PMMA phase that provides strength and stiffness, and well-dispersed CNCs that further reinforce the material.

Another possibility to enhance the dispersibility of CNCs in a polymer is to graft their surface with a polymer of the same or different nature.^25−27^ Taking this approach to the limit, it is also possible to omit an auxiliary matrix polymer and assemble the polymer-grafted, “hairy” nanoparticles (HNPs) into materials that are termed one-component nanocomposites (OCNs).^28^ Because the polymer is covalently attached to the surface of nanoparticles, aggregation and macrophase separation effects are eliminated. OCNs based on CNCs isolated from cotton that were decorated with glassy poly(methyl methacrylate) (PMMA) or rubbery poly(hexyl methacrylate (PHMA) have been recently reported.^29^ These nanomaterials were accessed by functionalizing the CNCs with a photoinitiator and surface-initiated free-radical photopolymerizations of methyl methacrylate or hexyl methacrylate. The OCNs based on such HNPs displayed a remarkable improvement in stiffness, toughness, or strength compared to two-component nanocomposites of unmodified CNCs and these polymers, depending on the nature of the grafts. Here we report OCNs made from HNPs that were synthesized by grafting gradient diblock copolymers from the CNCs via surface-initiated atom transfer radical polymerization. The inner block (toward the CNCs) is composed of poly(methyl acrylate) (PMA), and the outer block comprises a gradient copolymer rich in PMMA. This design is rooted in our hypothesis that OCNs based on such HNPs should microphase separate under the formation of a rubbery poly(methyl acrylate) phase that dissipates mechanical energy and imparts toughness, a glassy PMMA phase that provides strength and stiffness, and well-dispersed CNCs that further reinforce the materials (Figure 1). We expected that this architecture would give rise to a property matrix that combines high stiffness, strength, and toughness. While the grafting of stimuli-responsive diblock copolymers from CNCs has been reported,^30,31^ the solid-state properties of OCNs based on this design appear to be unexplored.

To achieve a high grafting density, we targeted a grafting-from approach^32^ based on surface-initiated atom transfer radical polymerization (SI-ATRP) from initiator-modified CNCs.^33^ This general methodology has recently been used to decorate CNCs with a variety of polymers, including poly(styrene),^34^ poly(methyl acrylate),^35^ poly(methyl methacrylate),^36,37^ and poly(*N*-isopropylacrylamide).^38^ Applying the same framework to diblock copolymers is challenging because only highly polar organic solvents can disperse the CNCs.^11,16^ Yet, the solvent must also dissolve both polymers and support the ATRP mechanism, and the polymerization must remain living once the first block has formed. Moreover, the characterization of grafts grown from the surface of CNCs, notably their molecular weight, represents a particular challenge. Conventional solution-based approaches are not applicable, and cleavage of the polymers after the HNPs are made is usually not straightforward and does not allow for *in situ* monitoring.^39^ Many studies have relied on an auxiliary “sacrificial” initiator, which is added to the reaction mixture to grow free polymer as a solution-characterizable proxy for the polymers grown from the CNCs.^39−42^ However, such *in situ* model reactions do not necessarily represent the kinetics of the surface-initiated polymerization reactions well. We addressed these challenges by developing a single electron transfer ATRP (SET-ATRP) gradient method, in which Cu^0^ wire and Cu^II^ are used as a source of the copper catalyst,^35,43^ dimethyl sulfoxide (DMSO) serves as the solvent, and the monomer addition/conversion is controlled to afford relatively pure blocks, even though the reaction becomes uncontrolled. We originally planned to monitor the polymerization by *in situ*^1^H NMR spectroscopic investigation of the monomer consumption against an internal standard^36^ but discovered that the polymers grown from the CNCs could directly be monitored by solution-phase ^1^H NMR spectroscopy, which, as reported by Kim et al. for poly(methacrylate)-grafted silica nanoparticles, is possible due to the excellent dispersibility of the HNPs and highly soluble polymer grafts.^44^

## Experimental Section

### Materials

All chemicals were purchased from Sigma-Aldrich and were used as received unless otherwise stated, except CDCl_3_ which was purchased from Cambridge Isotope Laboratories, Inc. Methyl acrylate (MA) and methyl methacrylate (MMA) were purified by filtration through alumina. Milli-Q water was produced using a Sartorius arium pro VF/UF (Sartorius AG, Göttingen, Germany) water purification system with Sartopore 2 150 filtration columns.

### Isolation of CNCs

Sulfated cellulose nanocrystals were isolated by sulfuric acid hydrolysis of Whatman No. 1 filter paper using a previously reported protocol.^45^ The filter paper was cut into small pieces (30 g) and added to 64 wt % H_2_SO_4_ (400 mL) that had previously been heated to 55 °C. The mixture was then magnetically stirred at 55 °C for 60 min before the reaction was quenched by diluting the reaction mixture with deionized H_2_O (1000 mL) and cooling in an ice bath. The CNCs were separated from the liquid by centrifugation using a Beckman Coulter centrifuge at a speed of 20000*g* for 20 min. During centrifugation, the temperature was maintained at 10 °C. After centrifugation, the supernatant was decanted and replaced with deionized H_2_O. Three centrifugation cycles were carried out; after the last one, the supernatant was colorless. The CNC dispersion was then dialyzed in deionized H_2_O for 5–7 days with water exchanges made every day until the pH of the water was 7. The final suspension was ultrasonicated for 15 min before being lyophilized for 3 days using a Telstar Lyoquest (Terrassa, Spain) at −41.5 °C and at 0.4 mbar of pressure.

### Conductometric Titrations

Conductometric titration was performed following the procedure reported by Beck et al.^46^ with the modification that the CNC suspensions were ultrasonicated for 2 h in a Sonoswiss s3h bath sonicator instead of being horn sonicated and analyzed by using a Mettler Toledo SevenCompact Duo S213 pH/conductivity meter (Greifensee, Switzerland). Conductometric titrations resulted in a concentration of 115 ± 5 mmol kg^–1^ sulfate half-ester groups (R-OSO_3_H).

### Atomic Force Microscopy (AFM)

The average height of CNCs and CNC-Br (CNCs that were surface-modified with BiBB) were obtained from AFM images. Freshly peeled mica was coated with an aqueous solution of poly(l-lysine) (0.1 w/v in H_2_O) that was applied by drop-casting (40 μL). After 5 min, the excess of poly(l-lysine) was washed off with Milli-Q water, and the substrates dried under a flow of nitrogen. Dispersions of CNCs or CNC-Br in water (0.001 wt %) were then spin-coated onto the functionalized mica surface at 2000 rpm and subsequently dried under nitrogen flow. The images were acquired with a JPK Nano Wizard II from JPK BioAFM (Berlin, Germany) in tapping mode with PPP-NCSTR probes at room temperature and using a silicon cantilever.

### Transmission Electron Microscopy (TEM)

TEM micrographs of CNCs and CNC-Br were acquired on a Tecnai Spirit transmission electron microscope (FEI/ThermoFischer, Hillsboro, OR) operating at 120 kV using a 2k Veleta camera. Sample suspensions of 5 μL of 0.03 wt % CNCs in water and 0.03 wt % CNC-Br in THF were spin-coated at 2000 rpm on a previously plasma-treated carbon film 300 mesh copper TEM grids. Deposited samples were left to dry in an oven at 60 °C overnight before being imaged.

### Nuclear Magnetic Resonance (NMR) Spectroscopy

^1^H NMR solvent spectra were measured at 297.2 K on a Bruker Avance DPX 400 spectrometer (Billerica, MA) at a frequency of 400.2 MHz with 32 scans and a 5 s relaxation time. All spectra were referenced to the residual solvent peak of deuteriochloroform (CDCl_3_, 7.26 ppm). Data were analyzed with the MestReNova software.

Solid-state cross-polarization magic angle spinning (CP/MAS) ^13^C NMR spectra were recorded on a Bruker Avance Neo 400 MHz (Billerica, MA) spectrometer using a spinning rate of 10 kHz and a 30.0 s relaxation delay in a 4 mm probe. Further experiments were performed on a Bruker Avance Neo 600 MHz machine (Billerica, MA) using a spinning rate of 60 kHz and a 5 s relaxation time in a 1.6 mm probe. Data were analyzed with the MestReNova software.

### Fourier-Transform Infrared (FT-IR) Spectroscopy

FT-IR spectra were recorded on a PerkinElmer Spectrum 65 (Shelton, CT) spectrometer equipped with an attenuated total reflection (ATR) setup. All spectra were collected in the wavelength range between 600 and 4000 cm^–1^ after 32 continuous scans.

### Elemental Analysis (EA)

EA was performed by the Molecular and Biomolecular Analysis Service MoBiAS at the ETH Zurich, Switzerland, using a Metrohm Eco IC (Herisau, Switzerland), and used to determine the C, H, N, and Br content of CNC and CNC-Br. The combustion products resulting from the sample digestion, i.e., CO_2_ and H_2_O, were quantified by infrared spectroscopy to determine C and H contents, respectively. N was measured as N_2_ by quantification of their thermal conductivity upon burning the sample at 1000 °C.

### Size Exclusion Chromatography (SEC)

SEC experiments were performed on an Agilent 1200 series HPLC system (Santa Clara, CA) equipped with an Agilent PL gel mixed guard column (particle size = 5 μm) and two Agilent PL gel mixed-D columns (ID = 7.5 mm, *L* = 300 mm, particle size = 5 μm). Signals were recorded by an Optilab REX interferometric refractometer and a miniDawn TREOS light scattering detector (Wyatt Technology Corp.). Samples were run using THF as the eluent at 30 °C and a flow rate of 1.0 mL/min. Data analyses were performed on Astra software (Wyatt Technology Corp.), and molecular weights were determined based on narrow molecular weight poly(methyl methacrylate) standards calibration (from 540 to 2210000 g/mol).

### Thermogravimetric Analysis (TGA)

TGA was performed with a Mettler-Toledo TGA/DSC 1 Star^e^ System (Greifensee, Switzerland) in the temperature range from 25 to 600 °C with a heating rate of 10 °C min^–1^. Tests were performed under nitrogen with a flow rate of 40 mL min^–1^. TGA data were analyzed using the STARe Evaluation software.

### Dynamic Mechanical Analyses (DMA)

DMA experiments were performed on a TA Instruments Model Q800 DMA (New Castle, DE) in tensile mode. The temperature ranged from −70 to 150 °C with a heating rate of 3 °C min^–1^, a frequency of 1 Hz, and an amplitude of 0.1% strain. Rectangular films with a length of ca. 10 mm, a width of ca. 2.5 mm, and a thickness of ca. 0.23 mm were cut from compression-molded films. The data reported are averages of three independent measurements, and all errors are standard deviations, reported as the variance in a set of samples compared to the mean of the measurement.

### Tensile Tests

Tensile tests were conducted on a Zwick/Roell Z010 (Ulm, Germany) tensile tester following ASTM D882 standards. Tests were performed at room temperature with a strain rate of 50% min^–1^ and a load cell of 200 N. Rectangular films with a length of ca. 10 mm, a width of ca. 2.5 mm, and a thickness of ca. 0.23 mm were cut out from hot pressed films. The data reported are averages of three independent measurements, and all errors are standard deviations, reported as the variance in a set of samples compared to the mean of the measurement.

### Ultrasonication

All sonication processes were performed in a Sonoswiss s3h bath sonicator (Ramsen, Switzerland).

### X-ray Scattering

Small- and wide-angle X-ray scattering (SAXS/WAXS) measurements were performed on a NanoMax-IQ camera (Rigaku Innovative Technologies, Auburn Hill, MI) equipped with a Cu target sealed tube source (MicroMax 003 microfocus, Rigaku). The scattering spectra were recorded on a Pilatus100 K detector (Dectris). The sample-to-detector distance was calibrated using silver behenate.

### Synthetic Procedures

#### Surface Functionalization of CNCs with BiBB

CNC-Br was synthesized according to the protocol of Zhang.^47^ In a 250 mL round-bottom flask equipped with a magnetic stir bar, CNCs (500 mg) were dispersed in DMF (50 mL) by bath sonication for 1 h. Triethylamine (TEA) (4 mL) and 4-(dimethylamino)pyridine (DMAP) (2 g) were added to the suspension. The suspension was evacuated under vacuum and backfilled with nitrogen three times. The suspension was then placed in an ice bath, and bromoisobutyryl bromide (BiBB) (4 mL) was added dropwise while the mixture was stirred. The reaction mixture was then allowed to warm to room temperature and stirred for 24 h, before ethanol (200 mL) was added to quench the reaction. The suspension was then subjected to centrifugation (7500 rpm, 10 min), and the supernatant was decanted and replaced with ethanol. The centrifugation step was repeated twice, but the supernatant was replaced with THF in the second cycle and deionized H_2_O in the third cycle. After bath sonication (30 min), the CNC-Br suspension was dialyzed in deionized H_2_O for 5–7 days with water exchanges made every day until the pH of the water was 7. The suspension was lyophilized for 3 days using a Telstar Lyoquest at −41.5 °C and at 0.4 mbar, and CNC-Br (200 mg) was obtained as a fluffy solid with a yellow tint. Elemental Analysis: C: 40.05 wt %; H: 4.45 wt %; N: 0.23 wt %; Br: 15.7 wt %.

#### Calculating the Specific Area (SSA) of CNCs and CNC-Br

The SSA of CNCs and CNC-Br was determined using a cylinder model with an ellipsoid cross section according as reported by Lin and Dufresne^48^ by
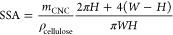
1where *m*_CNC_ represents the mass of the CNCs. The density of cellulose ρ_cellulose_ was assumed to be 1.5 × 10^6^ g m^–3^. The average dimensions of the cotton CNCs were determined by TEM and AFM imaging with an average width (*W*) of 17 nm, a height (*H*) of 4.9 nm, and a length (*L*) of 171 nm (Figures S3 and S4). The SSA of the neat CNCs used in this study was calculated to be 201 m^2^ g^–1^. The average dimensions of CNC-Br were determined by TEM and AFM imaging with an average width (*W*) of 11 nm, a height (*H*) of 5.5 nm, and a length (*L*) of 152 nm (Figures S3 and S4). The SSA of CNC-Br was determined to be 198 m^2^ g^–1^.

### Calculating the Initiator Grafting Density of CNC-Br

Determining the grafting density of the initiator was determined by a protocol by Majoinen et al.^39^ The elemental analysis of CNC-Br resulted in a Br content of 15.7 wt %. The initiator grafting density (σ_i_) was then calculated by using
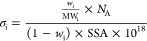
2where *w*_i_ is the weight fraction of Br in CNC-Br (0.0157 g Br/g CNC-Br), MW_i_ is the molecular weight of the grafted initiator species (150 g mol^–1^), *N*_A_ is Avogadro’s number, and the SSA is the specific surface area of CNC-Br in m^2^ g^–1^. This analysis results in a concentration of 3.76 initiator sites nm^–2^ by using the previously determined SSA for CNC-Br (198 m^2^ g^–1^).

### *In Situ* Monitoring of PMA Polymerization

In a 20 mL glass vial, EtBiB (22.01 μL, 0.15 mmol), MA (2.72 mL, 30 mmol), Cu(II)Br_2_ (1.7 mg, 0.008 mmol), and DMSO (2.72 mL) were combined. A 5 cm long copper wire with a diameter of 1 mm was ground with sandpaper, placed in 1 M HCl for 15 min, washed with ethanol and acetone, and wrapped around a magnetic stir bar, and this assembly was added to the reaction flask. After sparging the mixture with N_2_ for 30 min, Me_6_TREN (9.7 μL, 0.04 mmol) was added, and the reaction mixture was stirred under N_2_ at room temperature for 2 h. Every 10 min, a sample (50 μL) was withdrawn with a syringe for NMR characterization. 1 mL of CDCl_3_ was added to each aliquot before being filtered through alumina before ^1^H NMR analysis. After ^1^H NMR experiments, the reaction solutions were dried under vacuum, dissolved in 2 mL of THF, and then used for SEC analysis. Summary of conditions: [MA]:[initiator]:[Cu^II^]:[ligand] of 200:1:0.05:0.25 and solvent:monomer = 1:4 v:v.

### Synthesis of PMA_15_

In a 20 mL glass vial, EtBiB (22.01 μL, 0.15 mmol), MA (2.72 mL, 30 mmol), Cu(II)Br_2_ (1.7 mg, 0.008 mmol), and DMSO (2.72 mL) were combined. A 5 cm long copper wire with a diameter of 1 mm was ground with sandpaper, placed in 1 M HCl for 15 min, washed with ethanol and acetone, wrapped around a magnetic stir bar, and this assembly was added to the reaction flask. After sparging the mixture with N_2_ for 30 min, Me_6_TREN (9.7 μL, 0.04 mmol) was added and the reaction mixture was stirred under N_2_ at room temperature for 1 h. A sample (50 μL) was withdrawn with a syringe for NMR characterization, before the polymerization was quenched by exposing the flask to air and adding 20 mL of THF. The diluted reaction mixture was filtered through silica before being precipitated into cold methanol (250 mL). The product was filtered off and dried in a vacuum oven at 70 °C for 24 h. PMA_15_ (0.65 g, 25% yield) was obtained as a transparent, colorless, and tacky solid. Summary of conditions: [MA]:[initiator]:[Cu^II^]:[ligand] of 200:1:0.05:0.25, solvent:monomer = 1:1 v:v.

### Characterization of PMMA_40_

PMMA was purchased from Sigma-Aldrich. Characterization via ^1^H NMR and SEC can be found in Figures S55 and S56.

### Synthesis of PMA_15_-*b*-PMMA_4_ and PMA_15_-*b*-PMMA_11_

In a 20 mL glass vial, EtBiB (22.01 μL, 0.15 mmol), MA (2.72 mL, 30 mmol), Cu(II)Br_2_ (1.7 mg, 0.008 mmol), and DMSO (2.72 mL) were combined. A 5 cm long copper wire with a diameter of 1 mm was ground with sandpaper, placed in 1 M HCl for 15 min, washed with ethanol and acetone, and wrapped around a magnetic stir bar, and this assembly was added to the reaction flask. After sparging the mixture with N_2_ for 30 min, Me_6_TREN (9.7 μL, 0.04 mmol) was added, and the reaction mixture was stirred under N_2_ at room temperature for 1 h. Then, a mixture of DMSO:MMA (1:1 v:v, 12.8 mL) that had been degassed by sparging with N_2_ for 30 min was added, and the reaction mixture was stirred under N_2_ at room temperature for another 1 h (PMA_15_-*b*-PMMA_4_) or 2 h (PMA_15_-*b*-PMMA_11_). Samples (50 μL) were withdrawn with a syringe for NMR characterization, before the polymerization reactions were quenched by exposing the flasks to air and adding 20 mL of THF. The diluted reaction mixtures were filtered through silica before being precipitated into cold methanol (250 mL). The product was filtered off and dried in a vacuum oven at 70 °C for 24 h. PMA_15_-*b*-PMMA_4_ (1.5 g, 18% yield) and PMA_15_-*b*-PMMA_11_ (1.8 g, 21% yield) were obtained as solid white powders. Summary of conditions: [MA]:[MMA]:[initiator]:[Cu^II^]:[ligand] of 200:400:1:0.05:0.25 and solvent:monomer = 1:1 v:v.

### Synthesis of PMA_8_-*b*-PMMA_7_ and PMA_8_-*b*-PMMA_10_

In a 20 mL glass vial, EtBiB (22.01 μL, 0.15 mmol), MA (1.36 mL, 15 mmol), Cu(II)Br_2_ (1.7 mg, 0.008 mmol), and DMSO (2.72 mL) were combined. A 5 cm long copper wire with a diameter of 1 mm was ground with sandpaper, placed in 1 M HCl for 15 min, washed with ethanol and acetone, and wrapped around a magnetic stir bar, and this assembly was added to the reaction flask. After sparging the mixture with N_2_ for 30 min, Me_6_TREN (9.7 μL, 0.04 mmol) was added, and the reaction mixture was stirred under N_2_ at room temperature for 1 h. Then, a mixture of DMSO:MMA (1:1 v:v, 12.8 mL) that had been degassed by sparging with N_2_ for 30 min was added, and the reaction mixture was stirred under N_2_ at room temperature for another 1 h (PMA_8_-*b*-PMMA_7_) or 2 h (PMA_8_-*b*-PMMA_10_). Samples (50 μL) were withdrawn with a syringe for NMR characterization, before the reactions were quenched by exposing the flasks to air and adding 20 mL of THF. The diluted reaction mixtures were filtered through silica before being precipitated into cold methanol (250 mL). The product was filtered off and dried in a vacuum oven at 70 °C for 24 h. PMA_8_-*b*-PMMA_7_ (2.4 g, 33% yield) and PMA_8_-*b*-PMMA_10_ (2.3 g, 32% yield) were obtained as solid white powders. Summary of conditions: [MA]:[MMA]:[initiator]:[Cu^II^]:[ligand] of 100:400:1:0.05:0.25 and solvent:monomer = 1:1 v:v.

### Synthesis of CNC-*g*-PMA_5_ and CNC-*g*-PMA_8_

In a 10 mL round-bottom flask, CNC-Br (100 mg) and DMSO (10 mL) were combined and sonicated for 30 min to create a 1 wt % suspension. 5 mL of this dispersion (50 mg CNC-Br) was transferred into a round-bottom flask, to which also MA (0.91 mL, 10 mmol) and Cu(II)Br_2_ (1.12 mg, 0.005 mmol) were added. A 5 cm long copper wire with a diameter of 1 mm was ground with sandpaper, placed in 1 M HCl for 15 min, washed with ethanol and acetone, and wrapped around a magnetic stir bar, and this assembly was added to the reaction flask. After sparging the mixture with N_2_ for 30 min, Me_6_TREN (6.48 μL, 0.025 mmol) was added, and the reaction mixture was stirred under N_2_ at room temperature for 1 h (CNC-*g*-PMA_5_) or 2 h (CNC-*g*-PMA_8_) (see Table 1). A sample (50 μL) was withdrawn with a syringe for NMR characterization, before the polymerization was quenched by exposing the flask to air and adding 20 mL of THF. The diluted reaction mixture was transferred to a Falcon flask and centrifuged 5 times at 7500 rpm for 10 min. The supernatant was decanted and replaced with fresh THF after each centrifugation cycle. In order to determine the gravimetric weight gain, the CNC-*g*-PMA was vacuum-dried after the last centrifugation step at 70 °C for 24 h. CNC-*g*-PMA_5_ (∼270 mg, 42 yield%, 16 wt % CNC content) and CNC-*g*-PMA_8_ (∼320 mg, 34 yield%, 13 wt % CNC content) were obtained as a gum-like, white powder. Once dried, CNC-*g*-PMA was redispersed in THF (6 mg mL^–1^), and the suspension was dialyzed in THF with solvent exchanges made every day for 1 week. Summary of conditions: [MA]:initiator]:[Cu^II^]:[ligand] of 100:1:0.05:0.25 and solvent:monomer = 1:4 v:v.

**Table 1:** Composition of Homopolymer and Diblock Copolymer Grafted CNCs and Reaction Conditions Applied

sample name	reaction times PMA/PMMA block (h)	Eq MA/MMA (−)	MA conv^a^(%)	PMA/PMMA in grafts^b^(mol/mol)	PMA/PMMA *M*_n_ (kg mol^–1^)^b^	CNCs (wt %)^c^	PMA wt %/PMMA wt %
CNC-*g*-PMA_5_	1/–	100/0	63	100/0	5/–	9	91
CNC-*g*-PMA_8_	2/–	100/0	83	100/0	8/–	5	95
CNC-*g*-PMA_15_	2/–	200/0	82	100/0	15/–	4	96
CNC-*g*-PMA_8_-*b*-PMMA_2_	2/1	100/400	83	76/24	8/2	1	77/22
CNC-*g*-PMA_8_-*b*-PMMA_7_	2/2	100/400	82	52/48	8/7	2	53/45
CNC-*g*-PMA_5_-*b*-PMMA_7_	1/1	100/400	61	42/58	5/7	2	42/56
CNC-*g*-PMA_5_-*b*-PMMA_12_	1/2	100/400	62	32/68	5/12	2	37/71

a The MA conversion and the *M*_n_ of the PMA block were calculated from the extent of monomer conversion established by *in situ*^1^H NMR spectroscopy of the reaction mixture just before quenching (see text), and the *M*_n_ of the PMMA block was calculated from the PMA/PMMA ratio and the *M*_n_ of the PMA block.

b Determined by ^1^H NMR spectroscopy of isolated products (comparison of the integrals of signals associated with the PMA (3.66 ppm) and PMMA (3.60 ppm) blocks).

c Determined from the weight loss in TGA measurements associated with the degradation of CNCs.

### *In Situ* Monitoring of PMA Growth for CNC-*g*-PMA Polymerization

In a 10 mL round-bottom flask, CNC-Br (100 mg) and DMSO (10 mL) were combined and sonicated for 30 min to create a 1 wt % suspension. 2.5 mL of this dispersion (25 mg of CNC-Br) was transferred into a round-bottom flask, to which also MA (0.91 mL, 10 mmol) and Cu(II)Br_2_ (0.56 mg, 0.003 mmol) were added. A 5 cm long copper wire with a diameter of 1 mm was ground with sandpaper, placed in 1 M HCl for 15 min, washed with ethanol and acetone, and wrapped around a magnetic stir bar, and this assembly was added to the reaction flask. After sparging the mixture with N_2_ for 30 min, Me_6_TREN (3.24 μL, 0.025 mmol) was added, and the reaction mixture was stirred under N_2_ at room temperature for 2 h (Table 1). Every 10 min, a sample (50 μL) was withdrawn with a syringe for NMR characterization. Then, 1 mL of CDCl_3_ was added to each aliquot before being filtered through alumina before ^1^H NMR analysis. Summary of conditions: [MA]:[initiator]:[Cu^II^]:[ligand] of 200:1:0.05:0.25 and solvent:monomer = 1:4 v:v.

### Synthesis of CNC-*g*-PMA_15_

In a 10 mL round-bottom flask, CNC-Br (100 mg) and DMSO (10 mL) were combined and sonicated for 30 min to create a 1 wt % suspension. 2.5 mL of this dispersion (25 mg of CNC-Br) was transferred into a round-bottom flask, to which also MA (0.91 mL, 10 mmol) and Cu(II)Br_2_ (0.56 mg, 0.003 mmol) were added. A 5 cm long copper wire with a diameter of 1 mm was ground with sandpaper, placed in 1 M HCl for 15 min, washed with ethanol and acetone, and wrapped around a magnetic stir bar, and this assembly was added to the reaction flask. After sparging the mixture with N_2_ for 30 min, Me_6_TREN (3.24 μL, 0.025 mmol) was added, and the reaction mixture was stirred under N_2_ at room temperature for 2 h (see Table 1). A sample (50 μL) was withdrawn with a syringe for NMR characterization, before the polymerization was quenched by exposing the flask to air and adding 20 mL of THF. The diluted reaction mixture was transferred to a Falcon flask and centrifuged 5 times at 7500 rpm for 10 min. The supernatant was decanted and replaced with fresh THF after each centrifugation cycle. In order to determine the gravimetric weight gain, the CNC-*g*-PMA was vacuum-dried after the last centrifugation step at 70 °C for 24 h. CNC-*g*-PMA_15_ (∼250 mg, 32% yield, 10 wt % of CNC) was obtained as a gum-like, white powder. Once dried, the CNC-*g*-PMA was redispersed in THF (6 mg mL^–1^), and the suspension was dialyzed in THF with solvent exchanges made every day for 1 week. Summary of conditions: [MA]:[initiator]:[Cu^II^]:[ligand] of 200:1:0.05:0.25 and solvent:monomer = 1:4 v:v.

### Synthesis of CNC-*g*-PMA_8_-*b*-PMMA_2_ and CNC-*g*-PMA_8_-*b*-PMMA_7_

In a 10 mL round-bottom flask, CNC-Br (100 mg) and DMSO (10 mL) were combined and sonicated for 30 min to create a 1 wt % suspension. 5 mL of this dispersion (50 mg of CNC-Br, 5 mL of DMSO) was transferred into a round-bottom flask, to which also MA (0.91 mL, 10 mmol) and Cu(II)Br_2_ (1.12 mg, 0.005 mmol) were added. A 5 cm long copper wire with a diameter of 1 mm was ground with sandpaper, placed in 1 M HCl for 15 min, washed with ethanol and acetone, and wrapped around a magnetic stir bar, and this assembly was added to the reaction flask. After sparging the mixture with N_2_ for 30 min, Me_6_TREN (6.48 μL, 0.025 mmol) was added, and the reaction mixture was stirred under N_2_ at room temperature for 2 h. Then, a mixture of DMSO:MMA (1:1 v:v, 8.6 mL) that had been degassed by sparging with N_2_ for 30 min was added, and the reaction mixture was stirred under N_2_ at room temperature for another 1 h (CNC-*g*-PMA_8_-*b*-PMMA_2_) or 2 h (CNC-*g*-PMA_8_-*b*-PMMA_7_). A sample (50 μL) was withdrawn with a syringe for NMR characterization, before the polymerization was quenched by exposing the flask to air and adding 20 mL of THF. The diluted reaction mixture was transferred to a Falcon flask and centrifuged 5 times at 7500 rpm for 10 min. The supernatant was decanted and replaced with fresh THF after each centrifugation cycle. In order to determine the gravimetric weight gain, the CNC-*g*-PMA-*b*-PMMA was vacuum-dried after the last centrifugation step at 70 °C for 24 h. CNC-*g*-PMA_8_-*b*-PMMA_2_ (430 mg, 52% yield) and CNC-*g*-PMA_8_-*b*-PMMA_7_ (450 mg, 50% yield) were obtained as solid, white powders. Once dried, the CNC-*g*-PMA-*b*-PMMA was redispersed in THF (6 mg mL^–1^), and the suspension was dialyzed in THF with solvent exchanges made every day for 1 week. Summary of conditions: [MA]:[MMA]:[initiator]:[Cu^II^]:[ligand] of 100:400:1:0.05:0.25 and solvent:monomer = 1:4 v:v.

### Synthesis of CNC-*g*-PMA_5_-*b*-PMMA_7_ and CNC-*g*-PMA_5_-*b*-PMMA_12_

In a 10 mL round-bottom flask, CNC-Br (100 mg) and DMSO (10 mL) were combined and sonicated for 30 min to create a 1 wt % suspension. 5 mL of this dispersion (50 mg of CNC-Br, 5 mL of DMSO) was transferred into a round-bottom flask, to which also MA (0.91 mL, 10 mmol) and Cu(II)Br_2_ (1.12 mg, 0.005 mmol) were added. A 5 cm long copper wire with a diameter of 1 mm was ground with sandpaper, placed in 1 M HCl for 15 min, washed with ethanol and acetone, and wrapped around a magnetic stir bar, and this assembly was added to the reaction flask. After sparging the mixture with N_2_ for 30 min, Me_6_TREN (6.48 μL, 0.025 mmol) was added, and the reaction mixture was stirred under N_2_ at room temperature for 1 h. Then, a mixture of DMSO:MMA (1:1 v:v, 8.6 mL) that had been degassed by sparging with N_2_ for 30 min was added, and the reaction mixture was stirred under N_2_ at room temperature for another 1 h (CNC-*g*-PMA_5_-*b*-PMMA_7_) or 2 h (CNC-*g*-PMA_5_-*b*-PMMA_12_). A sample (50 μL) was withdrawn with a syringe for NMR characterization, before the polymerization was quenched by exposing the flask to air and adding 20 mL of THF. The diluted reaction mixture was transferred to a Falcon flask and centrifuged 5 times at 7500 rpm for 10 min. The supernatant was decanted and replaced with fresh THF after each centrifugation cycle. In order to determine the gravimetric weight gain, the CNC-*g*-PMA-*b*-PMMA was vacuum-dried after the last centrifugation step at 70 °C for 24 h. CNC-*g*-PMA_5_-*b*-PMMA_7_ (420 mg, 35% yield) and CNC-*g*-PMA_5_-*b*-PMMA_12_ (690 mg, 40% yield) were obtained as solid, white powders. Once dried, the CNC-*g*-PMA-*b*-PMMA was redispersed in THF (6 mg mL^–1^), and the suspension was dialyzed in THF with solvent exchanges made every day for 1 week. Summary of conditions: [MA]:[MMA]:[initiator]:[Cu^II^]:[ligand] of 200:400:1:0.05:0.25 and solvent:monomer = 1:4 v:v.

### Preparation of Films

Both PMA and CNC-*g*-PMA samples were processed into 250 μm films by hot-pressing at 100 °C at 1 ton of pressure for 2 min between poly(tetrafluoroethylene) sheets. Control of the thickness was done by using 250 μm thick poly(tetrafluoroethylene) as a spacer. For PMMA, PMA-*b*-PMMA, and CNC-*g*-PMA-*b*-PMMA, samples were processed into 250 μm films by hot-pressing at 140 °C at 2 tons of pressure for 2 min between Kapton sheets. Control of the thickness was done by using 250 μm thick aluminum sheets as spacers. The samples were kept in a desiccator to remain dry until mechanical and thermal testing.

## Results and Discussion

The CNCs used in this study were produced by hydrolysis of cotton-based paper with sulfuric acid according to a previously reported protocol.^49^ Conductometric titrations reveal the presence of sulfate half-ester groups (R-OSO_3_H) in a concentration of 115 ± 5 mmol kg^–1^ (Figure S1), thermogravimetric analysis (TGA) data show a 5% weight loss at 215 °C (Figure S2), atomic force microscopy (AFM) images indicate a height of 4.9 ± 3 nm (Figure S3), and the analysis of transmission electron microscopy (TEM) images reveals an average length of 171 ± 26 nm and an average width of 17 ± 3 nm (Figure S4). These data are all typical for cotton-based CNCs that were isolated with the specific protocol that was applied here.^50,51^

**Scheme 1:**
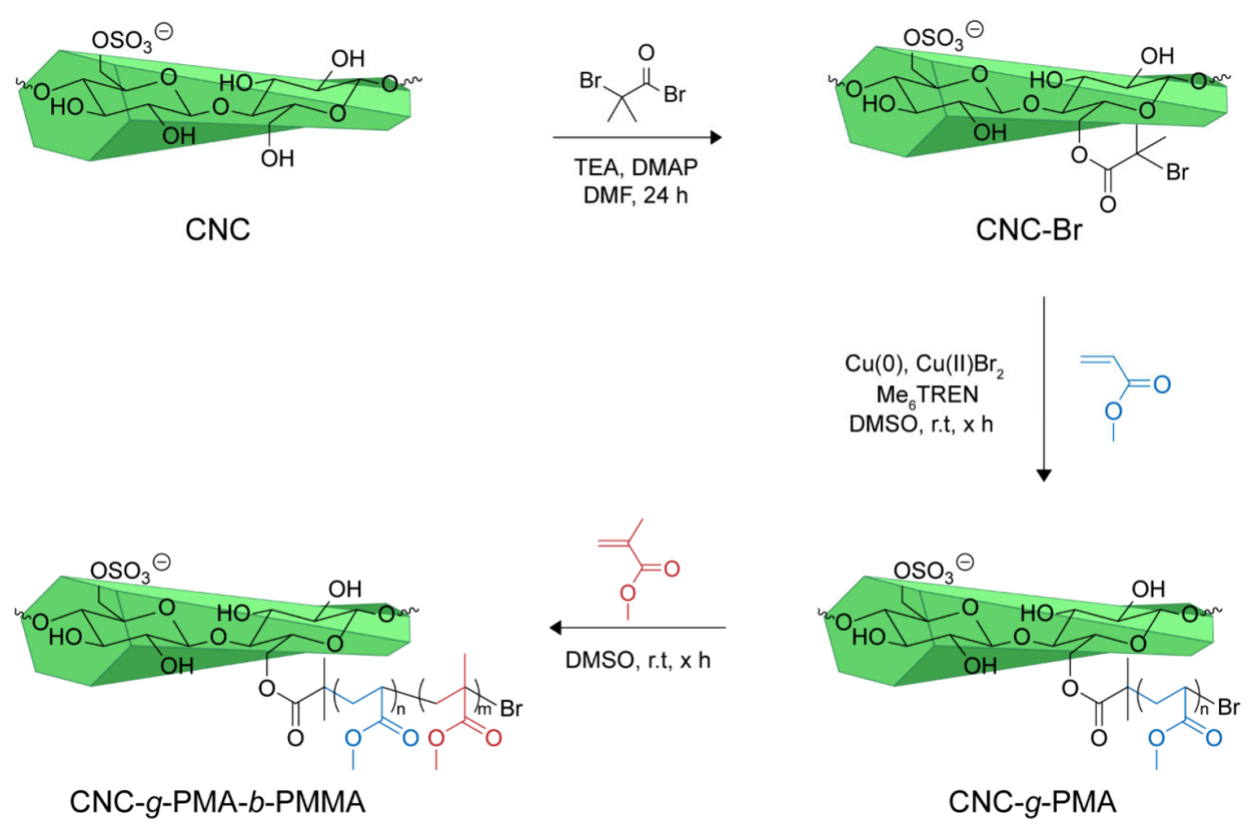
Functionalization of CNCs with α-Bromoisobutyryl-Based ATRP Initiator, Followed by Surface-Initiated SET-ATRP of PMA and Optionally a Second PMMA Block via a Continuous Feed Method

**Figure 2:**
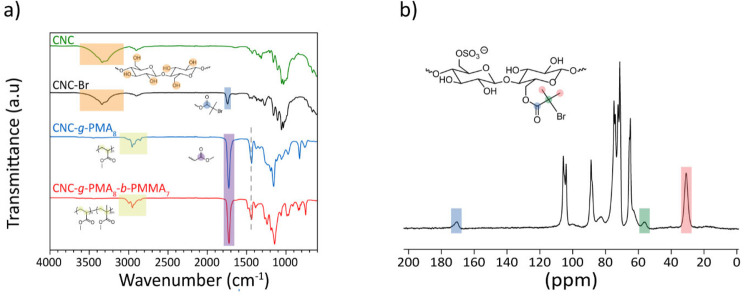
(a) FT-IR spectra of CNCs, CNC-Br, CNC-*g*-PMA_8_, and CNC-*g*-PMA_8_-*b*-PMMA_7_. (b) ^13^C solid-state CP-MAS NMR spectrum of CNC-Br at a spinning frequency 60 kHz and a 5 s relaxation time.

The surface of the CNCs was then decorated with an ATRP initiator based on the bromoisobutyryl ester (BIB) motif, using a modified version of the method reported by Zhang et al.^47^ The protocol involves the esterification of the primary alcohol surface groups with bromoisobutyryl bromide (BIBB, Scheme 1) under basic conditions (see the Experimental Section for details). The CNC-Br thus made have an average length of 152 ± 24 nm, an average width of 11 ± 2 nm, and an average height of 5.5 ± 2 nm (Figures S3 and S4); i.e., their dimensions are very similar to those of the parent CNCs. The successful covalent attachment of the ATRP initiator is supported by the Fourier transform infrared (FT-IR) spectra (Figure 2a), which show the appearance of a band at 1736 cm^–1^ that is diagnostic for the carbonyl (C=O) group of the BIB ester. Other characteristic bands correspond to cellulose OH stretching vibrations at 3450–3050 cm^–1^ and C–O–C stretching at 1162 cm^–1^.^52^ The successful attachment of the initiator to the surface of the CNCs is further confirmed by solid-state ^13^C nuclear magnetic resonance (NMR) spectra, which show the appearance of peaks at 173, 58, and 32 ppm (Figure 2b).

Based on the comparison with reference spectra of the neat CNCs and the reference compound ethyl α-bromoisobutyrate (EtBIB, Figures S5 and S6), the signals can be unequivocally assigned to the carbonyl group, the secondary carbon atom, and the methyl groups of the BIB residue. Elemental analysis of CNC-Br reveals a high Br content of 15.4 ± 0.3 wt %. Based on the molar fraction calculations reported by Majoinen et al.^39^ and the average specific surface area (198 ± 50 m^2^) (eq S1) of CNC-Br determined from the above dimensions using an ellipsoidal cross-section model,^48^ the bromine content translates into an initiator grafting density (σ_i_) of 3.77 ± 0.04 initiator molecules nm^–2^ (eq S2). From the limited number of organic solvents in which CNCs are dispersible,^11^ we selected DMSO, which also dissolves PMA and PMMA, for the grafting reactions. To fine-tune the reaction conditions for this combination of the solvent and monomers, we first homopolymerized methyl acrylate (MA) in DMSO using a reported SET-ATRP protocol and bromoisobutyryl bromide (BiBB) as a soluble initiator.^35,53^ After optimizing the reaction conditions through variation of several parameters, we settled on a molar ratio of [monomer]:[initiator]:[Cu^II^]:[ligand] of 200:1:0.05:0.25, a solvent:monomer ratio of 1:1 v/v, CuBr_2_ as the source of Cu(II), Me_6_TREN as the ligand, and copper wire as the reducing agent. Because the CNC-grafted polymers cannot be characterized by size-exclusion chromatography (SEC), we monitored the monomer conversion by ^1^H NMR spectroscopy. Thus, aliquots were taken from the reaction mixture at different time points, signals associated with the MA’s double bond at 6.36, 6.07, and 5.75 ppm and the PMA’s methyl ester group at 3.66 ppm were integrated, and the number-average molecular weight (*M*_n_) was calculated assuming living conditions using eqs S3 and S4 (Figure S8–S12). The data show that within 1 h, 90% of the monomer is converted into PMA (Figure S10 and Table S1), which translates into an *M*_n_ of 15.9 kg mol^–1^. The *M*_n_ values established for different reaction times by SEC perfectly match the values determined by NMR, and the low dispersity (*Đ*) of <1.08 confirms the living nature of the reaction (Figures S11 and S12). We also performed the preparative synthesis of a model PMA, stopping the reaction after 1 h and isolating the polymer. This afforded PMA with an *M*_n_ of 15.7 kg mol^–1^ and a *Đ* of 1.08 (Figures S9 and S12,Table S1); we refer to this sample as PMA_15_.

PMA-*b*-PMMA diblock copolymers were made similarly, but a continuous feed method was used. Initially, we employed a [MA]:[MMA]:[initiator]:[Cu^II^]:[ligand] ratio of 200:400:1:0.05:0.25, starting the reaction in the absence of MMA and with a DMSO:MA ratio of 1:1 v/v, i.e., using the same conditions as employed for the synthesis of PMA. After a reaction time of 1 h, i.e., when a PMA block of an *M*_n_ of ca. 15 kg mol^–1^ had formed, a degassed 1:1 v:v MMA:DMSO mixture was added, and the reaction was allowed to proceed for another 1 or 2 h. Just before quenching, aliquots were taken for NMR analysis (Figures S13–S15). The reaction mixture was then precipitated into cold methanol; the solidified PMA-*b*-PMMA was filtered off, dried overnight, and dissolved in CDCl_3_ for ^1^H NMR experiments. Through the comparison of the integrals of ^1^H NMR signals associated with the PMA (3.66 ppm) and PMMA (3.60 ppm) blocks, the composition of the copolymers (PMMA molar fractions of 25 and 39%) was determined. The *M*_n_ of the PMMA block (4 and 11 kg mol^–1^) was then calculated from the composition and the *M*_n_ of the PMA block (Table S1, Figures S16 and S17). On this basis, the polymers isolated after reaction times of 1 + 1 h and 1 + 2 h are designated as PMA_15_-*b*-PMMA_4_ and PMA_15_-*b*-PMMA_11_, respectively, where the subscripts indicate the *M*_n_ of the two blocks in 1000 g mol^–1^. To increase the PMMA fraction, the PMA block was shortened by reducing the initial MA concentration to one-half, while all other conditions were kept the same. NMR experiments show that after 1 h of reaction (with only MA present), 90% of the monomer had been converted, and an MA block with an *M*_n_ of 7.9 kg mol^–1^ had formed (Figure S18). Subsequent MMA addition and polymerization for 1 or 2 h afforded PMMA blocks with an *M*_n_ of 7.2 or 9.9 kg mol^–1^ (Figures S19–S22). The isolated polymers are designated as PMA_8_-*b*-PMMA_7_ and PMA_8_-*b*-PMMA_10_ (Table S1, Figures S21 and S22). The SEC traces of the isolated block copolymers all show bimodal molecular weight distributions (Figure S13). In the case of PMA_15_-*b*-PMMA_4_ and PMA_15_-*b*-PMMA_11_, a low-*M*_n_ peak is observed that is not or only slightly increased vis-á-vis the *M*_n_ of the PMA block. In the case of PMA_15_-*b*-PMMA_4_, the second peak is narrow, and its integration affords an *M*_n_ of ca. 41.8 kg mol^–1^. In the case of PMA_15_-*b*-PMMA_4_, the second peak is considerably broadened, and its separate integration reflects an *M*_n_ of ca. 51.2 kg mol^–1^. The SEC traces of PMA_8_-*b*-PMMA_7_ and PMA_8_-*b*-PMMA_10_ show similar features. Thus, while the SEC data confirm the formation of block copolymers, they also show that control is lost and that only a fraction of chains propagate upon adding MMA.

**Figure 3:**
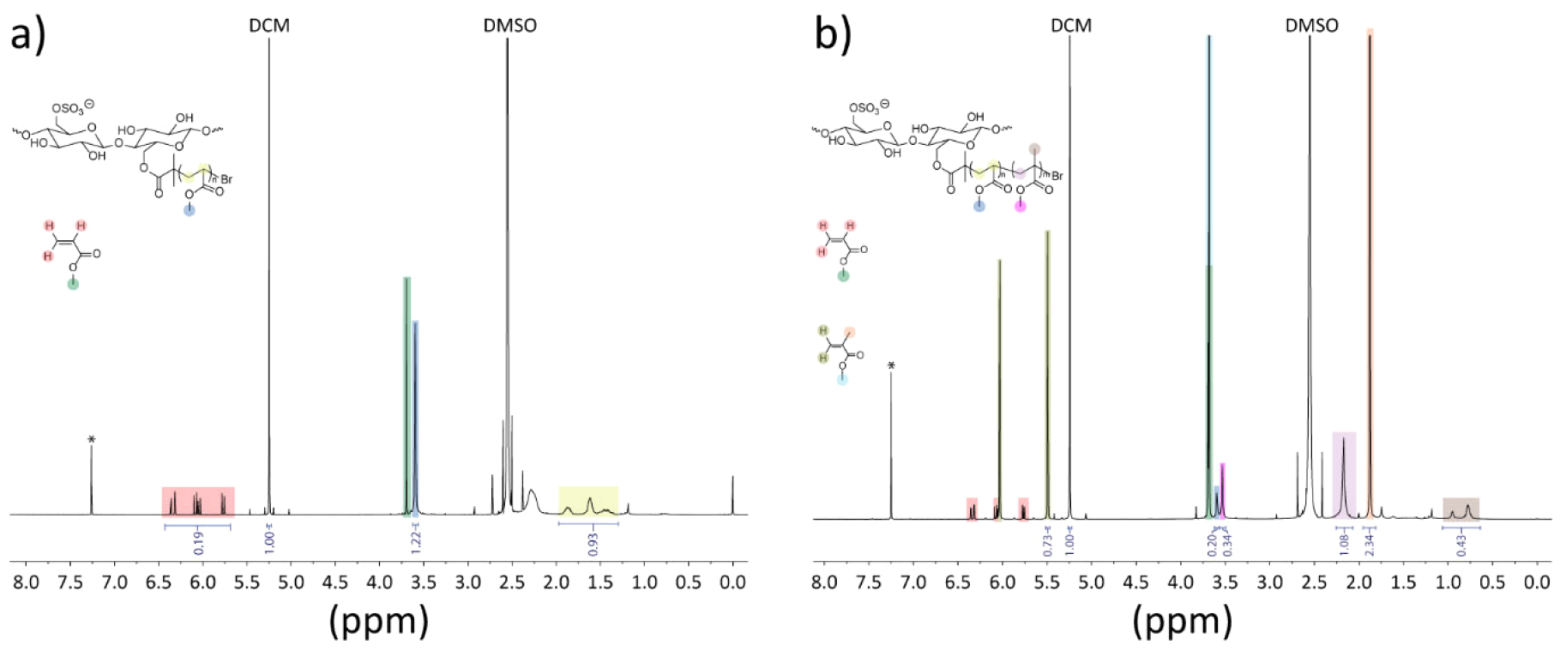
^1^H NMR spectra of aliquots that were taken from the reaction mixtures to produce (a) CNC-*g*-PMA_15_ and (b) CNC-*g*-PMA_5_-*b*-PMMA_12_ just before the reactions were quenched. The spectra were recorded in CDCl_3_.

Next, we prepared a series of CNCs grafted with PMA only (CNC-*g*-PMA_*x*_). This was accomplished by adapting the conditions developed for PMA but employing CNC-Br instead of BiBB. Intriguingly, solution-phase ^1^H NMR spectra clearly show the signals of the polymer grown from the CNCs (Figure 3a and Figures S23–S26), which is likely related to the high grafting density and reflects that the growing polymer chains are well solvated.^44^ Thus, the monomer conversion could be monitored with the same NMR technique as the free polymers. Note that this method afforded the same monomer conversion results, as obtained by comparing the integrals of ^1^H NMR signals of the monomer and an auxiliary standard (Table S3, eq S5, and Figures S27–S31).^36^ In an initial experiment, the same reaction conditions as detailed above for the synthesis of PMA_15_ were used. A comparison of the conversion vs time plots (Figure S10) shows that the reaction rate is reduced compared to the model reaction in solution, and a longer reaction time (2 h) is required to reach 90% monomer conversion. This effect is well-known for SI-ATRP reactions and is related to the heterogeneous nature of the reaction and the steric crowding on the surfaces of the CNCs, which likely prevents the growth of PMA from all initiator sites.^34,47,54^ Consequently, the *M*_n_ values determined from the extent of conversion after a reaction time of 2 h (15 kg mol^–1^, the isolated material is termed CNC-*g*-PMA_15_, Table 1) likely underestimates the actual value. The increase of the reaction mixture’s viscosity was much more pronounced than in the case of the CNC-free model reactions, which we interpret with entanglements between the brush-like particles formed in the reaction. When the monomer concentration was increased to 500 or 1000 equiv relative to the initiator, the reaction mixtures gelled within 30 min; the *M*_n_ values determined from the conversions are 20 and 35 kg mol^–1^, respectively (Figures S23 and S24). By contrast, the viscosity decreased when the monomer concentration was reduced to 100 equiv. This concentration and reaction times of 1 or 2 h were used to produce CNC-*g*-PMA grades with shorter grafts (CNC-*g*-PMA_5_ and CNC-*g*-PMA_8_, Figures S25 and S26). The different CNC-*g*-PMAs were isolated, just after the NMR samples to determine the conversion were taken, by multiple centrifugation and washing steps with THF and subsequent drying. The yields of 34–46%, calculated from the weight of the isolated materials, the amount of CNC-Br employed, the conversion determined by NMR, and the quantity of PMA that should result suggest that this process can be optimized. The losses prevent the gravimetric determination of the CNC content, which was therefore calculated from the weights of the starting materials and the conversion (3–8 wt %, Experimental Section). All CNC-*g*-PMA grades can be readily suspended in solvents that dissolve PMA but in which the parent CNCs aggregate, such as chloroform and THF. This was exploited to purify the materials through redispersion in THF and dialysis against THF. The purified materials were redispersed in CDCl_3_ to acquire ^1^H NMR spectra that confirm the structure of the grafted polymers (Figures S32–S34). The presence of the surface-grafted polymer is also confirmed by FT-IR spectra (Figure 2a and Figure S48), which display signals characteristic of the polymer.

The solid-state ^13^C NMR spectrum of dried CNC-*g*-PMA_15_ shows evidence of PMA grafting, with peaks at 176 ppm, corresponding to the carbonyl groups, as well as at 55 and 40 ppm corresponding to the carbons on the polymer backbone. (Figure S7). In addition, CNC-*g*-PMA samples were redispersed in THF at a concentration of 0.1 wt % in order to elucidate their morphology by TEM. The TEM images reveal an average length of 185 ± 31 nm and an average overall width of 31.8 ± 7 nm (Figure S35). Rod-shaped particles resembling CNCs appear to be surrounded with a material that extends from both sides with an average length of 10.4 ± 1 nm, which is consistent with the grafted PMA.

Block copolymer-grafted CNCs (CNC-*g*-PMA-*b*-PMMA) were prepared through a continuous feed method, as was applied to synthesize the PMA-*b*-PMMA reference diblock copolymers discussed above. We initially adopted the reaction conditions used for CNC-*g*-PMA_15_ and added a degassed mixture of MMA (400 equiv relative to the initiator) and DMSO (1:1 v/v) after 1 h, but the reaction mixture gelled shortly after the MMA addition, presumably on account of excessive chain entanglements. We therefore reduced the MA concentration to the level employed for the synthesis of CNC-*g*-PMA_8_ and CNC-*g*-PMA_5_ (100 equiv relative to the initiator) so that the molar ratios were [MA]:[MMA]:[initiator]:[Cu^II^]:[ligand] = 100:400:1:0.05:0.25. We first prepared two compositions in which the MA polymerization was performed for 2 h, leading to a monomer conversion of 90% and a PMA block length of 7.6 kg mol^–1^ (determined by ^1^H NMR spectroscopy of the reaction mixtures) before the MMA/DMSO mixture was added, and the reaction was continued for 1 h to produce CNC-*g*-PMA_8_-*b*-PMMA_2_ or 2 h to produce CNC-*g*-PMA_8_-*b*-PMMA_7_. Also, in these cases, solution-phase ^1^H NMR spectra show the signals of the polymer grown from the CNCs (Figure 3b, Figures S36 and S37), but high viscosity and eventual gelation of the reaction mixture led to difficulties in collecting aliquots from all reactions to monitor the growth of the PMMA block. Therefore, the materials were isolated and purified following the same procedure applied for CNC-*g*-PMA. ^1^H NMR spectra of the redispersed materials were used to determine the molar ratio of PMA to PMMA through the integration of the methyl ester signals for PMA (3.66 ppm) and PMMA (3.60 ppm). This value was used to estimate the *M*_n_ of the PMMA block from the *M*_n_ of the PMA block, which for each reaction was determined by ^1^H NMR spectroscopy (Table 1, Figures S38 and S39). To create HNPs with a higher PMMA fraction, the PMA block was again shortened to ca. 5 kg mol^–1^ by reducing the initial MA concentration (Figures S40 and S41), and subsequent MMA addition and polymerization for 1 or 2 h afforded PMMA blocks with an *M*_n_ of 6.5 or 11.5 kg mol^–1^ (CNC-*g*-PMA_5_-*b*-PMMA_7_ and CNC-*g*-PMA_5_-*b*-PMMA_12_, Figures S42 and S43). Also, for these materials, FT-IR spectra show signals that evidence the presence of the polymer grafts and the CNCs (Figure 2a and Figure S48). Based on the MA conversion and the MA:MMA ratio in the grafts (Table 1), we estimate the CNC content in these OCNs to be below 6 wt %. The solid-state ^13^C NMR spectrum of CNC-*g*-PMA_5_-*b*-PMMA_12_ confirms the growth of PMMA chains, with peaks at 176 ppm, corresponding to the carbonyl groups, as well as at 55, 40, and 20 ppm, corresponding to the carbons of the polymer backbone (Figure S7). The TEM images reveal an average length of 188 ± 21 nm and an average width of 33.5 ± 8 nm (Figure S35). Also in this case, the images show rod-shaped particles that resemble CNCs and are surrounded by polymeric material that extends from both sides of the CNCs with an average length of 13.8 ± 1 nm. The slightly larger width in comparison to CNC-*g*-PMA (+ 2 nm) is consistent with the presence of the additional PMMA segment. Small- and wide-angle X-ray (SAXS, WAXS) scattering data of CNC-Br, CNC-*g*-PMA, and CNC-*g*-PMA-*b*-PMMA show characteristic CNC peaks and evidence of amorphous polymers (Figure S57). While the diffraction pattern of CNC-Br shows clear peaks in the WAXS regime that reflect the crystalline nature of the CNCs, these signals are barely visible in the patterns of the polymer-grafted CNCs, which are dominated by broad halos that are characteristic of amorphous polymers.

**Figure 4:**
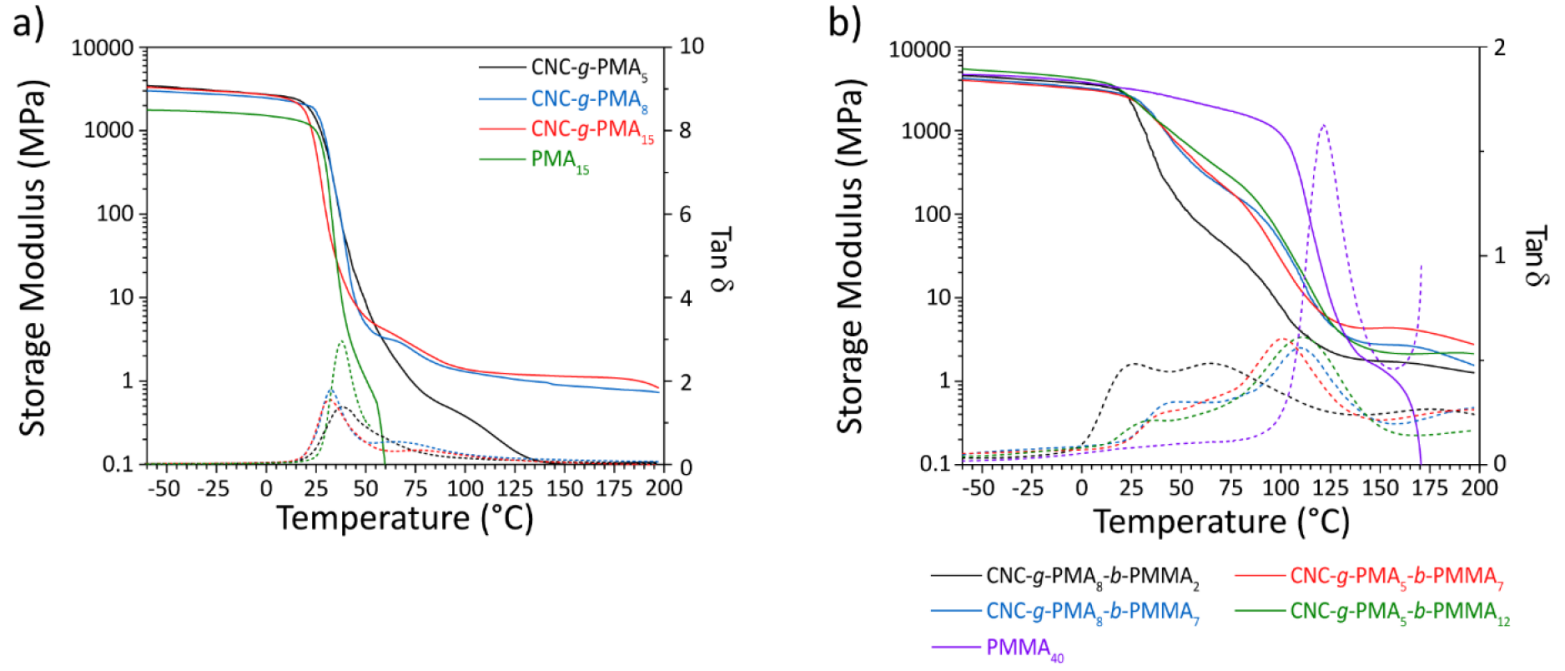
Dynamic mechanical analysis traces of OCNs based on (a) the CNC-*g*-PMA and (b) the CNC*-g*-PMA-*b*-PMMA series. Data for free PMA (a) and PMMA (b) reference polymers are also shown.

**Figure 5:**
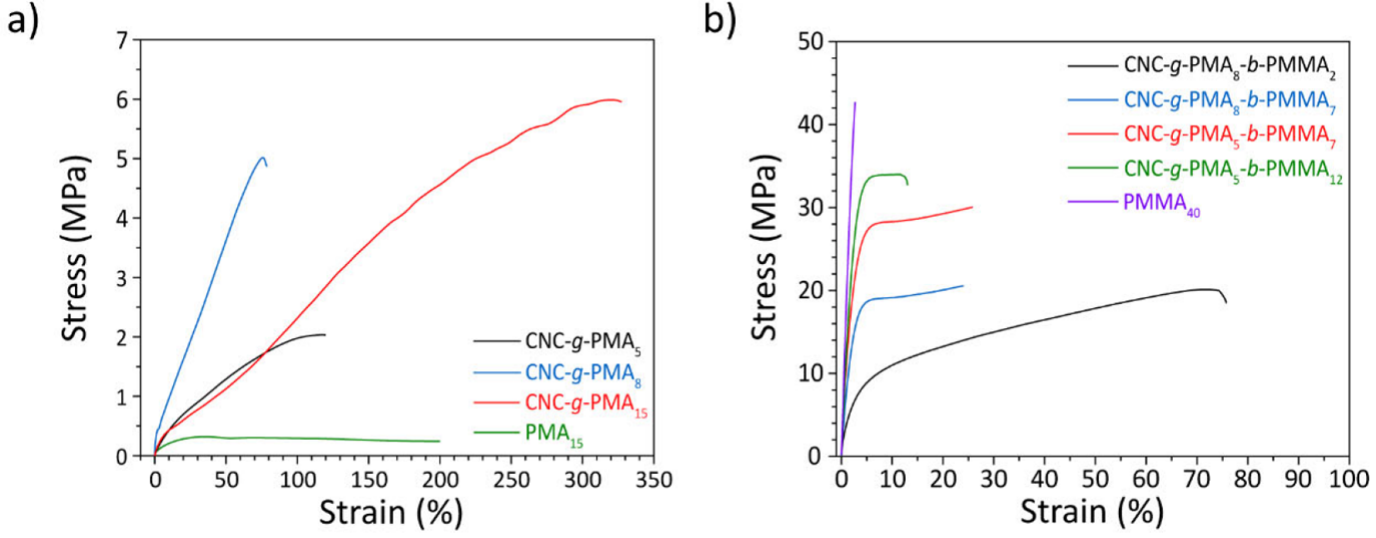
Tensile tests of OCNs based on (a) the CNC-*g*-PMA and (b) the CNC*-g*-PMA-*b*-PMMA series. Data for free PMA (a) and PMMA (b) reference polymers are also shown.

The PMA reference polymers and CNC-*g*-PMA were processed into 250 μm thin films by hot-pressing at 100 °C, whereas for PMMA, PMA-*b*-PMMA, and CNC-*g*-PMA-*b*-PMMA a temperature of 140 °C was applied. The films thus made were highly transparent, except for the CNC-*g*-PMA series, which were initially clear but became hazy within minutes after cooling (Figures S50 and S51). The thermomechanical behavior of the materials was probed by dynamic mechanical analysis (DMA) of thin films in tension mode. The DMA trace of the CNC-free PMA reference shows a glassy regime with a storage modulus (*E*′) typical of an amorphous glassy polymer (2.6 GPa at −60 °C, Figure 4a and Table 2). *E*′ starts to decrease around *T*_g_, observed at 37 °C as a maximum of the corresponding loss tangent (tan δ) function. Above this temperature, *E*′ drops rapidly and the sample fails around 60 °C. The DMA trace of CNC-*g*-PMA_5_ shows similar features, but due to the presence of the CNCs, *E*′ is increased to 3.5 GPa in the glassy regime. Moreover, *E*′ decreases more gradually, and the failure temperature is increased to ca. 135 °C, which we relate to the onset of entanglements between relatively short PMA chains grafted to different CNCs. This effect becomes more prominent in the OCNs based on CNCs with longer PMA grafts, i.e., CNC-*g*-PMA_8_ and CNC-*g*-PMA_15_. The DMA traces of these materials are practically identical and show a rubbery plateau with *E*′ ≈ 1–2 MPa that extends to 200 °C (Figure 4a and Table 2). We recall that these materials are not chemically cross-linked and that the CNC content is below the percolation concentration. Thus, the DMA data support the conclusion that mechanical stress transfer above *T*_g_ is primarily related to chain entanglements between the polymer-grafted CNCs. The DMA traces of the OCNs based on block copolymer-grafted CNCs (CNC-*g*-PMA-*b*-PMMA) also show an extended rubbery plateau, in contrast to the reference block copolymers, in which entanglements are limited (Figure 4b and Figure S52). The DMA traces of the block copolymer OCNs further reveal two thermal transitions that reflect microphase separation of the two blocks. Thus, *E*′ drops in two distinct steps whose relative magnitudes scale roughly with the fractions of the two blocks (Table 1). The maxima of the tan δ traces (i.e., the *T*_g_ values) and the magnitude of the respective signals provide further support for this morphology. Thus, CNC-*g*-PMA_8_-*b*-PMMA_2_, which features the lowest PMMA content, displays a first *T*_g_ of 39 °C, which is slightly higher than that of PMA_15_ and of materials of the CNC-*g*-PMA series (Table 2), likely on account the formation of small PMA domains that are confined between the CNCs and glassy PMMA domains. The PMMA phase displays a *T*_g_ of 112 °C, which is considerably lower than that of the neat PMMA and the block copolymer references, reflecting the incorporation of some MA through the gradient process and likely the formation of small, confined domains. The modulus reduction observed for this material above the PMA *T*_g_ is the most pronounced among the series of block copolymer–OCNs (*E*′ is reduced from 4390 MPa at −60 °C to 37 MPa at 75 °C), consistent with the high PMA fraction in this material (76%). As the PMMA fraction is increased, the *T*_g_ values shift to higher temperatures, and the modulus drop above the PMA *T*_g_ is less pronounced. Thus, CNC-*g*-PMA_8_-*b*-PMMA_7_ (52% PMA) shows *T*_g_s at 50 and 114 °C, and *E*′ at 75 °C is 178 MPa (Figure 4b and Table 2). CNC-*g*-PMA_5_-*b*-PMMA_7_, which has a slightly higher PMMA fraction (58%), shows similar properties, while CNC-*g*-PMA_5_-*b*-PMMA_12_ with the highest PMMA content (68%) displays the highest stiffness. The mechanical properties of the OCNs were further investigated by uniaxial tensile tests that were performed with a strain rate of 50% min^–1^ at room temperature, i.e., near the PMA *T*_g_. Consequently, the neat PMA (PMA_15_) exhibits a low ultimate tensile strength (σ_UTS_ = 0.2 MPa), a low Young’s modulus (*E*_y_ = 9 MPa), and a high elongation at break (ε_B_ = 200%) (Figure 5a and Table 2). Consistent with the low CNC content, *E*_y_ is only slightly higher in the CNC-*g*-PMA OCNs (11–55 MPa), but these materials show strain hardening, and σ_UTS_ is increased by an order of magnitude or more to 2–6 MPa, growing with the PMA block length. Intriguingly, unlike in conventional two-component CNC nanocomposites,^6,8^ a high elongation at break (72–320%) is retained, which we relate to the fact that the stress transfer does not involve a percolating, hydrogen-bonded CNC network, but instead entanglements between the polymer grafts. Consequently, CNC-*g*-PMA_15_ displays the highest and CNC-*g*-PMA_5_ the lowest extensibility. We relate the fact that CNC-*g*-PMA_8_ displays the steepest stress–strain curve (Figure S53) and the lowest ε_B_ to the slightly different *T*_g_s of the materials, as supported by the DMA traces, which show that the temperature at which the modulus starts to trop is highest for CNC-*g*-PMA_8_. The stress–strain curves of the CNC-*g*-PMA-*b*-PMMA series reveal that the introduction of the PMMA blocks leads to further and very significant increases in strength and stiffness (Figure 5b and Table 2). Both, *E*_y_ and σ_UTS_ increase steadily with the PMMA fraction, while ε_B_ is decreased. Across the board, the *E*_y_ and σ_UTS_ values are higher than those of the CNC-free reference copolymers (Figure S54) and reach values of *E*_y_ = 1.6 GPa and σ_UTS_ = 34 MPa for CNC-*g*-PMA_5_-*b*-PMMA_12_, in which the PMMA fraction is 68%. Except for CNC-*g*-PMA_8_-*b*-PMMA_2_, the stress–strain curves show a yield point, above which the deformation becomes plastic. Notably, although the strain at break decreases with the PMMA content, the block copolymer OCNs are all ductile with ε_B_ = 20–80%, in contrast to the PMMA-rich reference diblock copolymers PMA_8_-*b*-PMMA_7_ and PMA_8_-*b*-PMMA_10_ (Figure S54) and the neat PMMA (Figure 5b), which display brittle failure (Table S2). Intriguingly, the OCN with the highest PMMA content, CNC-*g*-PMA_5_-*b*-PMMA_12_, displays a Young’s modulus that is comparable to the one of PMMA (1.7 GPa) but a 2-fold higher strain at break (11%) and a much higher toughness (3.2 MJ m^–3^ vs 1.1 MJ m^–3^), even though *E*_y_ is somewhat reduced (34 instead of 47 MPa). The toughness is up to an order of magnitude higher than that of previously reported OCNs based on CNCs grafted with PMMA homopolymers (0.34–0.61 MJ m^–3^),^29^ which highlights the importance of the rubbery PMA blocks that toughen the material though elastic energy dissipation.

**Table 2:** Mechanical and Thermomechanical Properties of All OCNs and Reference Homopolymers

sample	*E*_y_^a^ (MPa)	σ_UTS_^a^ (MPa)	ε_B_^a^ (%)	tensile toughness^a^ (MJ m^–3^)	*E*′ at −60 °C^b^ (MPa)	*T*_g,1_^b^ (° C)	*T*_g,2_^b^ (° C)
PMA_15_	9 ± 0.6	0.15 ± 0.0	265 ± 29	0.17 ± 0.0	2590 ± 380	37 ± 1	
PMMA_40_	1770 ± 280	47 ± 7	4 ± 1	1.1 ± 0.4	4700 ± 120		120 ± 1
CNC-*g*-PMA_5_	14 ± 3	2.1 ± 0.3	113 ± 15	1.7 ± 0.5	3445 ± 140	37 ± 1	
CNC-*g*-PMA_8_	55 ± 6	3.8 ± 1	72 ± 15	2.0 ± 0.4	3230 ± 80	31 ± 2	
CNC-*g*-PMA_15_	11 ± 3	5.7 ± 0.3	320 ± 32	11.2 ± 0.9	3270 ± 70	32 ± 1	
CNC-*g*-PMA_8_-*b*-PMMA_2_	360 ± 20	19 ± 1	80 ± 6	11.6 ± 0.9	4390 ± 160	39 ± 3	112 ± 5
CNC-*g*-PMA_8_-*b*-PMMA_7_	920 ± 32	21 ± 1	19 ± 5	3.5 ± 0.9	4160 ± 500	50 ± 7	114 ± 1
CNC-*g*-PMA_5_-*b*-PMMA_7_	1300 ± 70	28 ± 2	21 ± 6	5.3 ± 1.8	4230 ± 250	48 ± 1	105 ± 1
CNC-*g*-PMA_5_-*b*-PMMA_12_	1590 ± 50	34 ± 1	11 ± 3	3.2 ± 0.8	4970 ± 700	42 ± 9	111 ± 1

a Determined by tensile tests.

b Determined by dynamic mechanical analysis; *T*_g_ values represent the maxima of the tan δ curves

## Conclusion

In summary, novel one-component nanocomposites based on CNCs grafted with homopolymers and diblock copolymers in SET-ATRP reactions are reported. The grafted nanoparticles disperse readily in organic solvents, and the polymer grafts are well solvated, which enables their characterization by *in situ* or *ex situ*^1^H NMR spectroscopy. This capability allows monitoring the growth of the polymers and can be used to determine the composition of the HNPs as they are produced. We note that the model reactions show that upon addition of MMA, the living nature of the polymerization is compromised, and consequently, the *M*_n_ of the PMMA block is systematically underestimated. Nevertheless, the method is clearly well-suited to establish the fractions of the two blocks, which appears to have a much larger influence on the mechanical characteristics than the block length *per se*. Dynamic mechanical analysis shows that the HNPs made assemble into microphase-separated OCNs that display two distinct glass transitions associated with PMA and PMMA rich phases. This in turn demonstrates that the continuous feed method applied affords block copolymer grafts with fairly well-defined blocks. Thermomechanical testing also suggests a densely entangled morphology, as the CNC-*g*-PMA and CNC-*g*-PMA-*b*-PMMA OCNS do not mechanically fail above *T*_g_. Instead, the DMA traces show extended and robust rubbery plateaus reminiscent of chemically cross-linked networks. Tensile tests show that the OCNs are superior in strength, ductility, and modulus compared to the free copolymers, which makes them useful for applications in which a combination of transparency and toughness is required. The data highlight the importance of the rubbery PMA blocks in toughening the material through elastic energy dissipation. We concede that the *T*_g_ of PMA, close to ambient temperature and shifting somewhat between the various materials, is not ideal, as small temperature changes may significantly change the mechanical properties of the OCNs.

## Supporting Information Available

Titration data, TGA data, and AFM and TEM images of the CNCs, NMR spectra of all polymer-grafted CNCs, additional size-exclusion chromatography, TGA, IR, and mechanical data of the polymer-grafted CNCs, and tables in which reaction conditions and compositions of reference polymers are compiled (PDF)
