# Supplementary material for: One-Component Nanocomposites Made from Diblock Copolymer Grafted Cellulose Nanocrystals
Source: Biomacromolecules. 2024 Feb 21;25(3):1637–48. doi: 10.1021/acs.biomac.3c01196 (PMC10934803; doi:10.1021/acs.biomac.3c01196)
Supplement: Supplementary file 1 — bm3c01196_si_001.pdf [file bm3c01196_si_001.pdf]

Supporting Information

One-component nanocomposites made from diblock copolymer grafted cellulose nanocrystals

Chris Rader[1], Patrick W. Fritz[2], Timur Ashirov[2], Ali Coskun[2], Christoph Weder[1,]*

[1]Adolphe Merkle Institute, University of Fribourg, Chemin des Verdiers 4, 1700 Fribourg, Switzerland

[2]Department of Chemistry, University of Fribourg, Chemin de Musee 9, 1700 Fribourg, Switzerland

Table of Contents

1 Supplementary Figures S1-57 2
2 Supplementary Tables S1-3 48
3 Supplementary Equations S1-5 50

Supplementary Figures S1-57

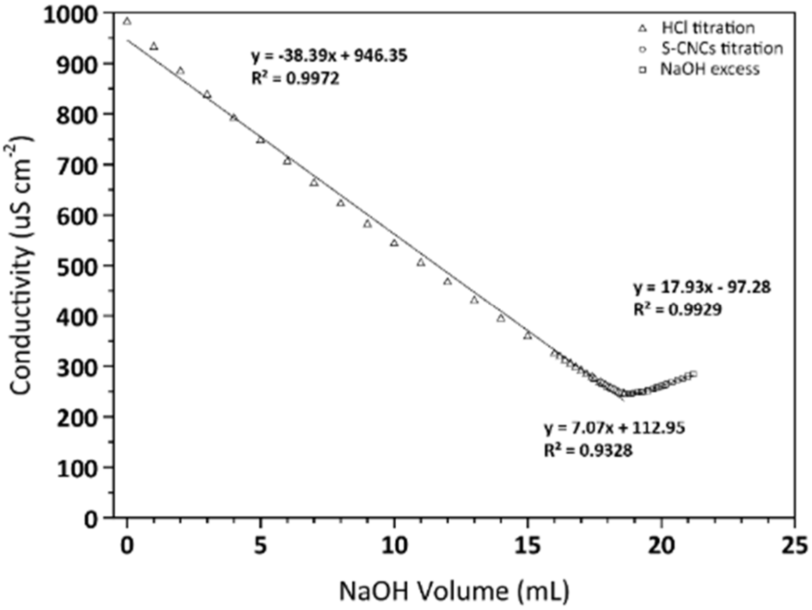

Figure S1. Conductometric titration curve for S-CNCs titrated against 0.01M NaOH solution. The two equivalent points determined graphically were used to calculate the surface half ester content. Results show a concentration of 115 ± 5 mmol kg^{-1} sulfate-half ester groups (**R-OSO_3H**).

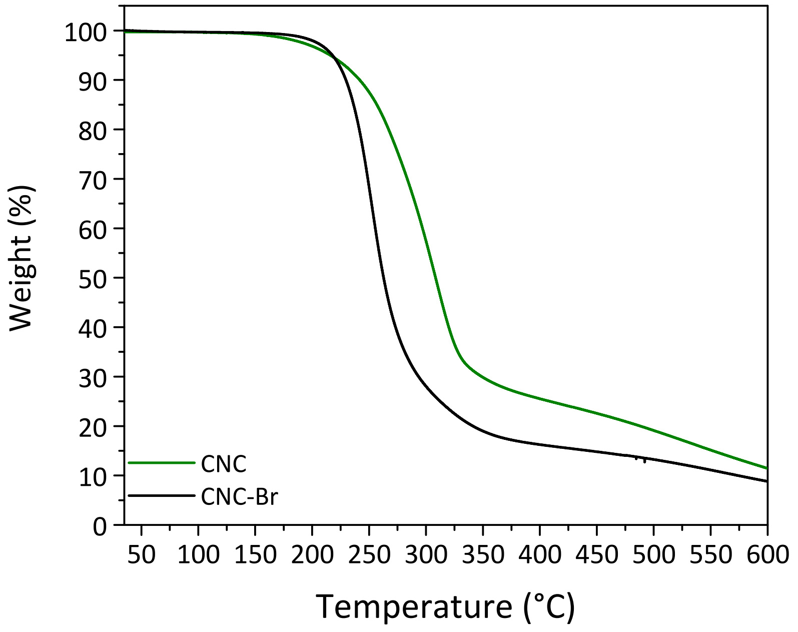

Figure S2. Thermogravimetric analysis traces of CNC and CNC-Br recorded at a heating rate of 10 °C min^{-1} showing an onset of degradation for CNC at 150 °C and for CNC-BR at 200 °C.

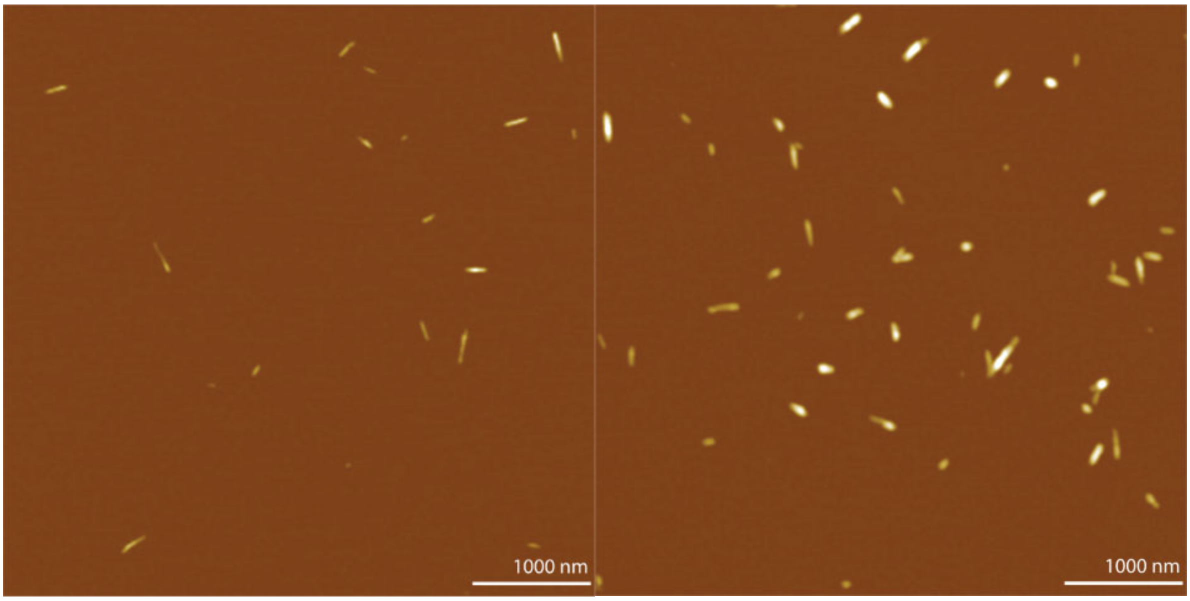

Figure S3. AFM images of CNCs (left) indicating an average height of 4.9 ± 3 nm and CNC-Br (right) showing an average height of 5.5 ± 2 nm.

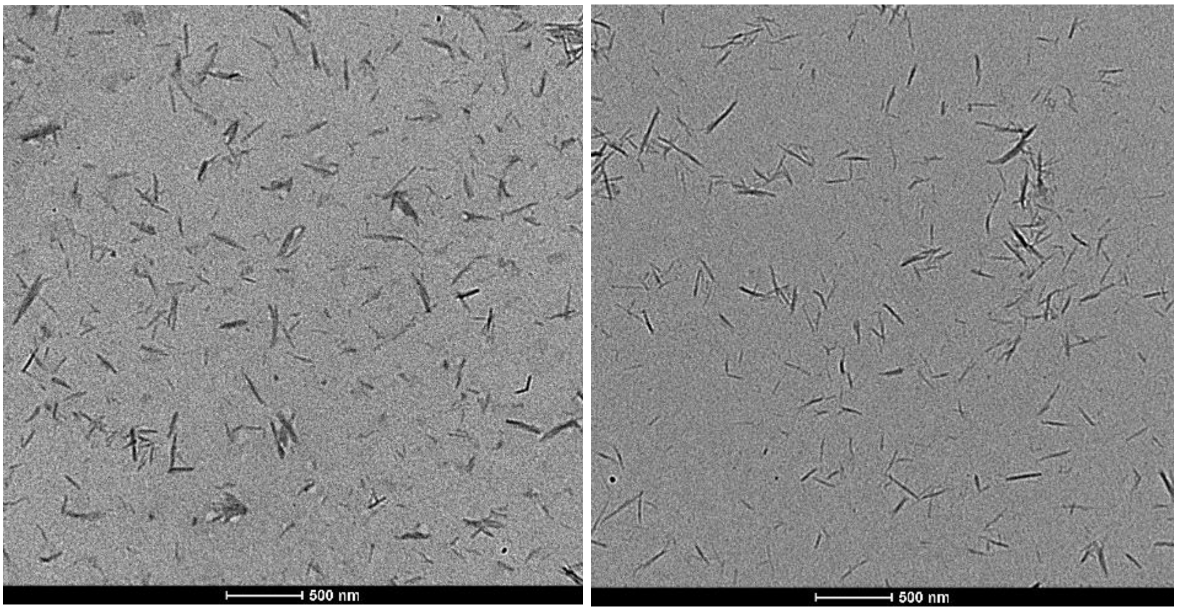

Figure S4. TEM micrographs of a representative spot of a spin-coated 0.001 wt% aqueous dispersion of neat CNCs, scale bar = 500 nm (left) an average length of 171 ± 26 nm and an average width of 17 ± 3 nm and of a spin-coated 0.001 wt% DMSO dispersion of CNC-Br, scale bar = 500 nm (right) an average length of 152 ± 24 nm, an average width of 11 ± 2 nm, and an average height of 5.5 ± 2 nm.

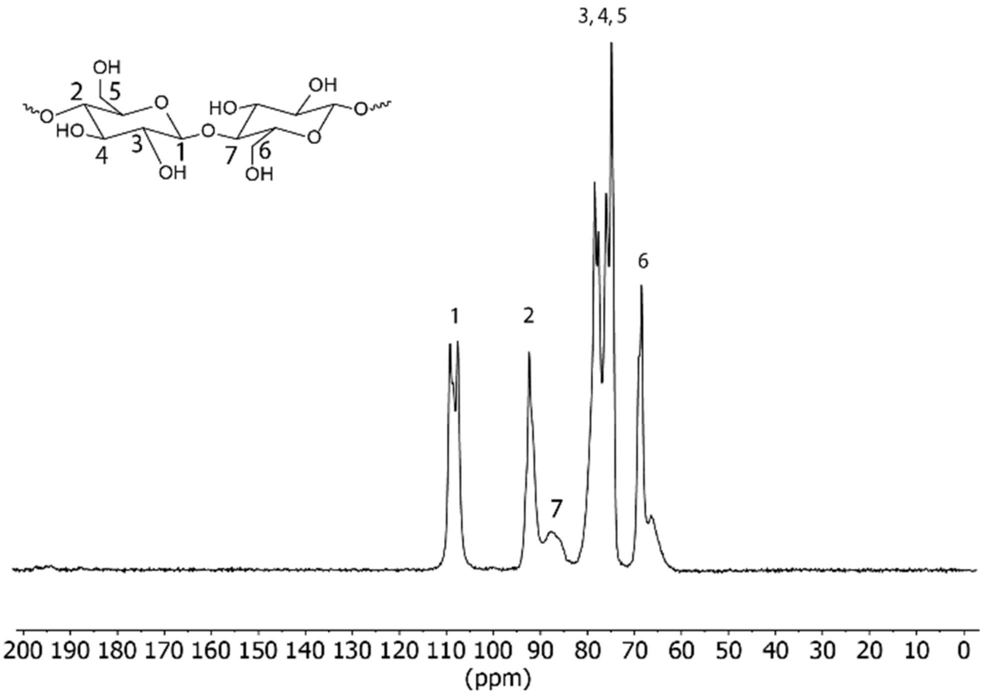

Figure S5. ^{13}C solid-state CP-MAS NMR spectrum of CNCs recorded at a spinning rate of 60 kHz with 10 s relaxation time.

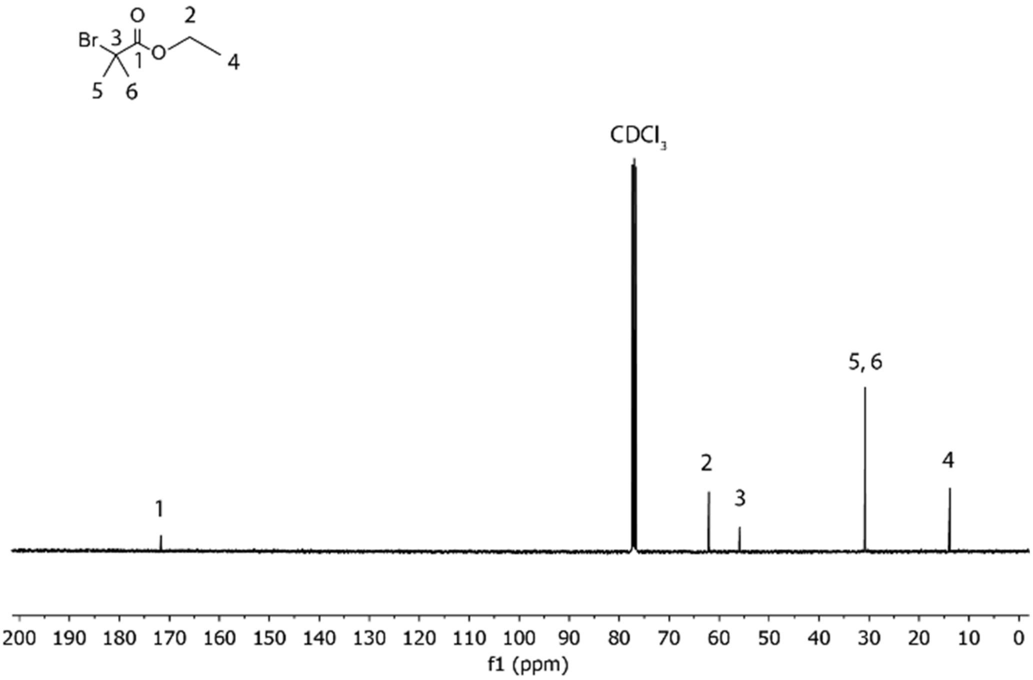

Figure S6. ^{13}C solution NMR spectrum of EtBiB in $CDCl_3$.

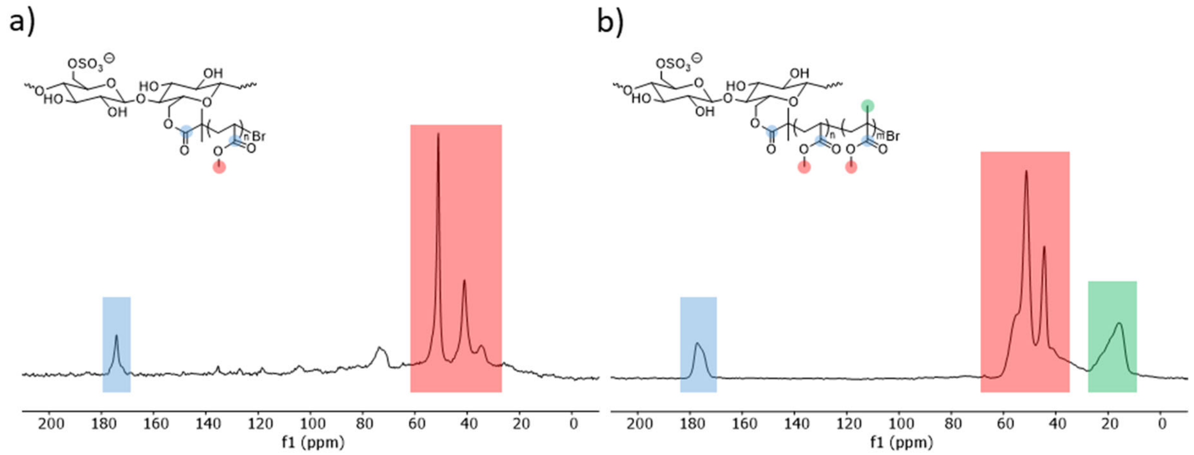

Figure S7. ^{13}C solid-state CP-MAS NMR spectra of a) CNC-*g*-PMA$_{15}$ and CNC-*g*-PMA$_5$-*b*-PMMA$_{12}$ recorded at a spinning rate of 60 kHz with 10 s relaxation time.

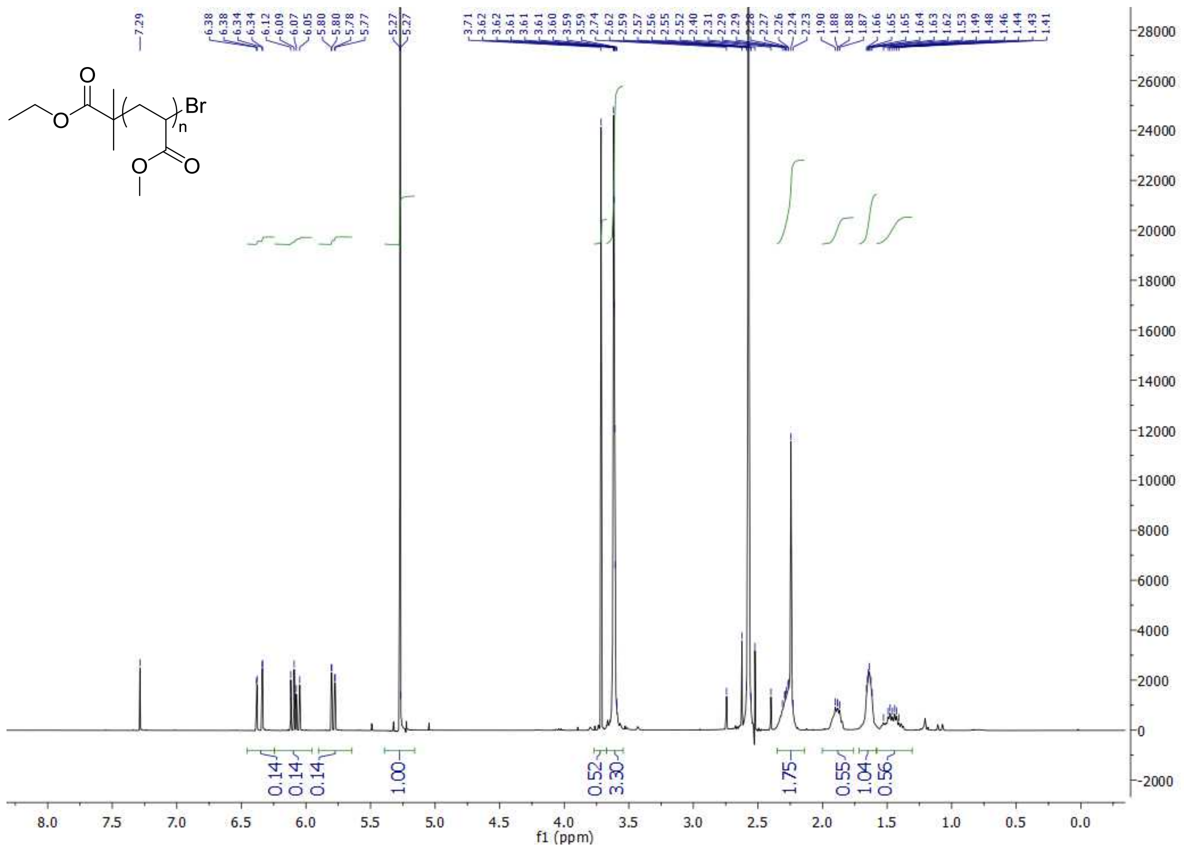

Figure S8. ^{1}H NMR spectrum of an aliquot taken from a PMA polymerization ([Monomer]:[Initiator]:[Cu^{II}]:[Ligand] of 200:1:0.05:0.25, a solvent:monomer ratio of 1:1 v/v) after 60 min. The spectrum was recorded in $CDCl_3$.

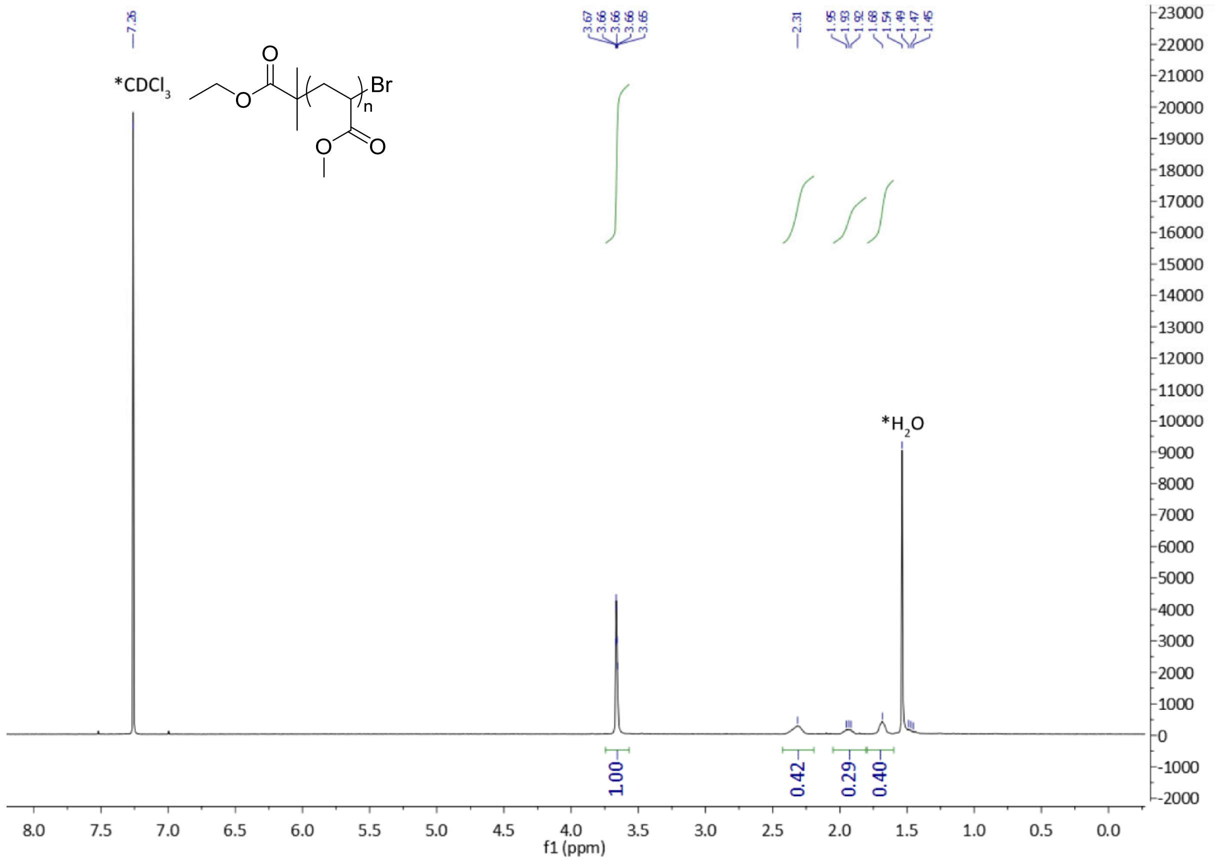

Figure S9. ^{1}H NMR spectrum of PMA_{15} in $CDCl_3$. The polymer was isolated after a reaction time of 60 min by precipitation into cold methanol and dried. The reaction conditions were [Monomer]:[Initiator]:[Cu^{II}]:[Ligand] of 200:1:0.05:0.25, a solvent:monomer ratio of 1:1 v/v.

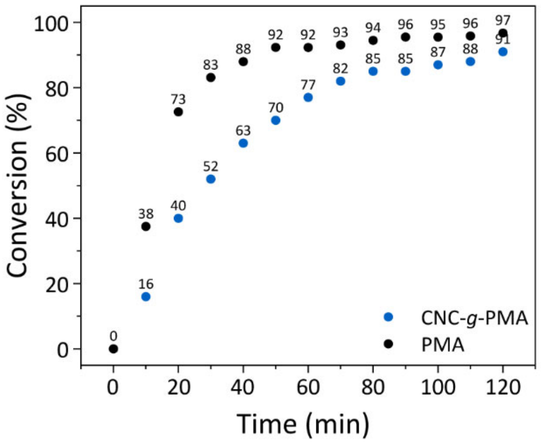

Figure S10. Monomer-to-polymer conversion versus time observed in reactions that afforded PMA and CNC-*g*-PMA, respectively. Aliquots were taken from the reaction mixtures in time intervals of 10 min. The conversion was calculated from the integrals of signals associated with the monomer's double bond at 6.36, 6.07, and 5.75 ppm and the polymer's methyl ester group at 3.66 ppm (cf. Figures S7 and Figure 3 in the manuscript for spectra recorded at 60 min time points) using Eq. S3.

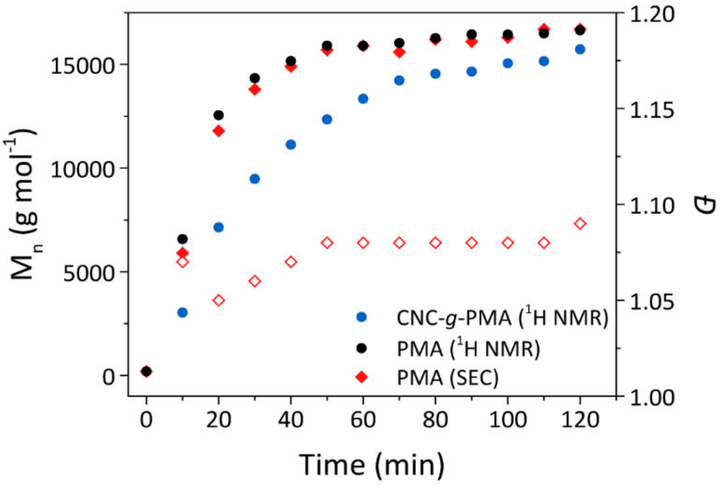

Figure S11. M_n (solid circles and diamonds) and dispersity (open diamonds) versus time observed in reactions that afforded PMA (black, red) and CNC-*g*-PMA (blue), respectively. Aliquots were taken from the reaction mixtures in time intervals of 10 min. The molecular weight data of PMA were established by SEC (red diamonds) and NMR (black circles) (Figure S11, Figure S9), while the molecular weight data of CNC-*g*-PMA are based on NMR (blue circles).

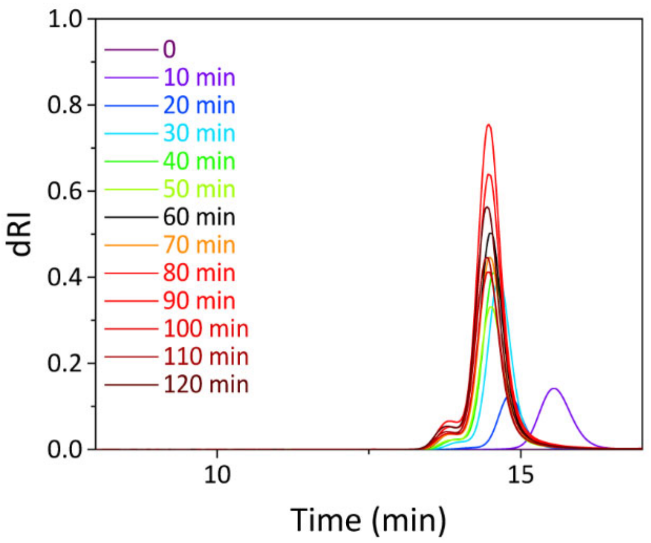

Figure S12. SEC traces of aliquots taken from the reaction mixtures to produce PMA in intervals of 10 min.

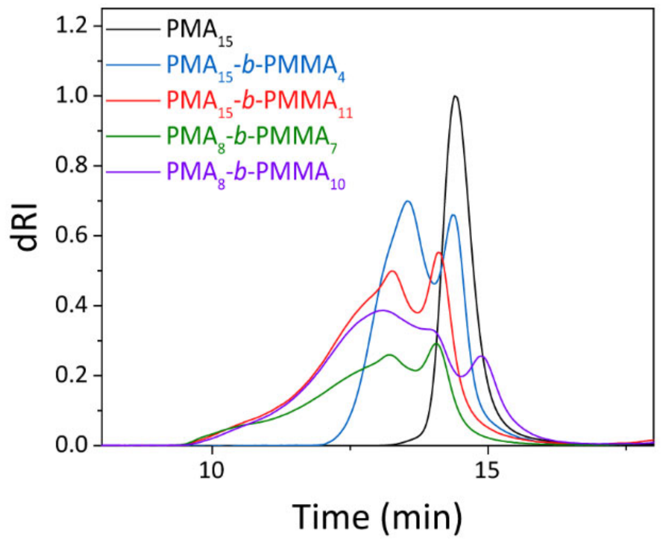

Figure S13. Size exclusion chromatograms of PMA and the series of PMA-*b*-PMMA diblock copolymers.

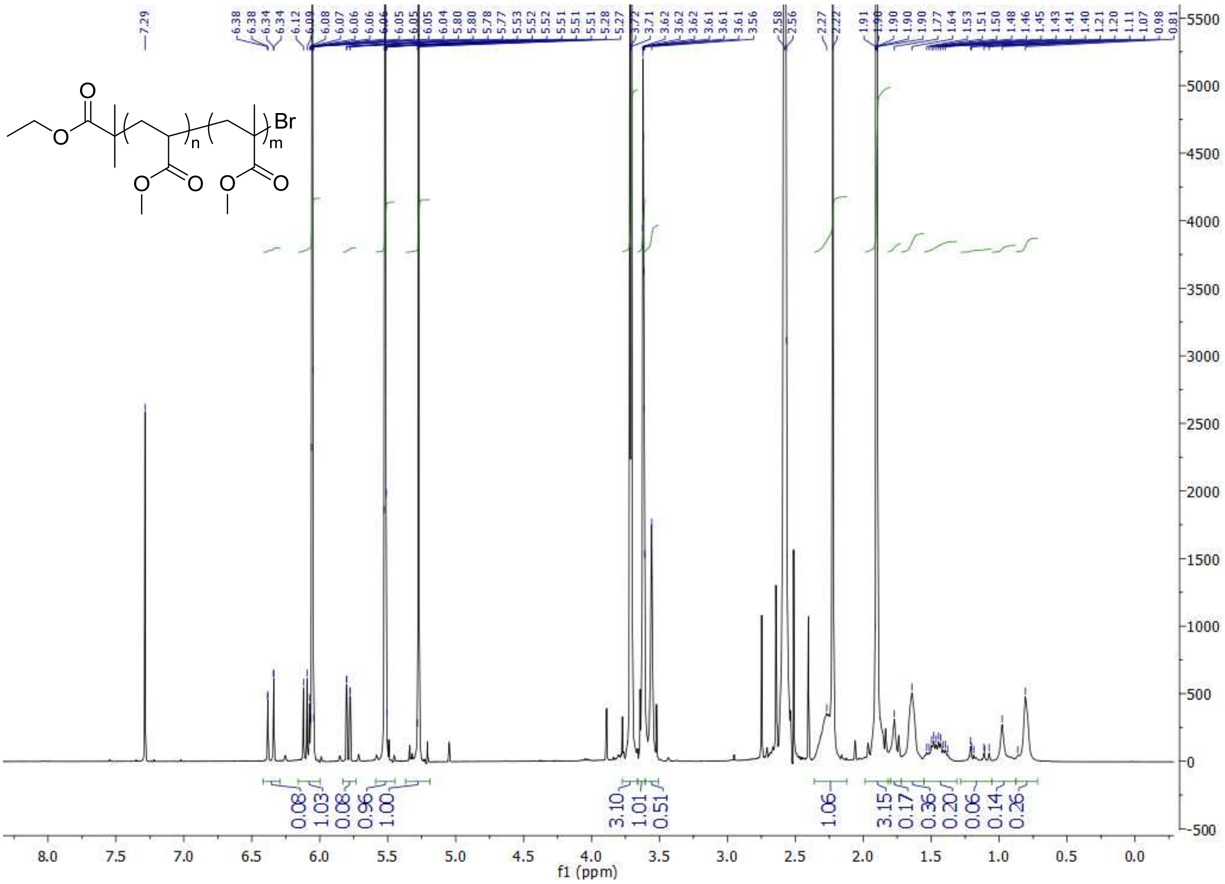

Figure S14. ^{1}H NMR spectrum of an aliquot taken from the reaction mixture to produce **PMA$_{15}$-*b*-PMMA$_4$** 60 min after the addition of MMA. The spectrum was recorded in $CDCl_3$. The reaction conditions were [MA]:[MMA]:[Initiator]:[Cu^{II}]:[Ligand] = 200:400:1:0.05:0.25, a solvent:monomer ratio of 1:1 v/v, and a reaction time of 1 h before the addition of MMA.

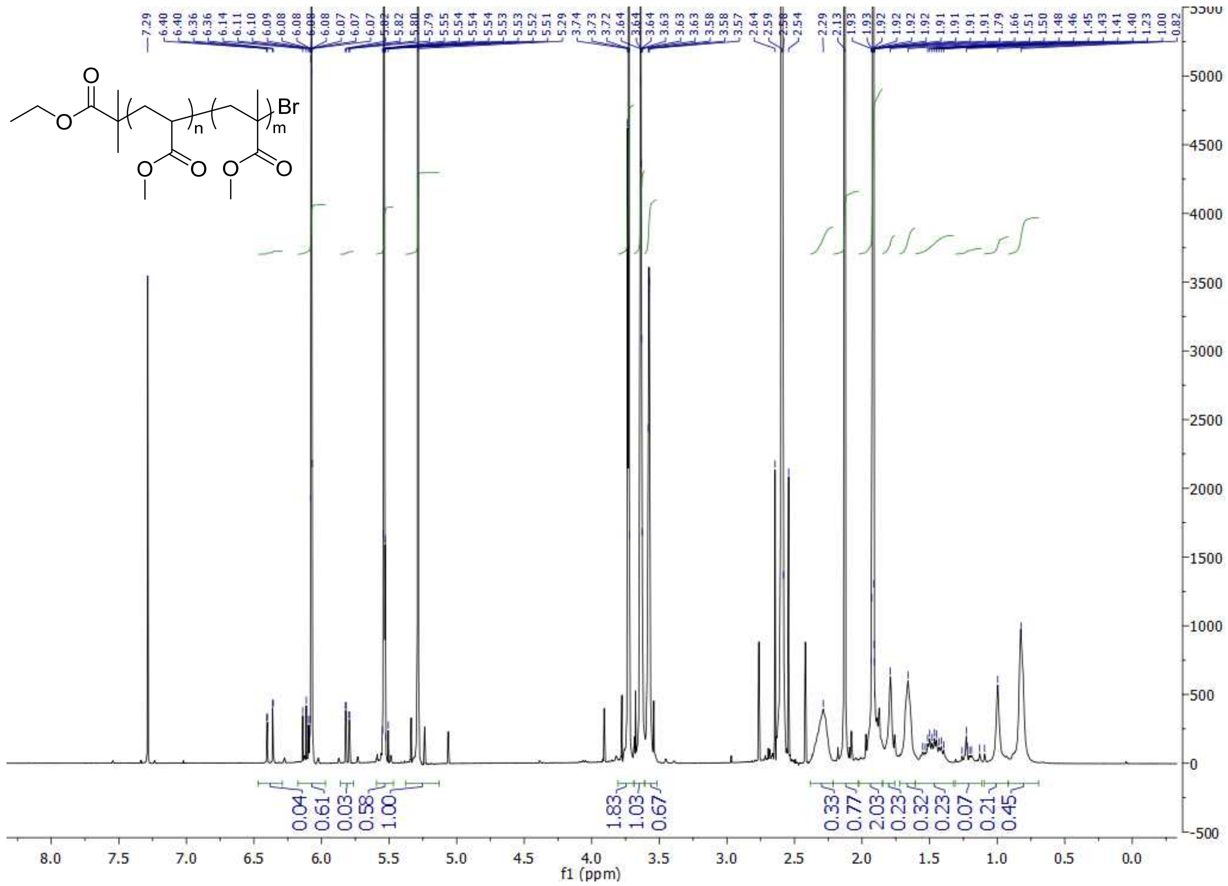

Figure S15. ^{1}H NMR spectrum of an aliquot taken from the reaction mixture to produce **PMA$_{15}$-*b*-PMMA$_{11}$** 120 min after the addition of MMA. The spectrum was recorded in $CDCl_3$. The reaction conditions were [MA]:[MMA]:[Initiator]:[Cu^{II}]:[Ligand] = 200:400:1:0.05:0.25, a solvent:monomer ratio of 1:1 v/v, and a reaction time of 1 h before the addition of MMA.

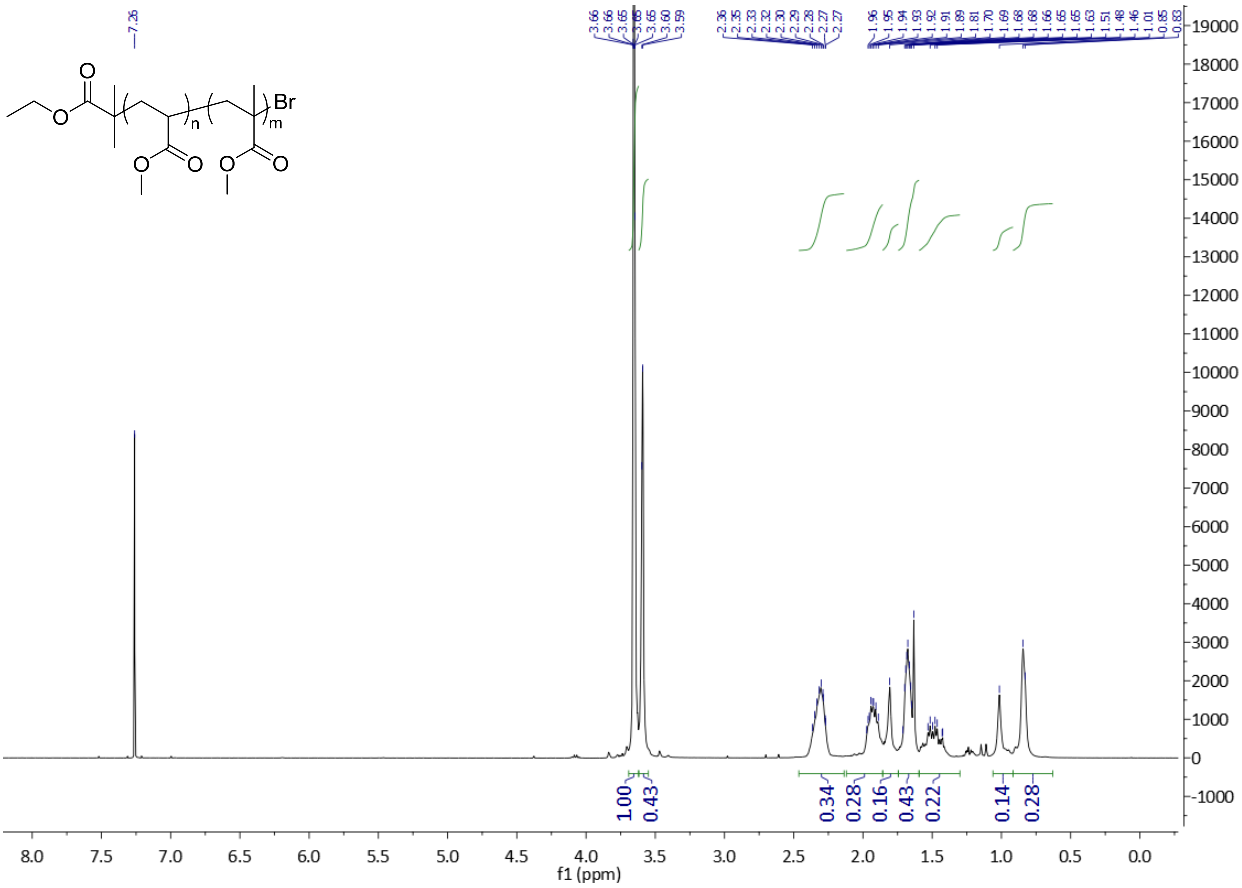

Figure S16. ^{1}H NMR spectrum of **PMA$_{15}$-*b*-PMMA$_4$** in $CDCl_3$. The polymer was isolated after a reaction time of 60 + 60 min by precipitation into cold methanol and dried. The reaction conditions were [MA]:[MMA]:[Initiator]:[Cu^{II}]:[Ligand] = 200:400:1:0.05:0.25, a solvent:monomer ratio of 1:1 v/v, and a reaction time of 1 h before the addition of MMA.

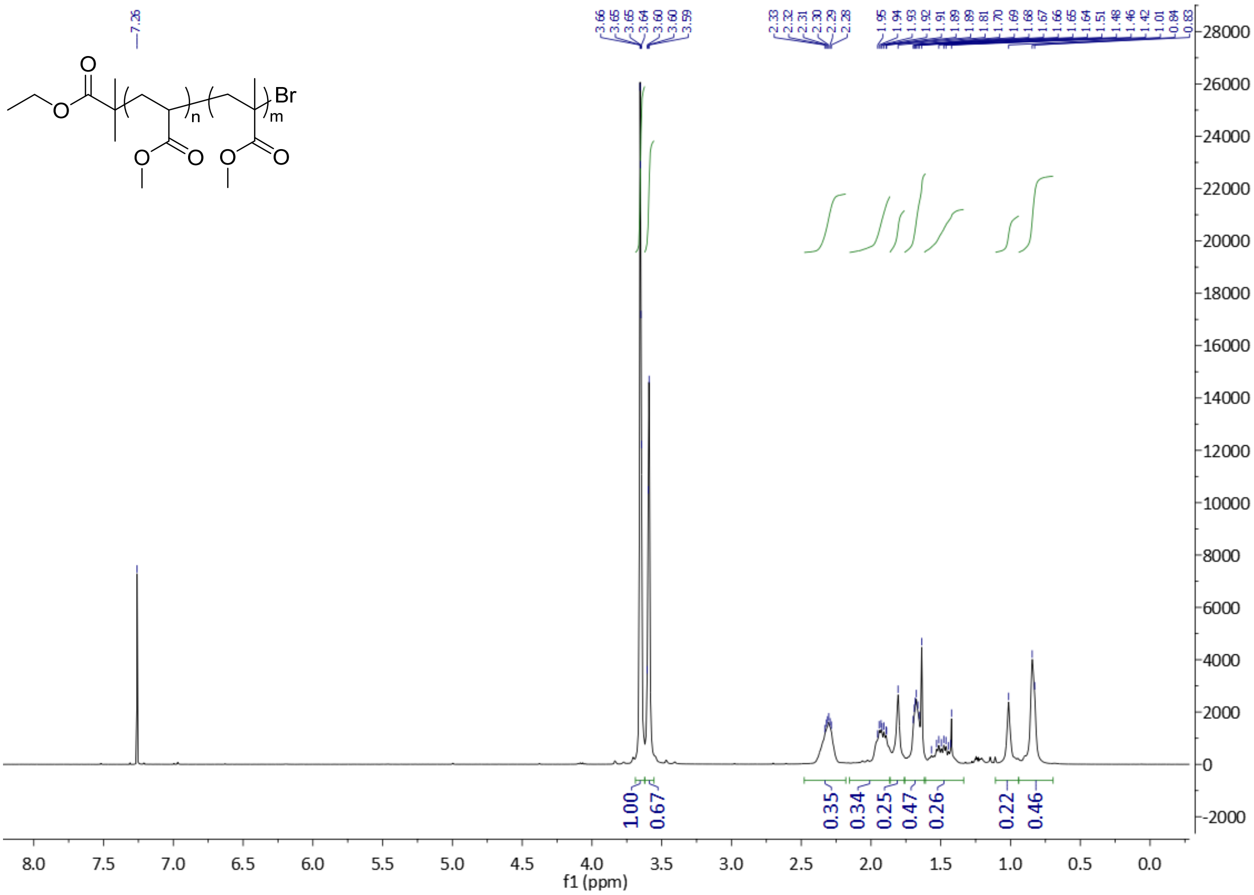

Figure S17. ^{1}H NMR spectrum of **PMA$_{15}$-*b*-PMMA$_{11}$** in $CDCl_3$. The polymer was isolated after a reaction time of 60 + 120 min by precipitation into cold methanol and dried. The reaction conditions were [MA]:[MMA]:[Initiator]:[Cu^{II}]:[Ligand] = 200:400:1:0.05:0.25, a solvent:monomer ratio of 1:1 v/v, and a reaction time of 1 h before the addition of MMA.

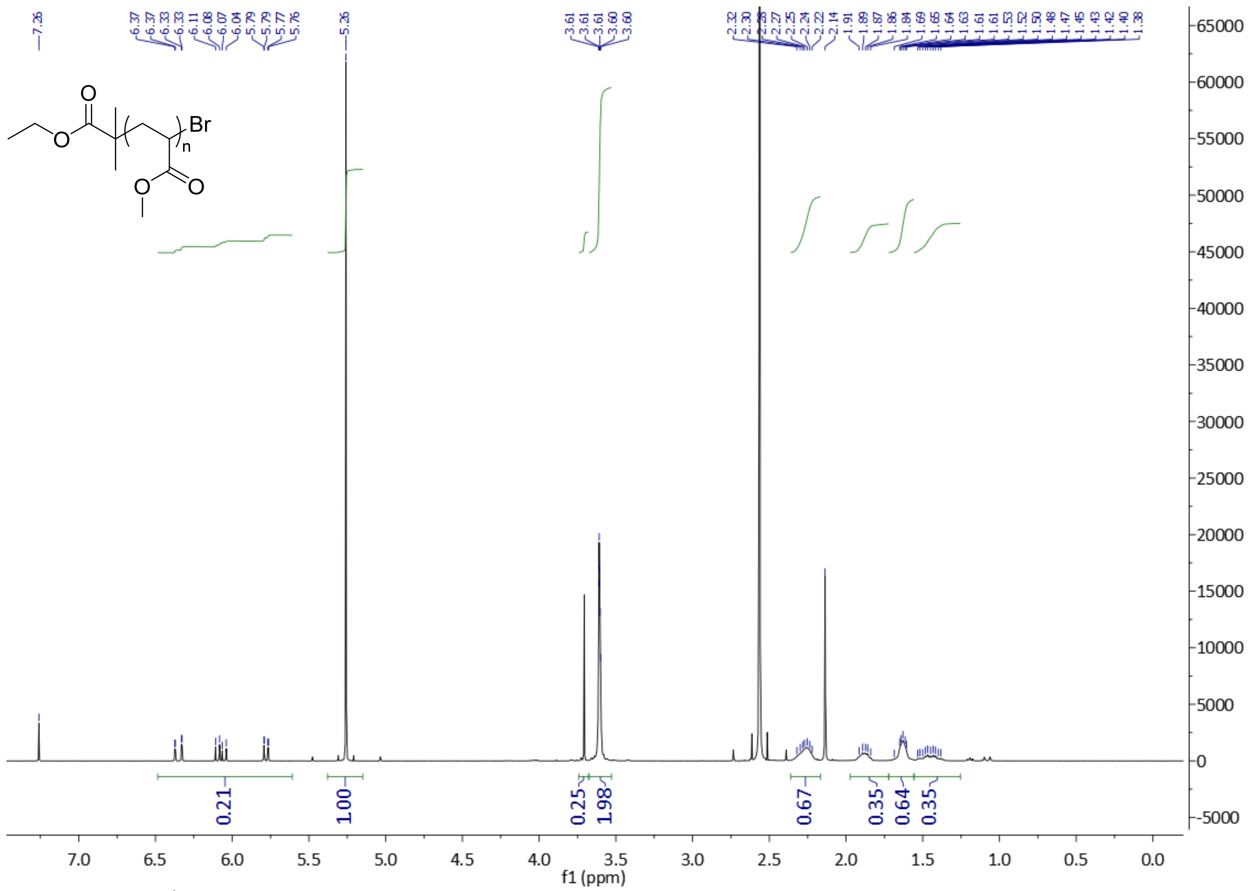

Figure S18. ^{1}H NMR spectrum of an aliquot taken from the reaction mixture to produce **PMA$_8$** after 60 min of reaction time. The spectrum was recorded in $CDCl_3$. The signal at 2.14 ppm is associated with an acetone impurity. The reaction conditions were [Monomer]:[Initiator]:[Cu^{II}]:[Ligand] of 100:1:0.05:0.25, a solvent:monomer ratio of 1:1 v/v.

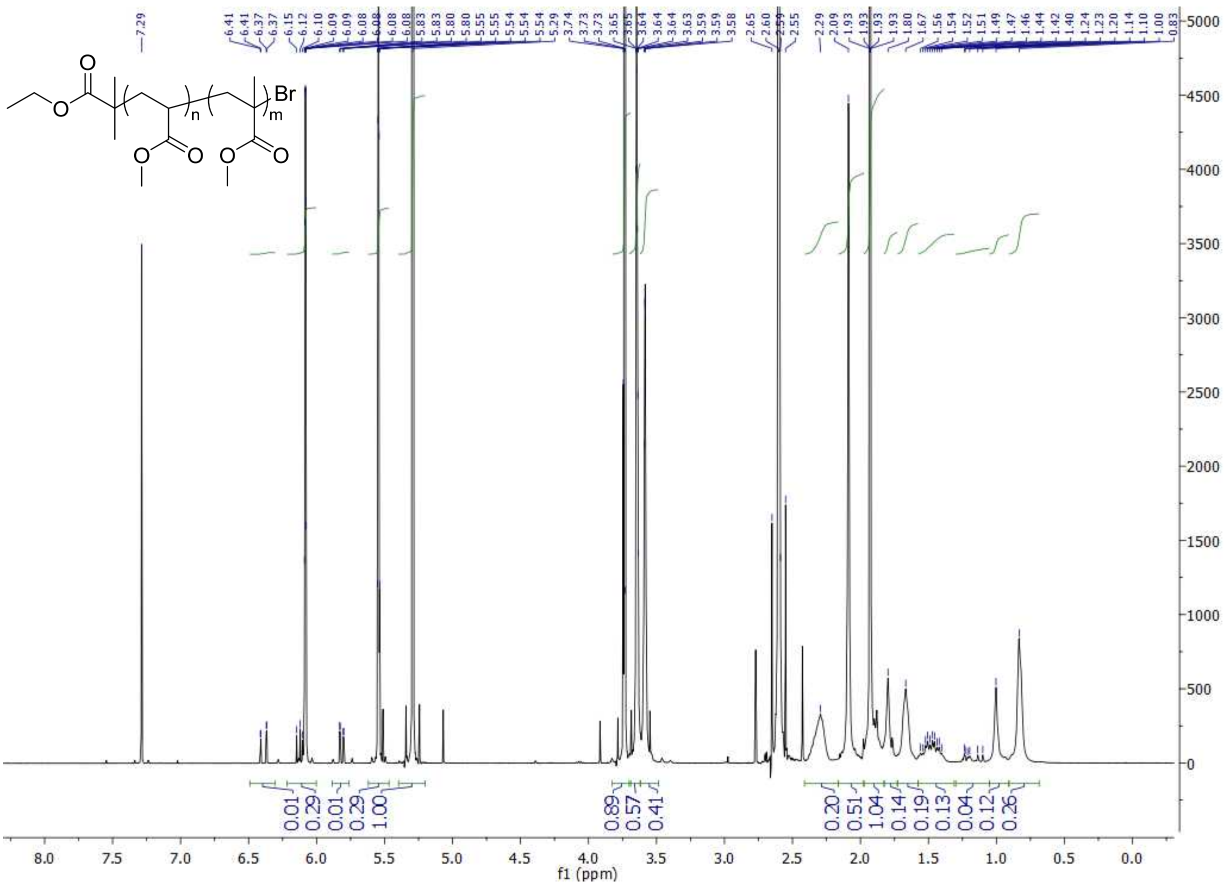

Figure S19. ^{1}H NMR spectrum of an aliquot taken from the reaction mixture to produce **PMA$_8$-*b*-PMMA$_7$** 60 min after the addition of MMA. The spectrum was recorded in $CDCl_3$. The reaction conditions were [MA]:[MMA]:[Initiator]:[Cu^{II}]:[Ligand] = 100:400:1:0.05:0.25, a solvent:monomer ratio of 1:1 v/v, and a reaction time of 1 h before the addition of MMA.

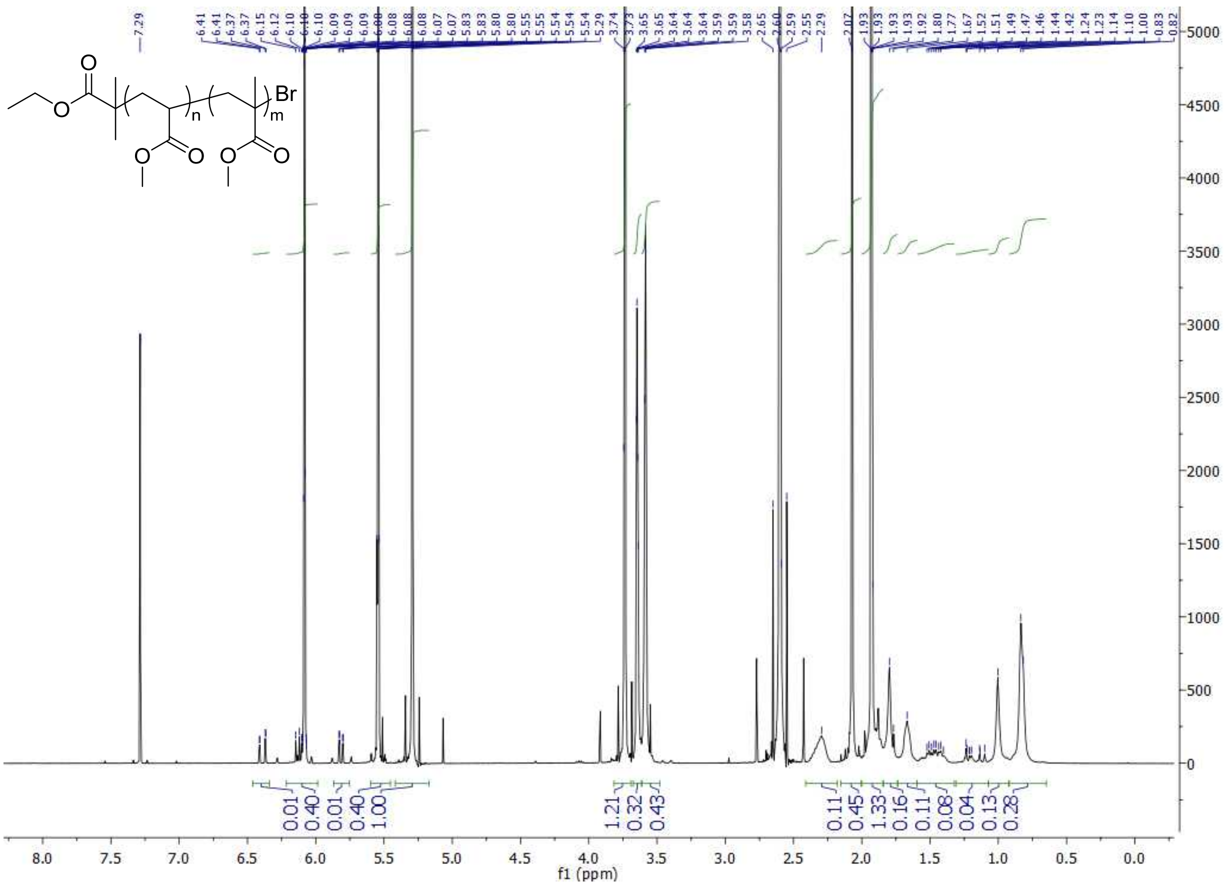

Figure S20. ^{1}H NMR spectrum of an aliquot taken from the reaction mixture to produce **PMA$_8$-*b*-PMMA$_{10}$** 120 min after the addition of MMA. The spectrum was recorded in $CDCl_3$. The reaction conditions were [MA]:[MMA]:[Initiator]:[Cu^{II}]:[Ligand] = 100:400:1:0.05:0.25, a solvent:monomer ratio of 1:1 v/v, and a reaction time of 1 h before the addition of MMA.

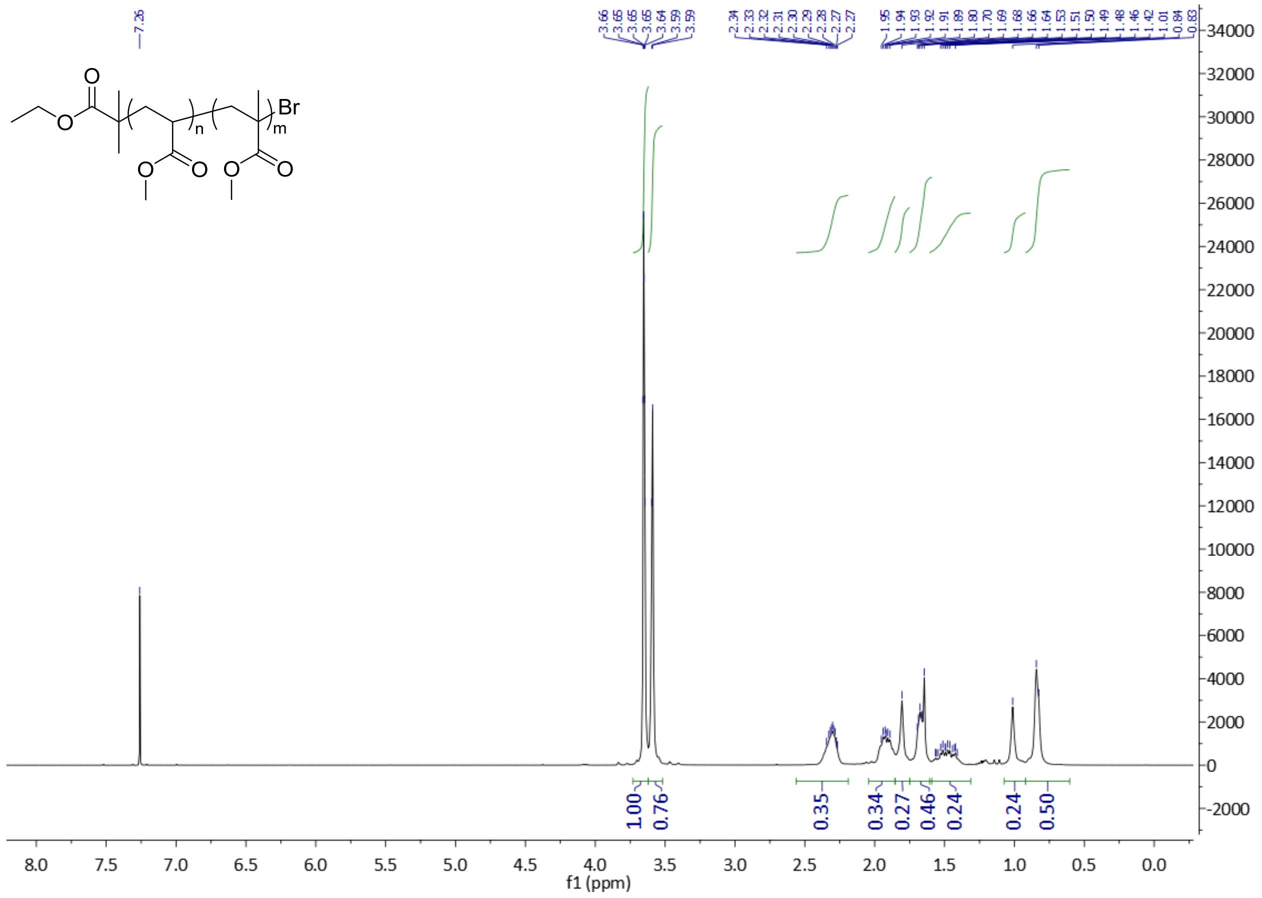

Figure S21. ^{1}H NMR spectrum of **PMA$_8$-*b*-PMMA$_7$** in $CDCl_3$. The polymer was isolated after a reaction time of 60 + 60 min by precipitation into cold methanol and dried. The reaction conditions were [MA]:[MMA]:[Initiator]:[Cu^{II}]:[Ligand] = 100:400:1:0.05:0.25, a solvent:monomer ratio of 1:1 v/v, and a reaction time of 1 h before the addition of MMA.

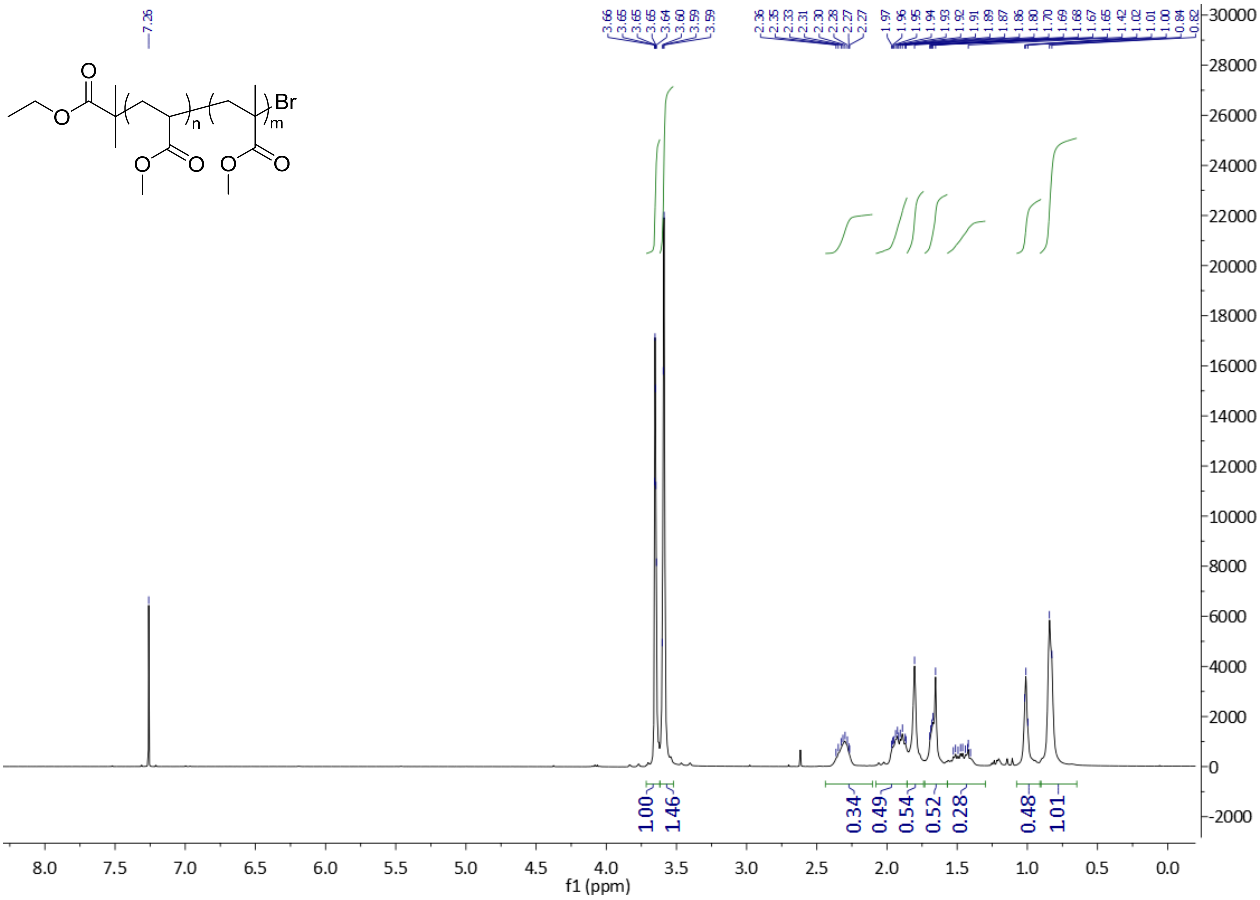

Figure S22. ^{1}H NMR spectrum of **PMA$_8$-*b*-PMMA$_{10}$** in $CDCl_3$. The polymer was isolated after a reaction time of 60 + 120 min by precipitation into cold methanol and dried. The reaction conditions were [MA]:[MMA]:[Initiator]:[Cu^{II}]:[Ligand] = 100:400:1:0.05:0.25, a solvent:monomer ratio of 1:1 v/v, and a reaction time of 1 h before the addition of MMA.

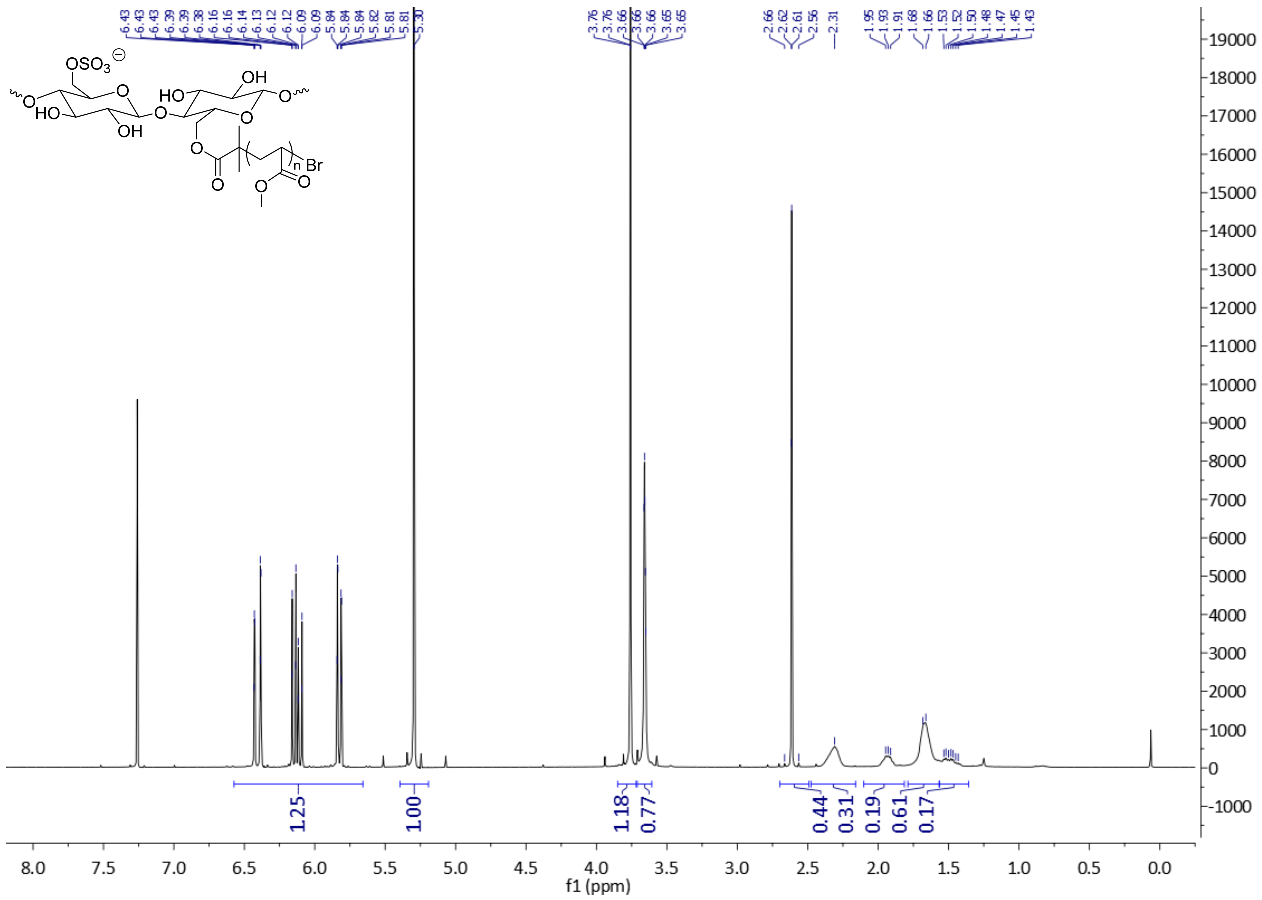

Figure S23. ^{1}H NMR spectrum of an aliquot taken from the reaction mixture to produce **CNC-*g*-PMA$_{20}$** ([MA]:[Initiator]:[Cu^{II}]:[Ligand] = 500:1:0.05:0.25, solvent:monomer ratio of 1:1 v/v) after 30 min. The spectrum was recorded in $CDCl_3$.

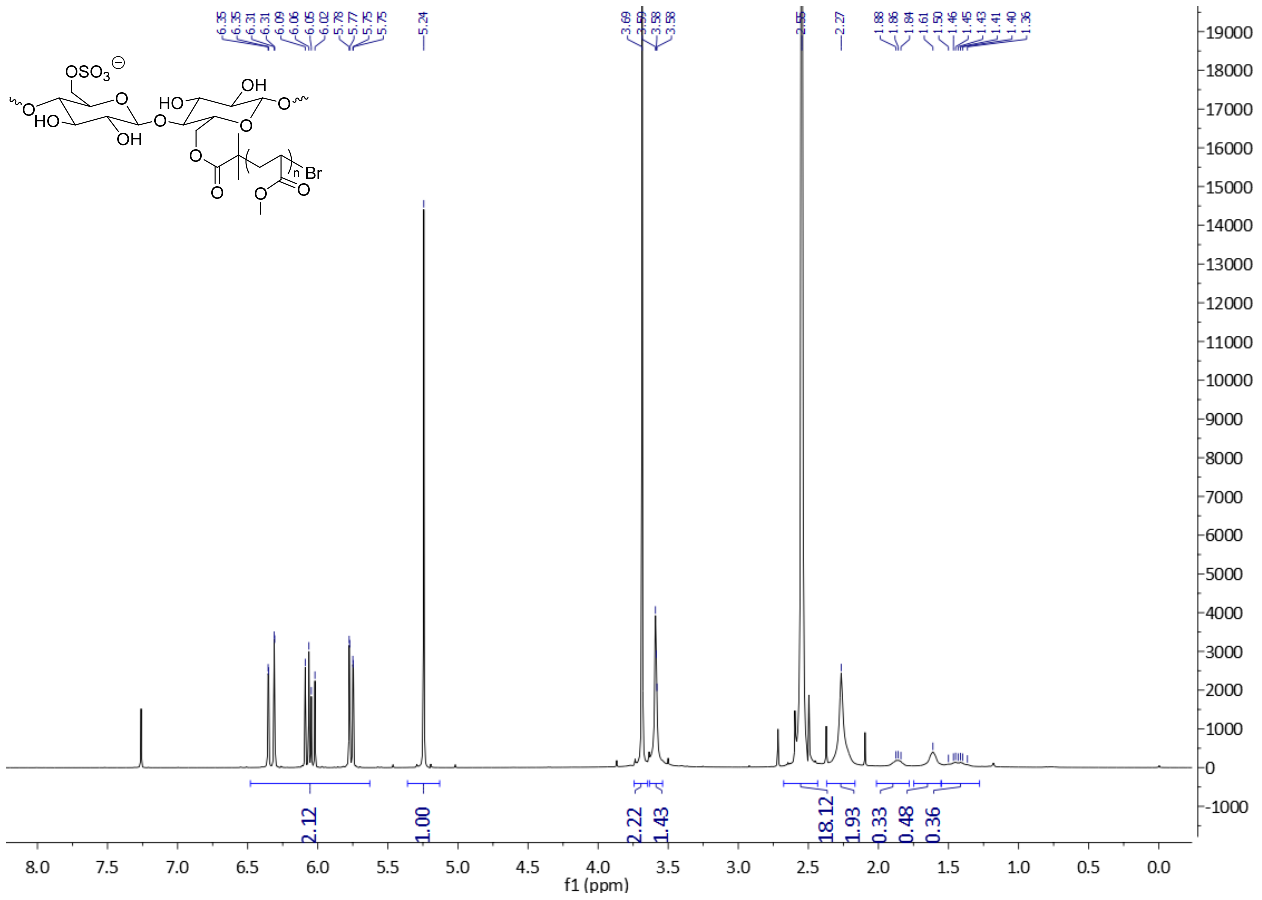

Figure S24. ^{1}H NMR spectrum of an aliquot taken from the reaction mixture to produce **CNC-*g*-PMA$_{35}$** ([MA]:[Initiator]:[Cu^{II}]:[Ligand] = 1000:1:0.05:0.25, solvent:monomer ratio of 1:1 v/v) after 30 min. The spectrum was recorded in $CDCl_3$.

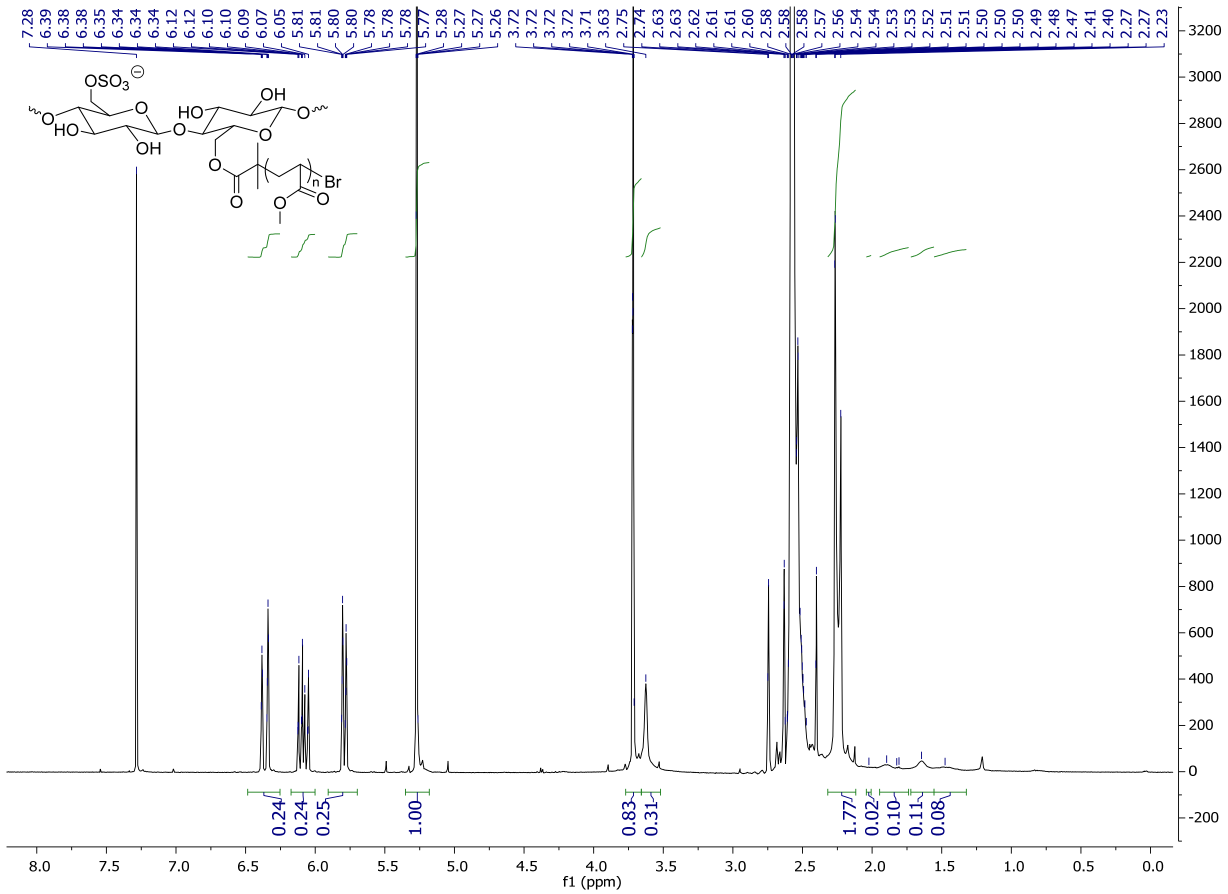

Figure S25. ^{1}H NMR spectrum of an aliquot taken from the reaction mixture to produce **CNC-*g*-PMA$_5$** ([MA]:[Initiator]:[Cu^{II}]:[Ligand] = 100:1:0.05:0.25, solvent:monomer ratio of 1:1 v/v) after 60 min. The spectrum was recorded in $CDCl_3$.

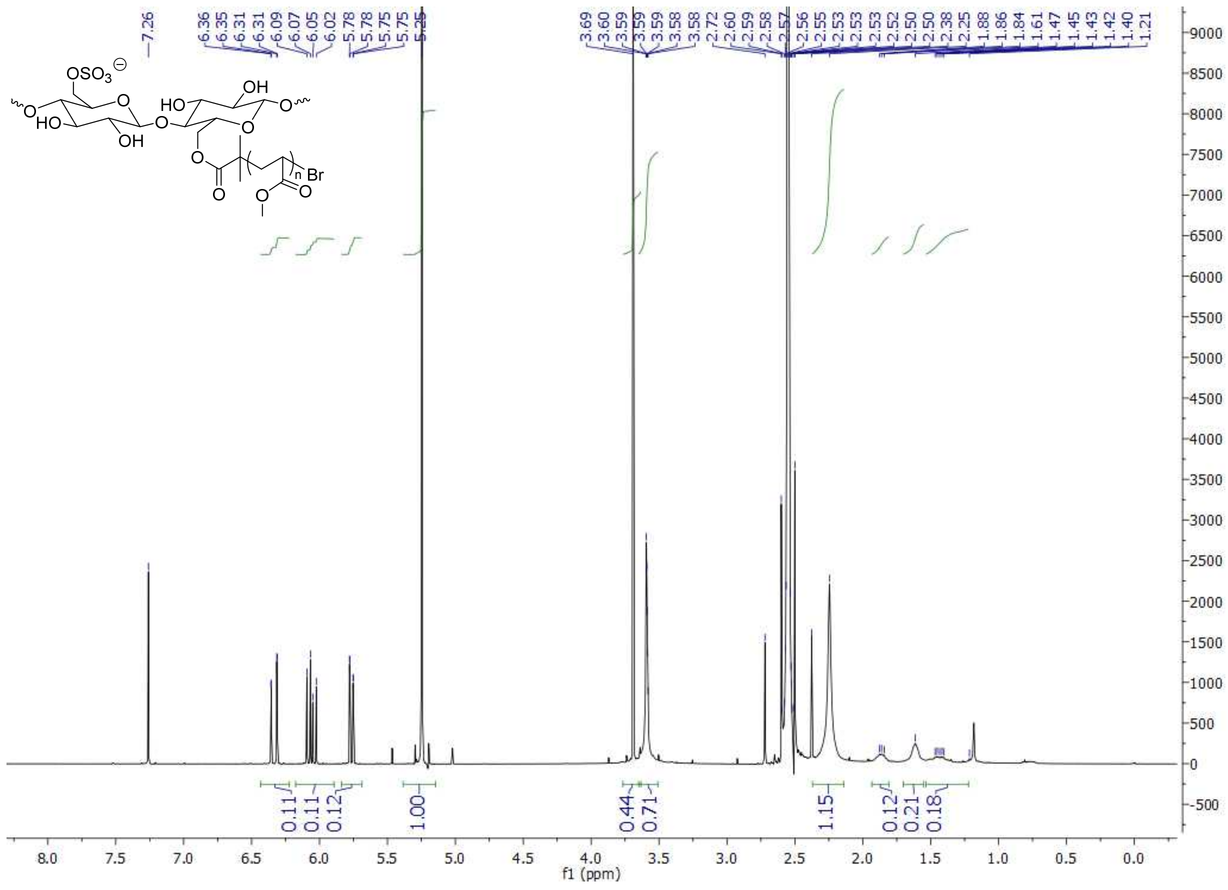

Figure S26. ^{1}H NMR spectrum of an aliquot taken from the reaction mixture to produce **CNC-*g*-PMA$_8$** ([MA]:[Initiator]:[Cu^{II}]:[Ligand] = 100:1:0.05:0.25, solvent:monomer ratio of 1:1 v/v) after 60 min. The spectrum was recorded in $CDCl_3$.

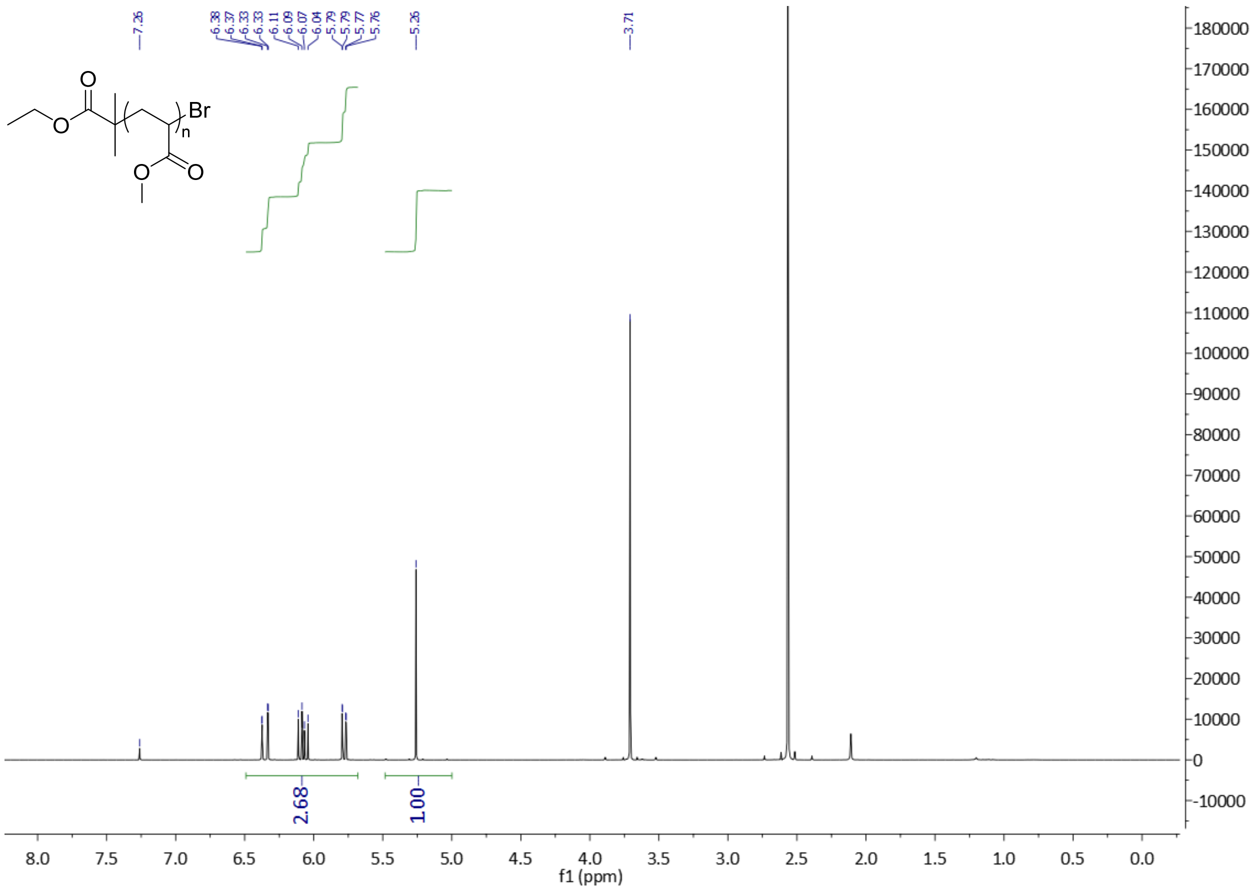

Figure S27. ^{1}H NMR spectrum of an aliquot taken from the reaction mixture to produce **PMA$_{15}$** after a reaction time of 0 min and measured in $CDCl_3$. The signal at 5.26 ppm is associated with the 5 μL of dichloromethane that was added as an internal standard. The signal at 2.14 ppm is associated with an acetone impurity. The reaction conditions were [Monomer]:[Initiator]:[Cu^{II}]:[Ligand] of 200:1:0.05:0.25, a solvent:monomer ratio of 1:4 v/v.

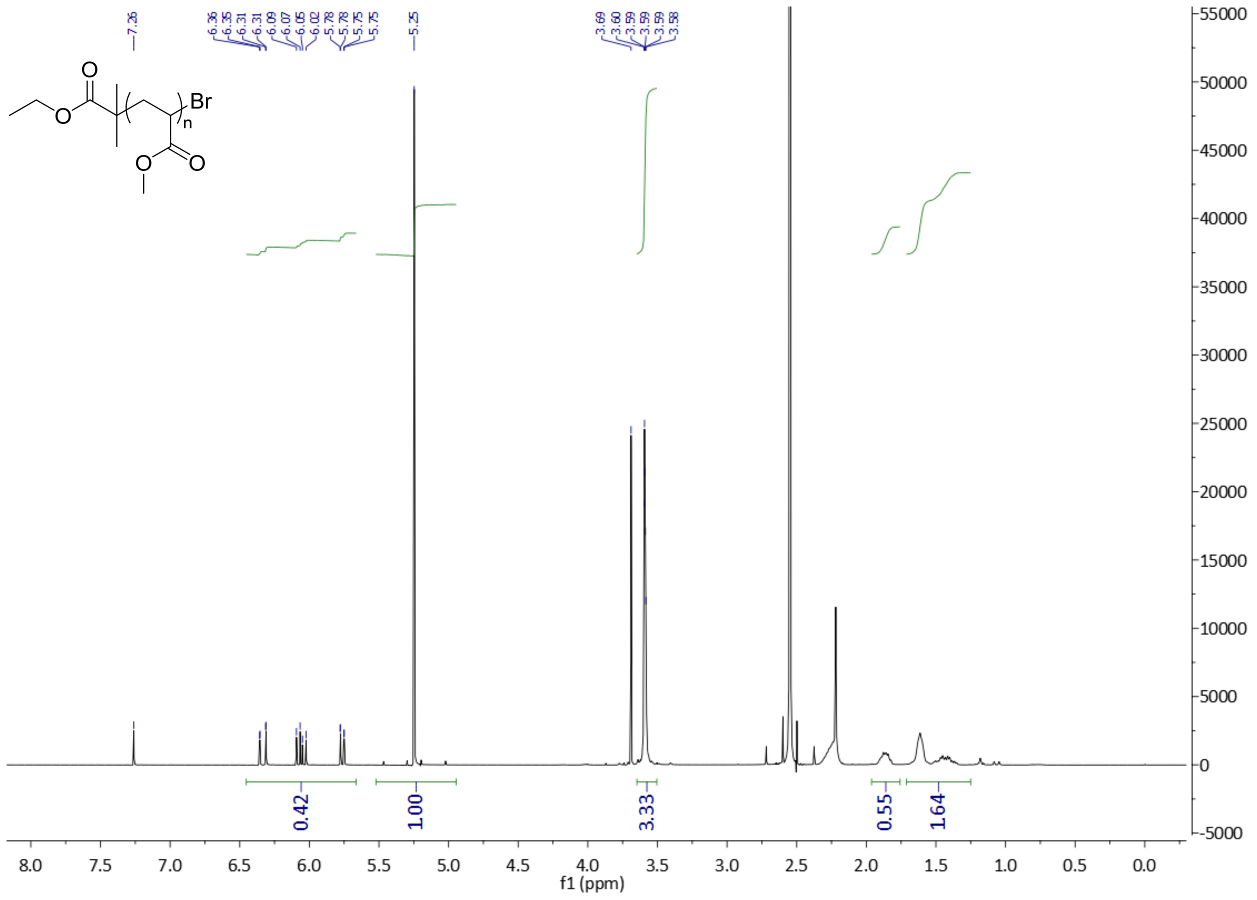

Figure S28. ^{1}H NMR spectrum of an aliquot **PMA$_{15}$** taken @ 1h and measured in $CDCl_3$. The signal at 5.25 ppm is associated with the 5 μL of dichloromethane that was added as an internal standard. The signal at 2.14 ppm is associated with an acetone impurity. The reaction conditions were [Monomer]:[Initiator]:[Cu^{II}]:[Ligand] of 200:1:0.05:0.25, a solvent:monomer ratio of 1:4 v/v.

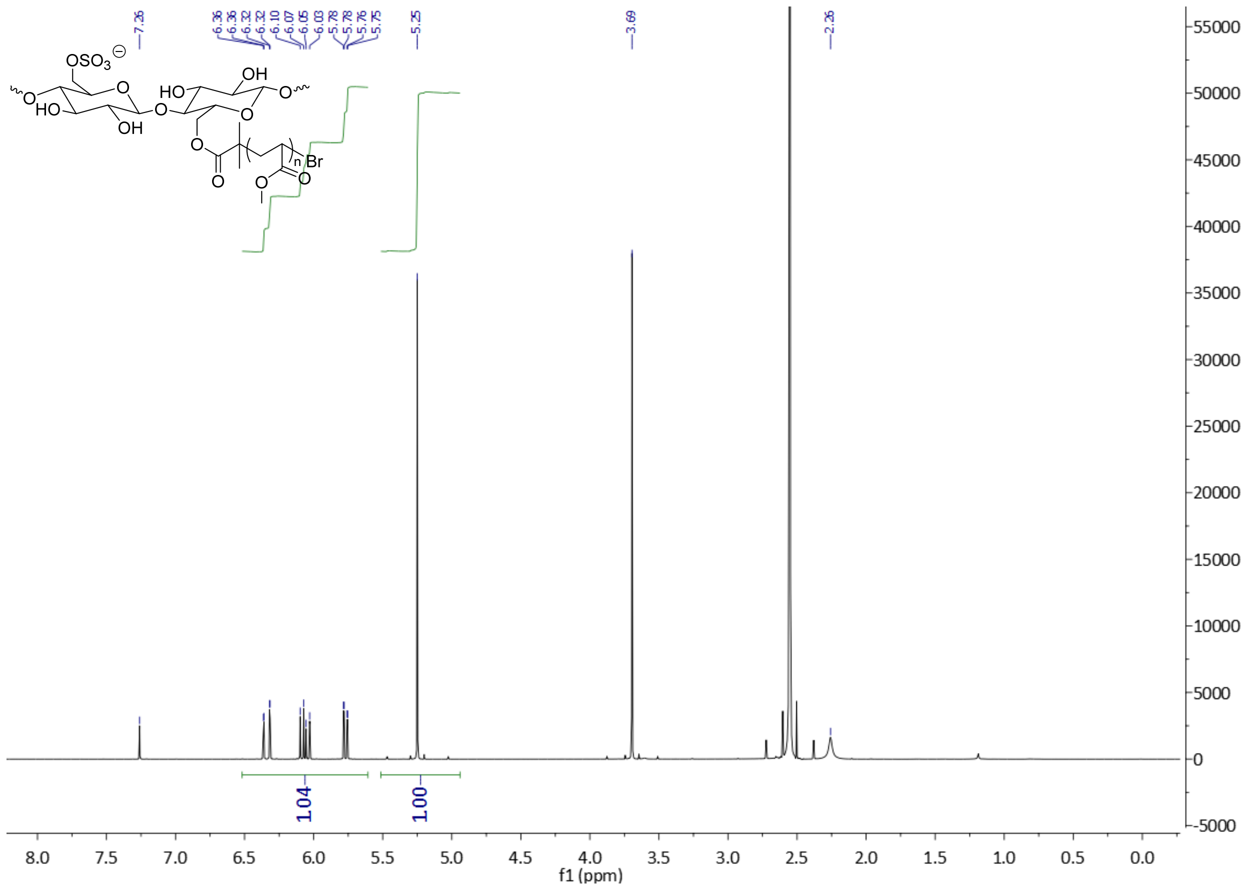

Figure S29. ^{1}H NMR spectrum of an aliquot of **CNC-*g*-PMA$_{15}$** taken @ t_0 and measured in $CDCl_3$. The signal at 5.25 ppm is associated with the 5 µL of dichloromethane that was added as an internal standard. The signal at 2.26 ppm is associated with an acetone impurity. The reaction conditions were [Monomer]:[Initiator]:[Cu^{II}]:[Ligand] of 200:1:0.05:0.25, a solvent:monomer ratio of 1:4 v/v.

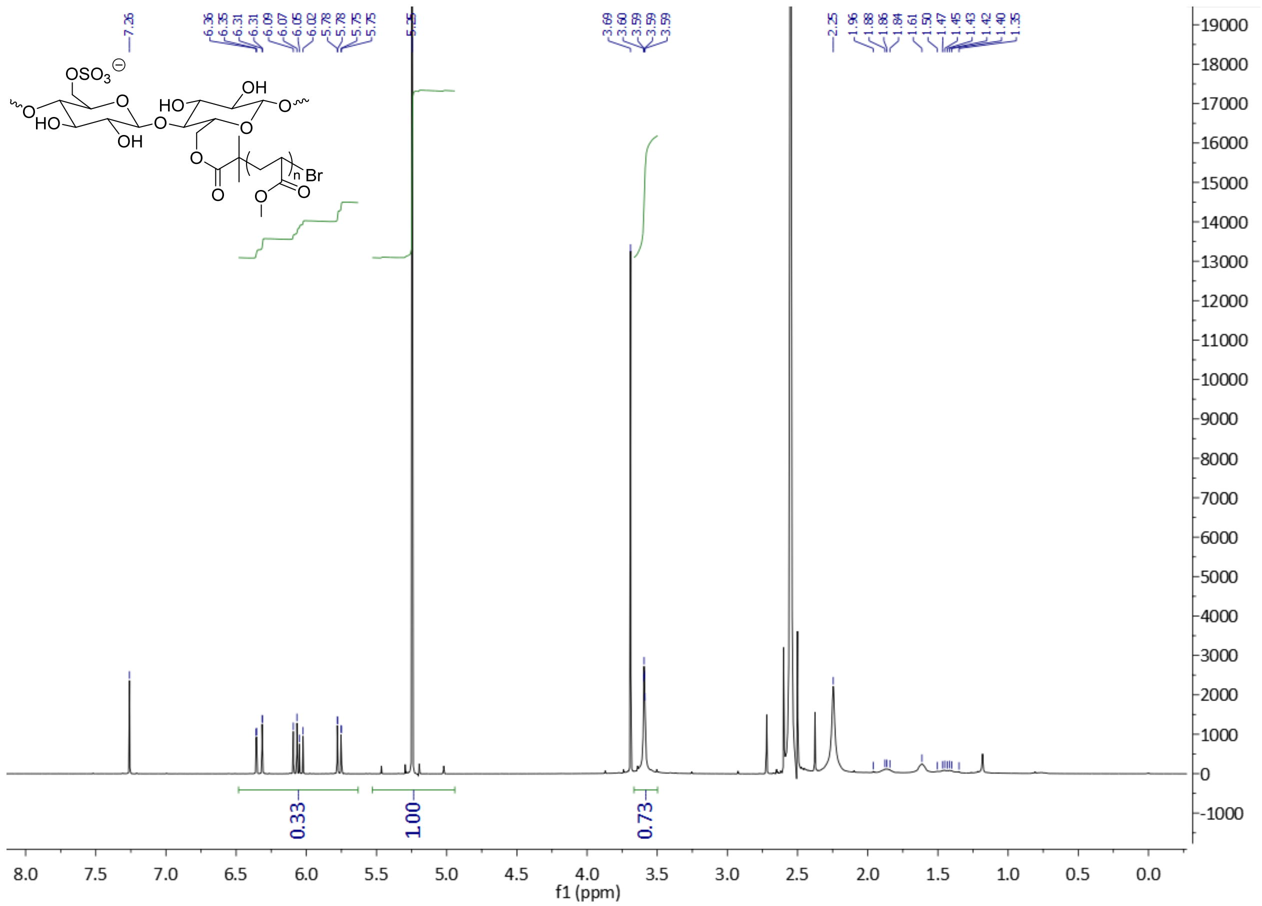

Figure S30. ^{1}H NMR spectrum of an aliquot of **CNC-*g*-PMA$_{15}$** taken @ 1h and measured in $CDCl_3$. The signal at 5.25 ppm is associated with the 5 µL of dichloromethane that was added as an internal standard. The signal at 2.25 ppm is associated with an acetone impurity. The reaction conditions were [Monomer]:[Initiator]:[Cu^{II}]:[Ligand] of 200:1:0.05:0.25, a solvent:monomer ratio of 1:4 v/v.

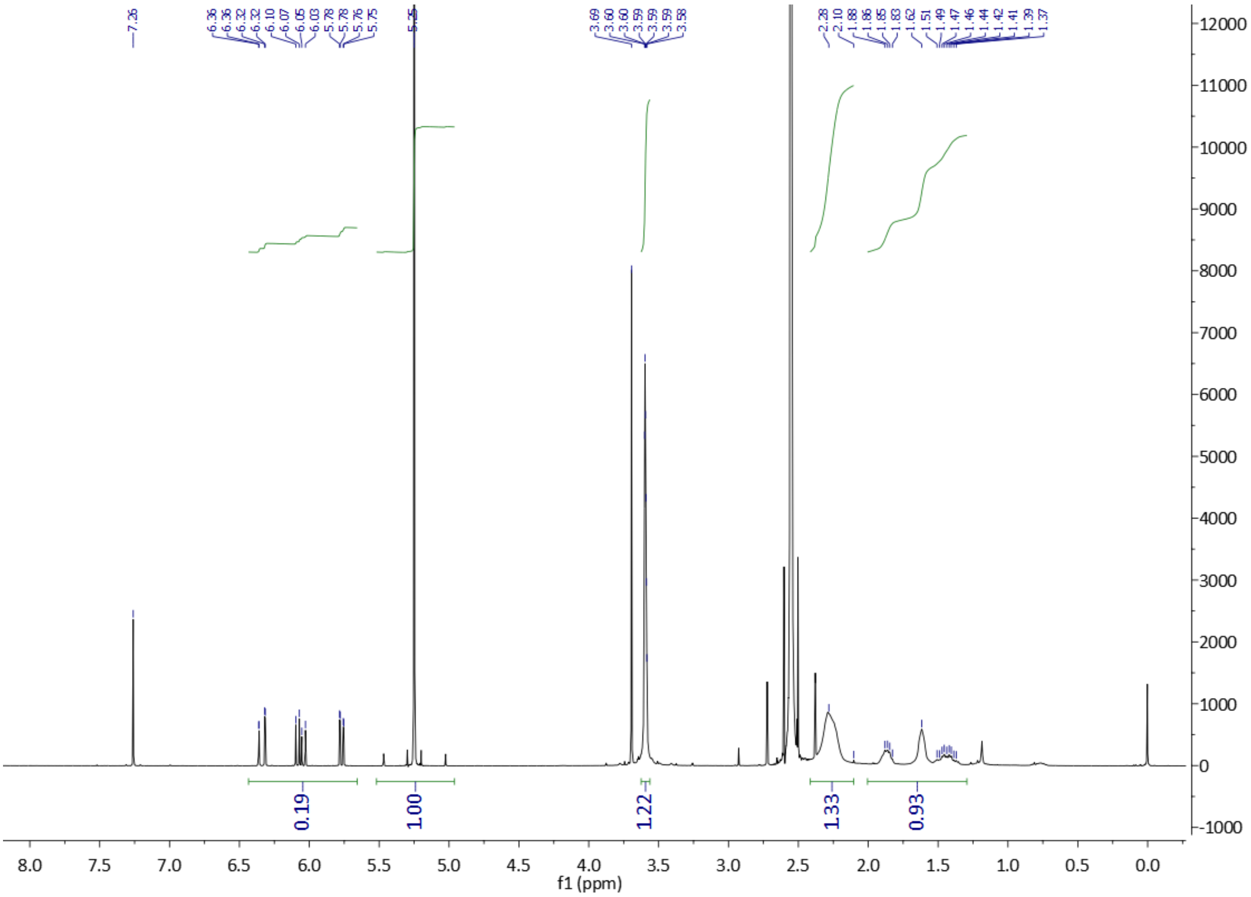

Figure S31. ^{1}H NMR spectrum of an aliquot of **CNC-*g*-PMA$_{15}$** taken @ 2h and measured in $CDCl_3$. The signal at 5.25 ppm is associated with the 5 µL of dichloromethane that was added as an internal standard. The signal at 2.25 ppm is associated with an acetone impurity. The reaction conditions were [Monomer]:[Initiator]:[Cu^{II}]:[Ligand] of 200:1:0.05:0.25, a solvent:monomer ratio of 1:4 v/v.

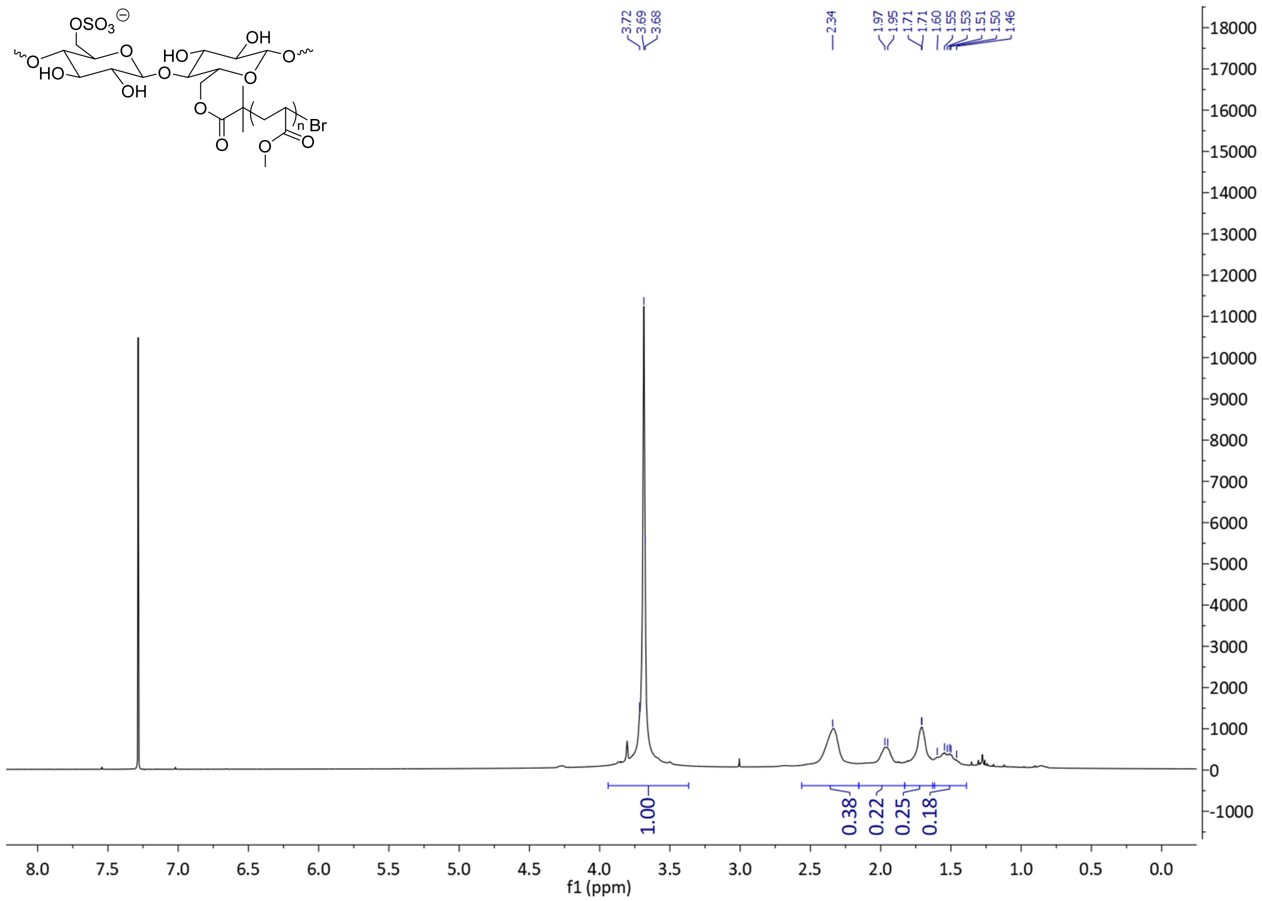

Figure S32. ^{1}H NMR spectrum of **CNC-*g*-PMA$_5$** (isolated after a reaction time of 60 min and purified by dialysis against THF) that was redispersed in $CDCl_3$.

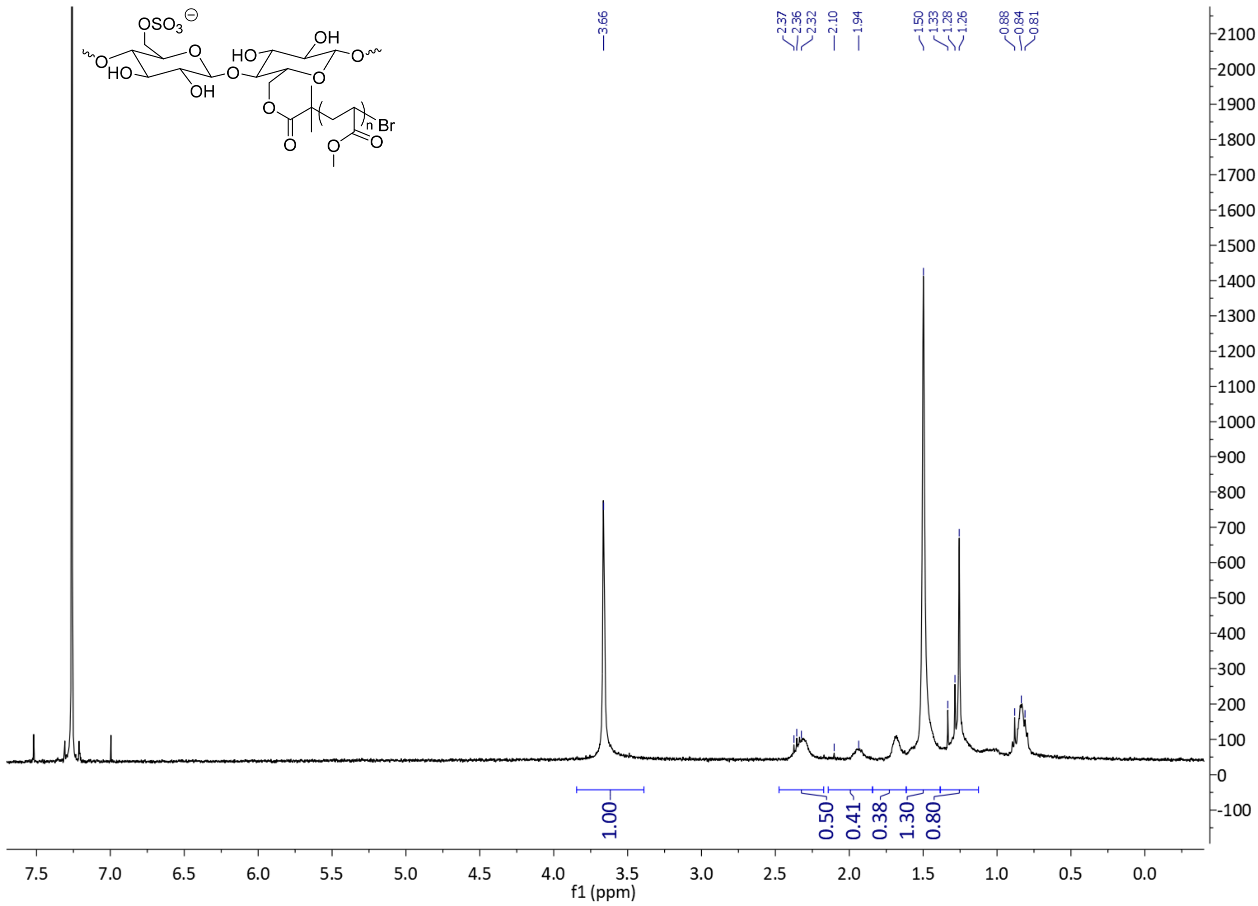

Figure S33. ^{1}H NMR spectrum of **CNC-*g*-PMA$_8$** (isolated after a reaction time of 120 min and purified by dialysis against THF) that was redispersed in $CDCl_3$.

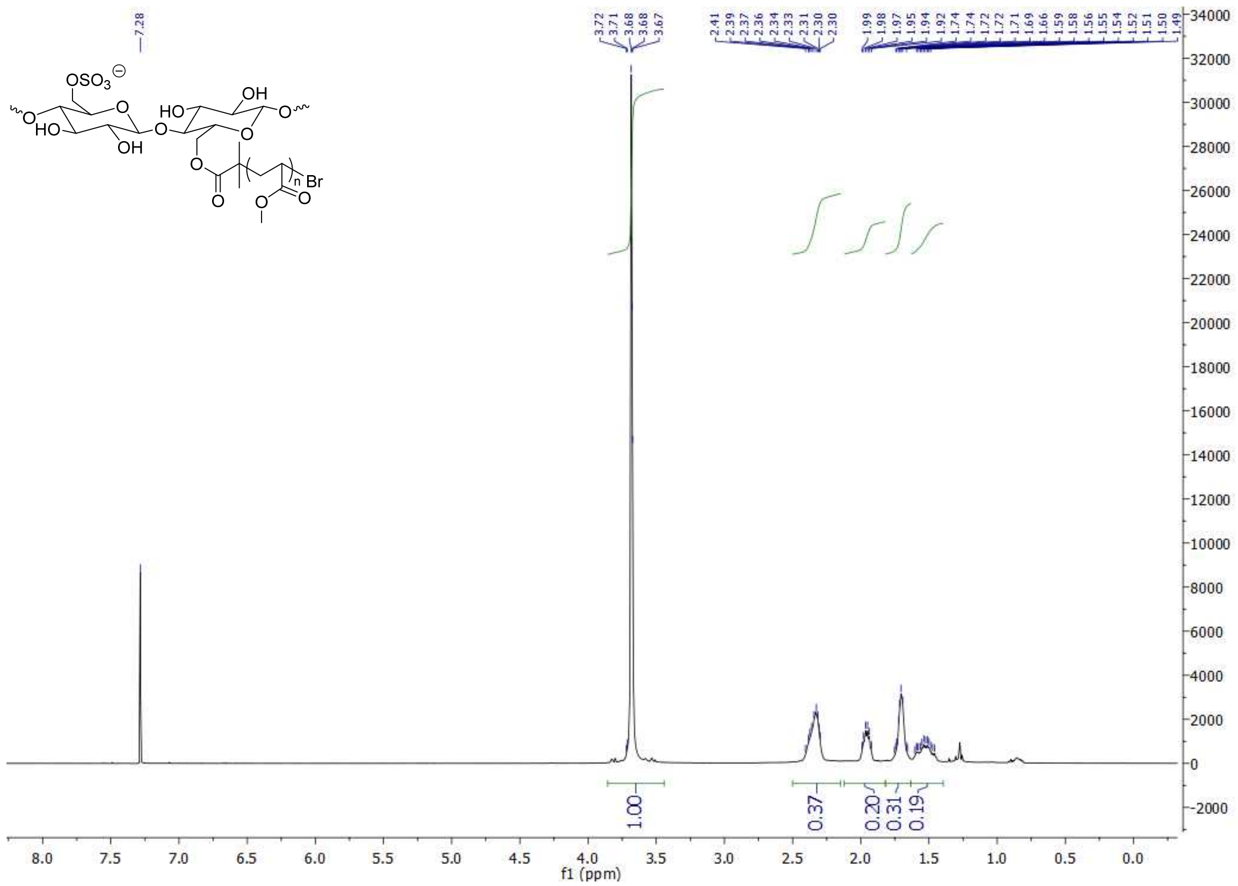

Figure S34. ^{1}H NMR spectrum of **CNC-*g*-PMA$_{15}$** (isolated after a reaction time of 120 min and purified by dialysis against THF) that was redispersed in $CDCl_3$.

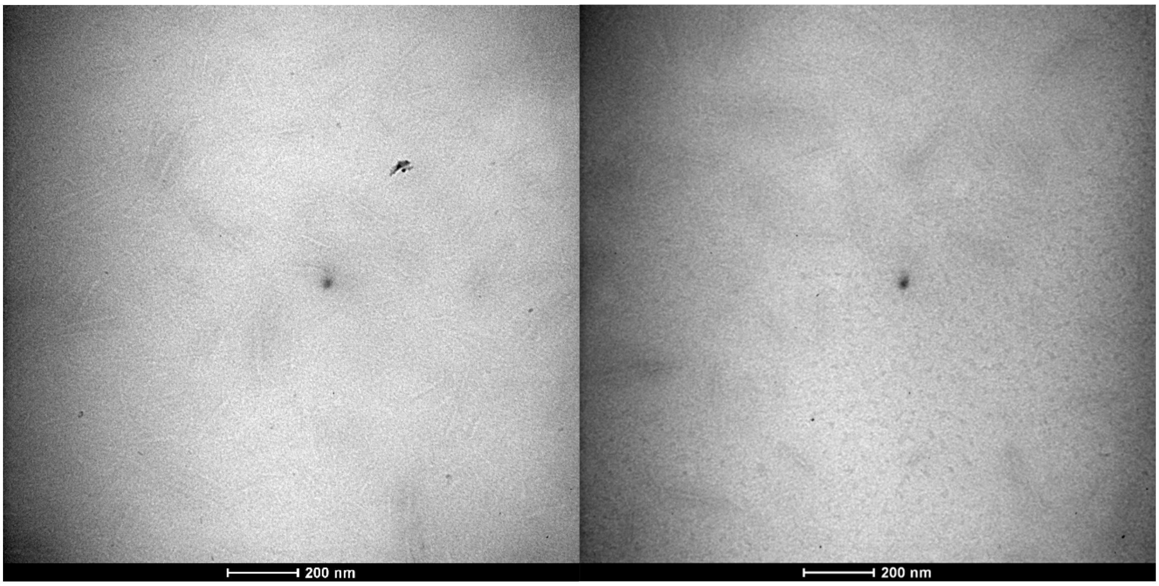

Figure S35. TEM micrographs of one representative spot of a spin-coated 0.1 wt% THF dispersion of **CNC-g-PMA$_8$** (left), showcasing an average length of 185 ± 31 nm and an average width of 31.8 ± 7 nm and of spin-coated 0. 1 wt% THF dispersion of **CNC-g-PMA$_8$-b-PMMA$_7$** (right), showcasing an average length of 188 ± 21 nm and an average width of 33.5 ± 8 nm.

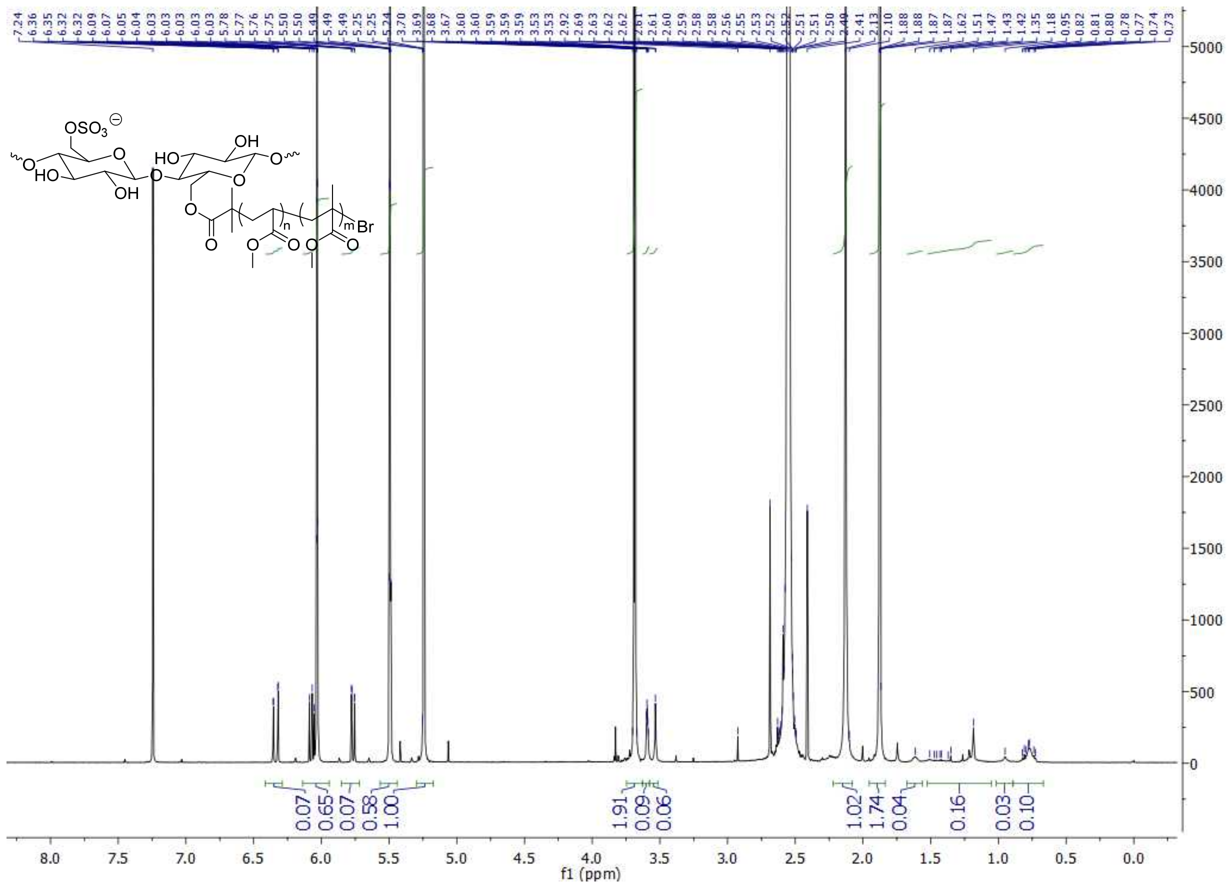

Figure S36. ^{1}H NMR spectrum of an aliquot taken from the reaction mixture to produce **CNC-*g*-PMA$_8$-*b*-PMMA$_2$** just before the reaction was quenched. The spectrum was recorded in $CDCl_3$.

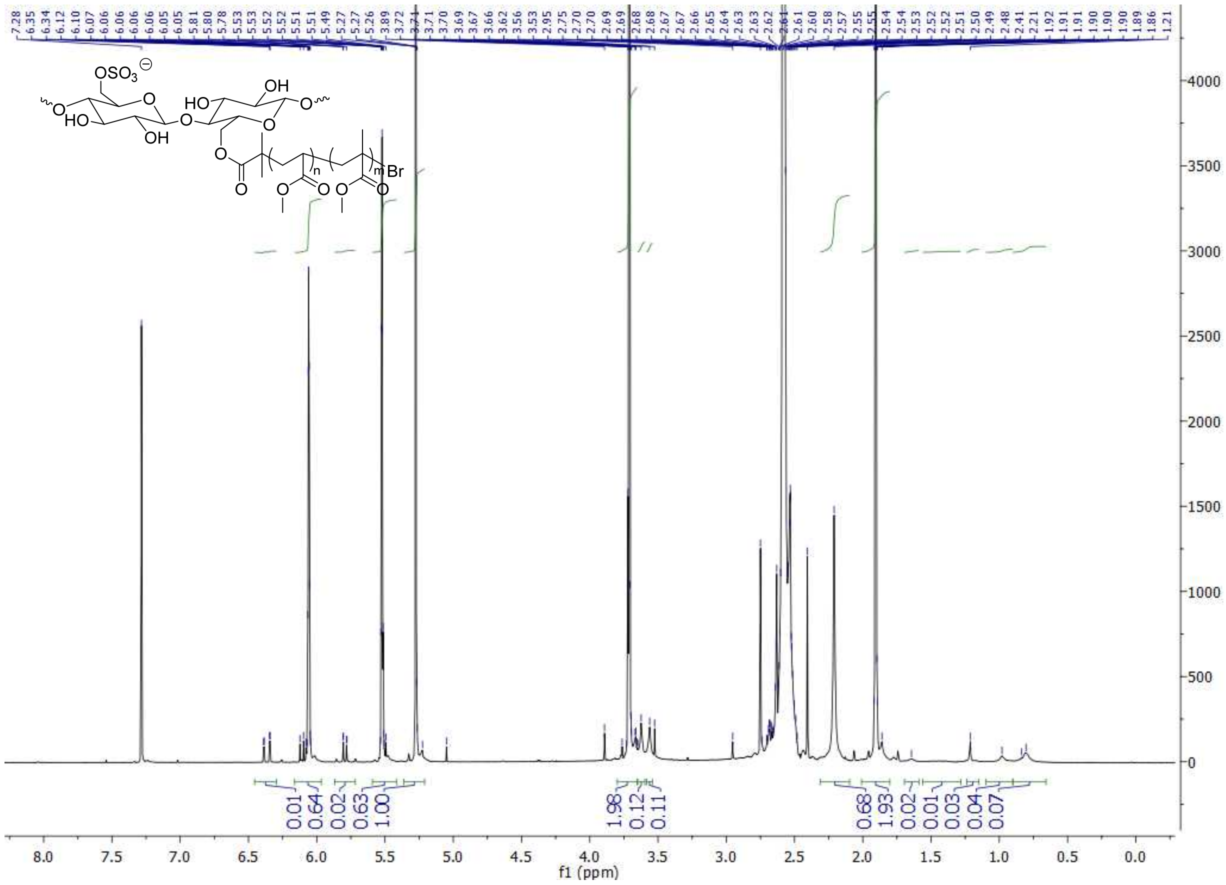

Figure S37. ^{1}H NMR spectrum of an aliquot taken from the reaction mixture to produce **CNC-*g*-PMA$_8$-*b*-PMMA$_7$** just before the reaction was quenched. The spectrum was recorded in $CDCl_3$.

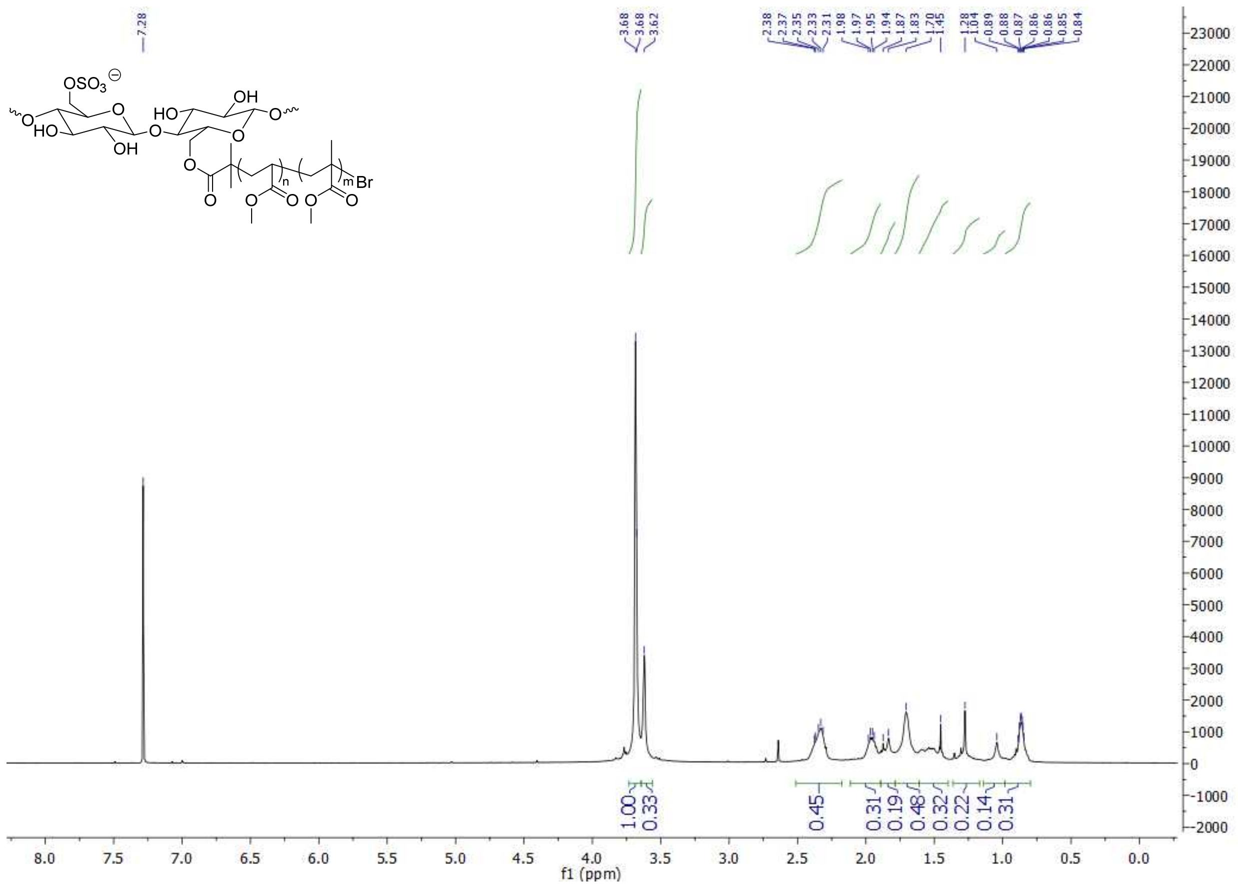

Figure S38. ^{1}H NMR spectrum of **CNC-*g*-PMA$_8$-*b*-PMMA$_2$** (isolated after a reaction time of 120 + 60 min and purified by dialysis against THF) that was redispersed in $CDCl_3$.

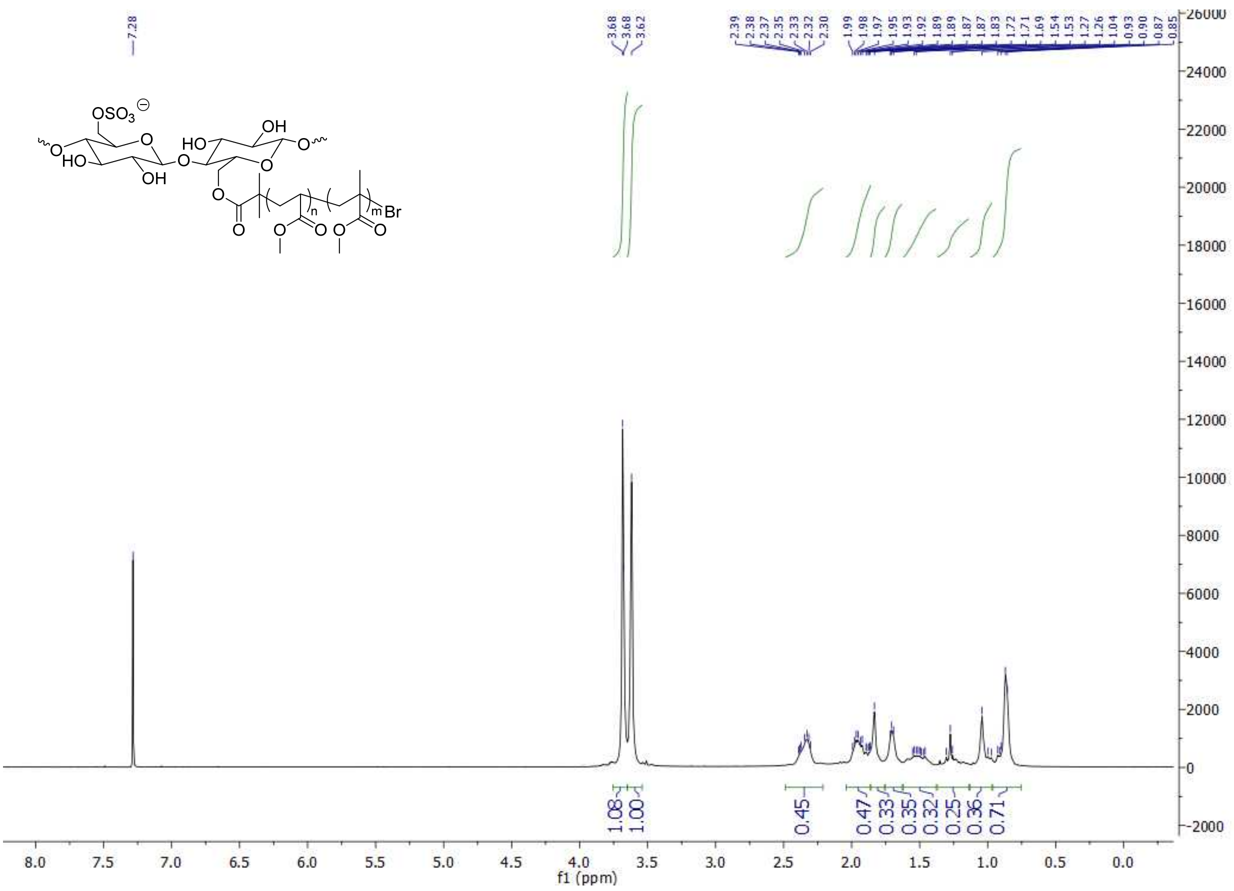

Figure S39. ^{1}H NMR spectrum of **CNC-*g*-PMA$_8$-*b*-PMMA$_7$** (isolated after a reaction time of 120 + 120 min and purified by dialysis against THF) that was redispersed in $CDCl_3$.

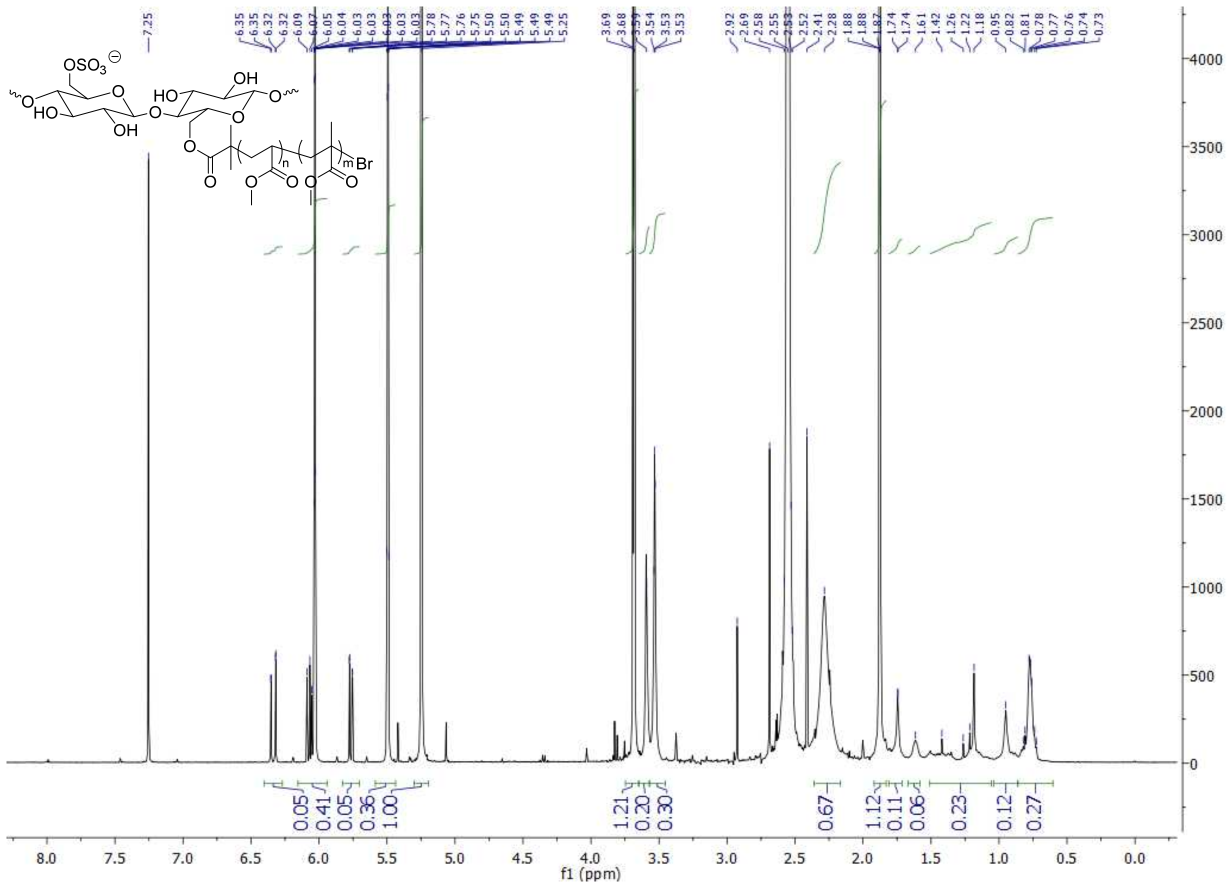

Figure S40. ^{1}H NMR spectrum of an aliquot taken from the reaction mixture to produce **CNC-*g*-PMA$_5$-*b*-PMMA$_7$** just before the reaction was quenched. The spectrum was recorded in $CDCl_3$.

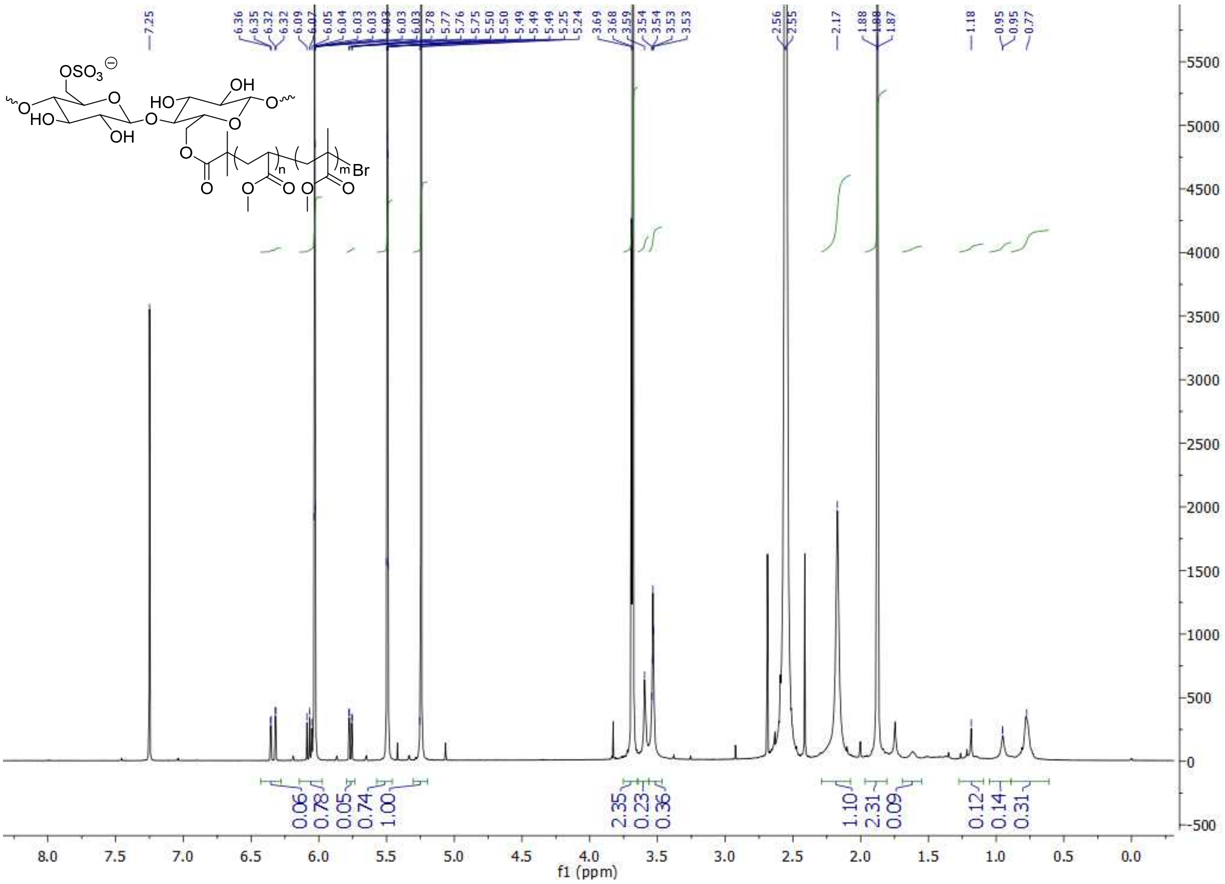

Figure S41. ^{1}H NMR spectrum of an aliquot taken from the reaction mixture to produce **CNC-*g*-PMA$_5$-*b*-PMMA$_{12}$** just before the reaction was quenched. The spectrum was recorded in $CDCl_3$.

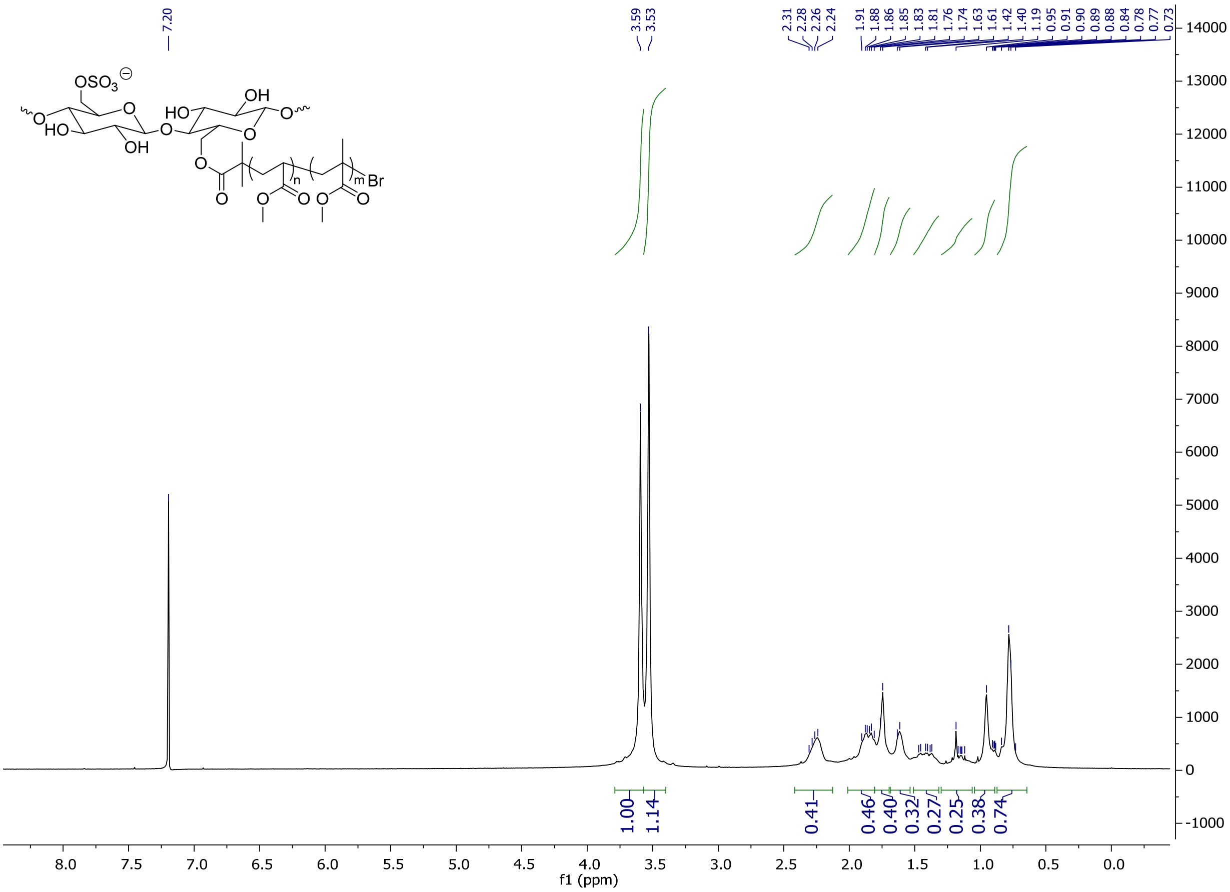

Figure S42. ^{1}H NMR spectrum of **CNC-*g*-PMA$_5$-*b*-PMMA$_7$** (isolated after a reaction time of 60 + 60 min and purified by dialysis against THF) that was redispersed in $CDCl_3$.

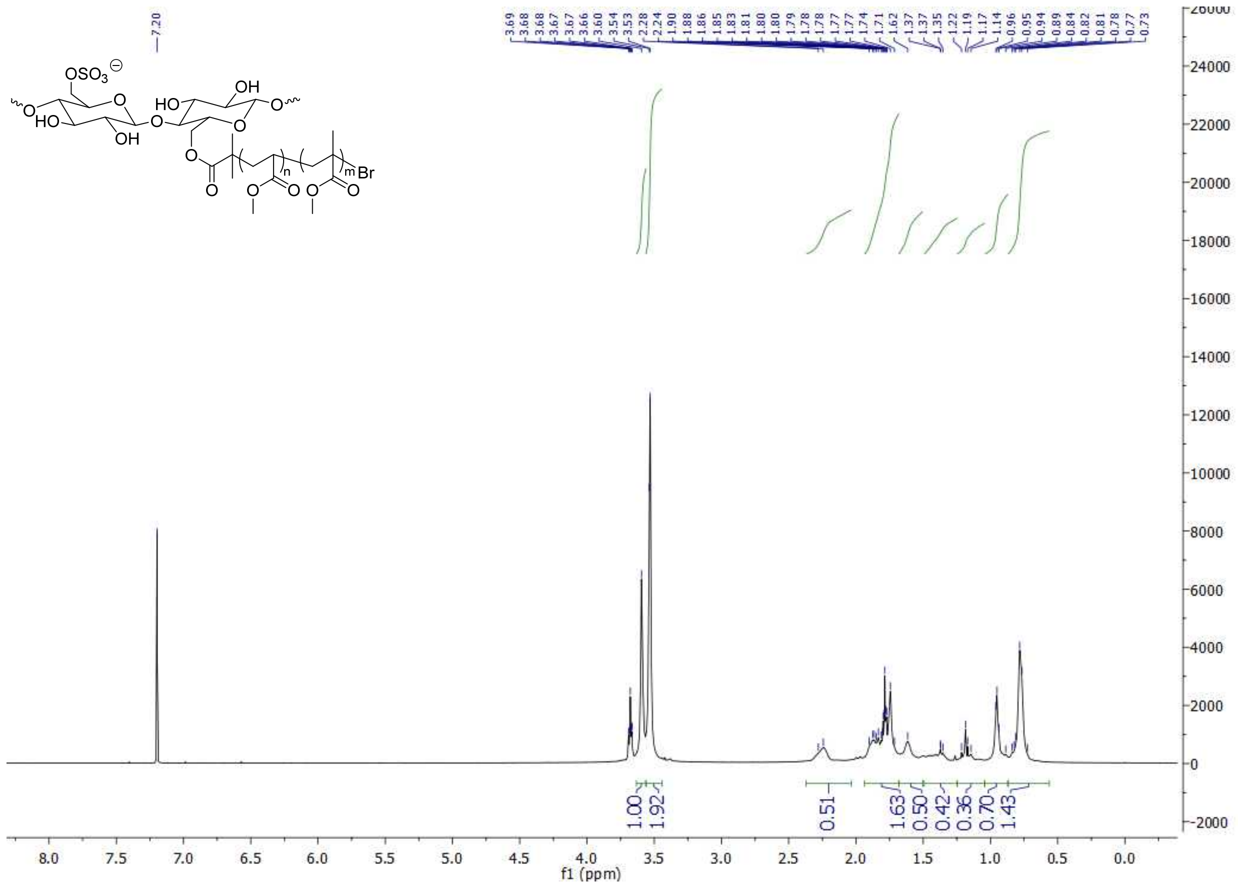

Figure S43. ^{1}H NMR spectrum of **CNC-*g*-PMA$_5$-*b*-PMMA$_{12}$** (isolated after a reaction time of 60 + 120 min and purified by dialysis against THF) that was redispersed in $CDCl_3$. Signals at 3.73-3.76 ppm and 1.84-1.87 ppm are related to residual THF.

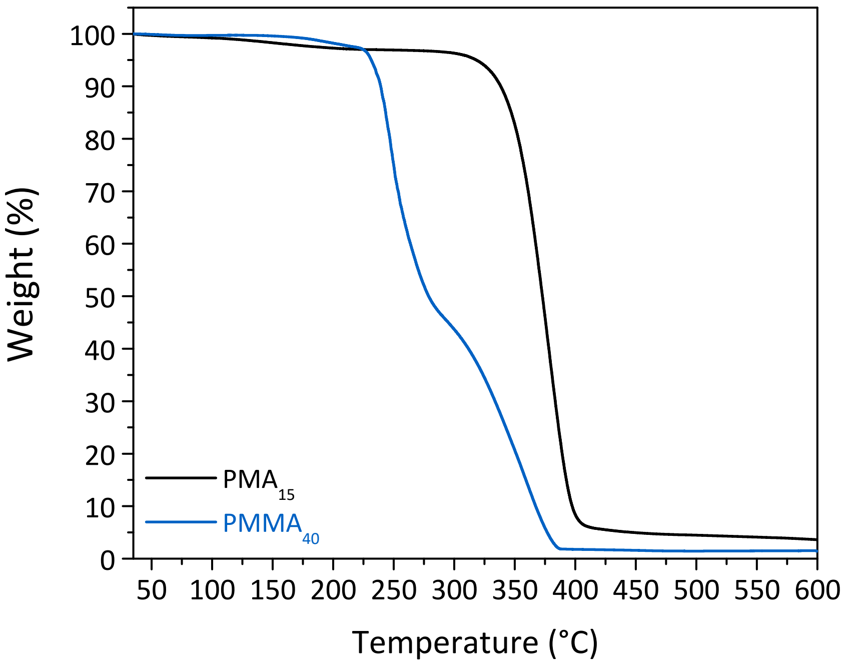

Figure S44. Thermogravimetric analysis (TGA) traces of $\mathbf{PMA_{15}}$ and $\mathbf{PMMA_{40}}$ recorded at a heating rate of 10 °C min^{-1}.

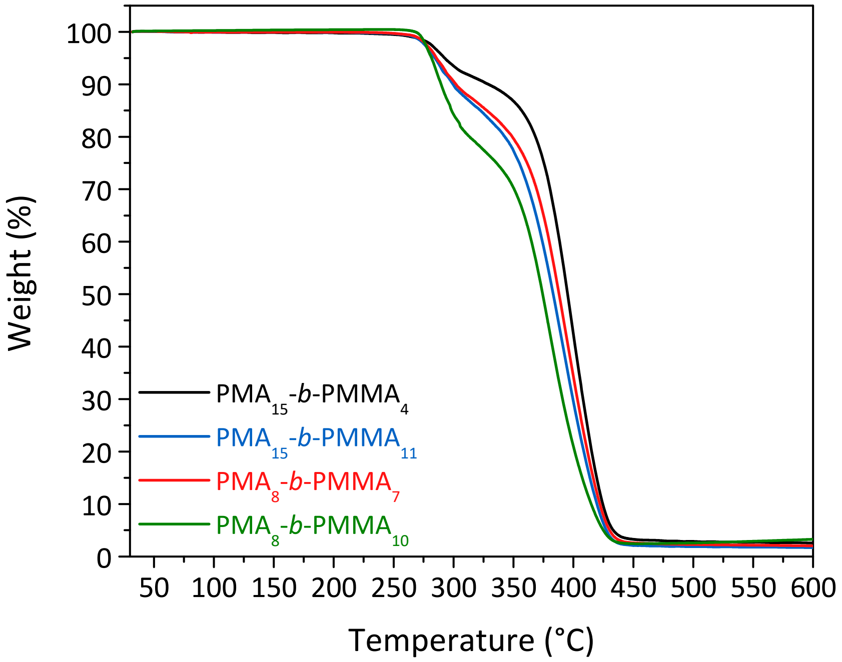

Figure S45. TGA traces of PMA-*b*-PMMA block copolymers recorded at a heating rate of 10 °C min^{-1}.

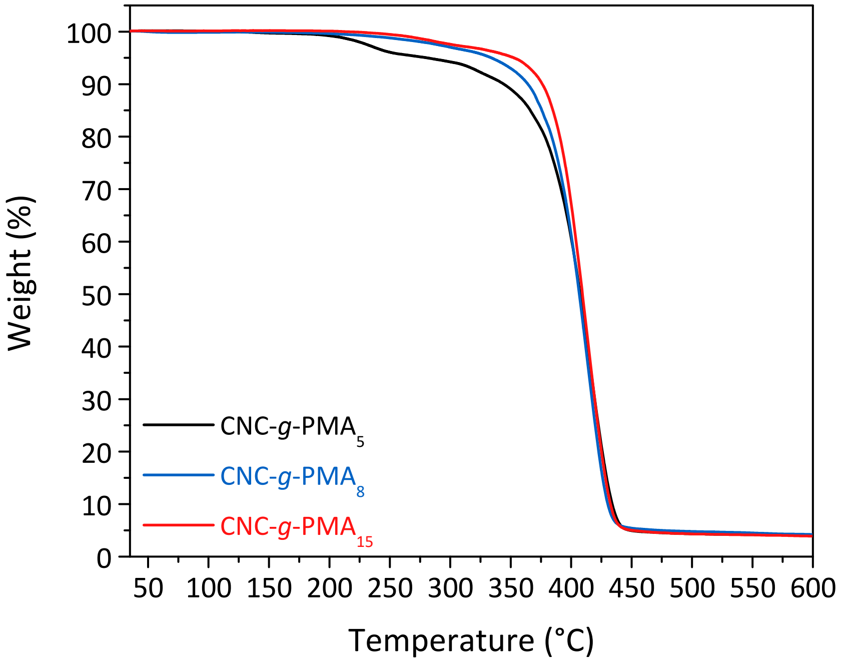

Figure S46. TGA traces of CNC-*g*-PMA OCNs recorded at a heating rate of 10 °C min^{-1}.

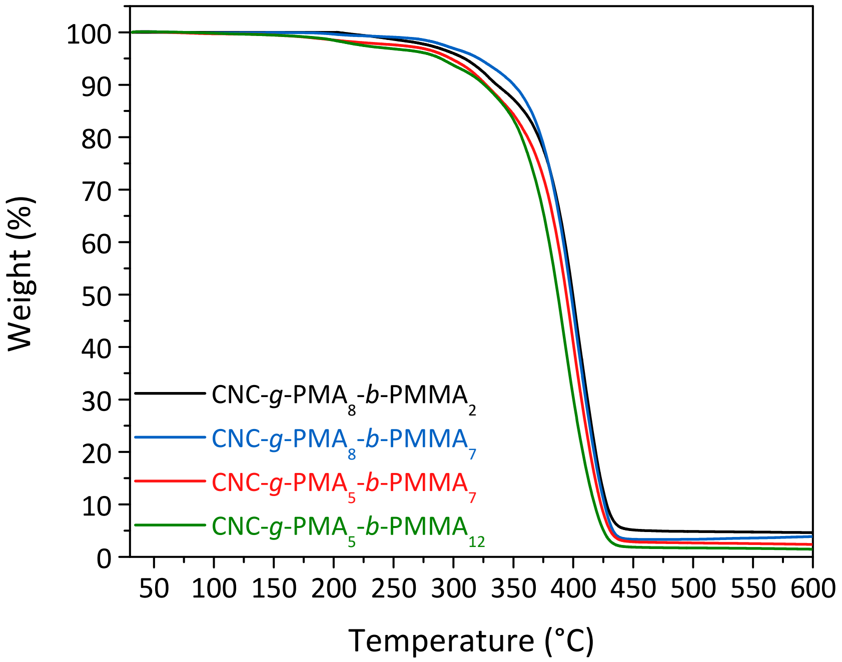

Figure S47. TGA traces of CNC-*g*-PMA-*b*-PMMA OCNs recorded at a heating rate of 10 °C min^{-1}.

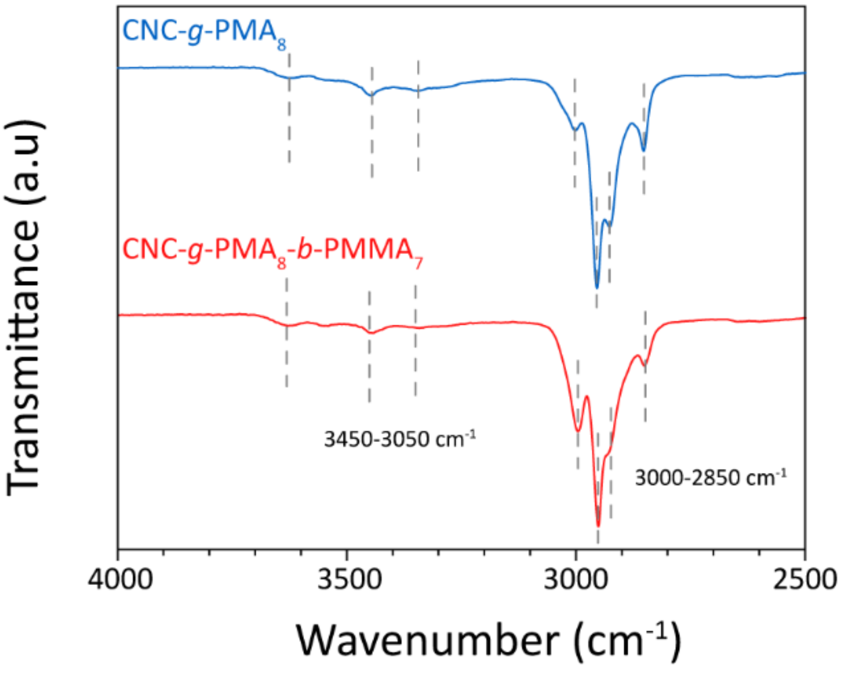

Figure S48. Magnification of portions of the FT-IR spectra of CNC-*g*-PMA_8 and CNC-*g*-PMA_8-*b*-$PMMA_7$.

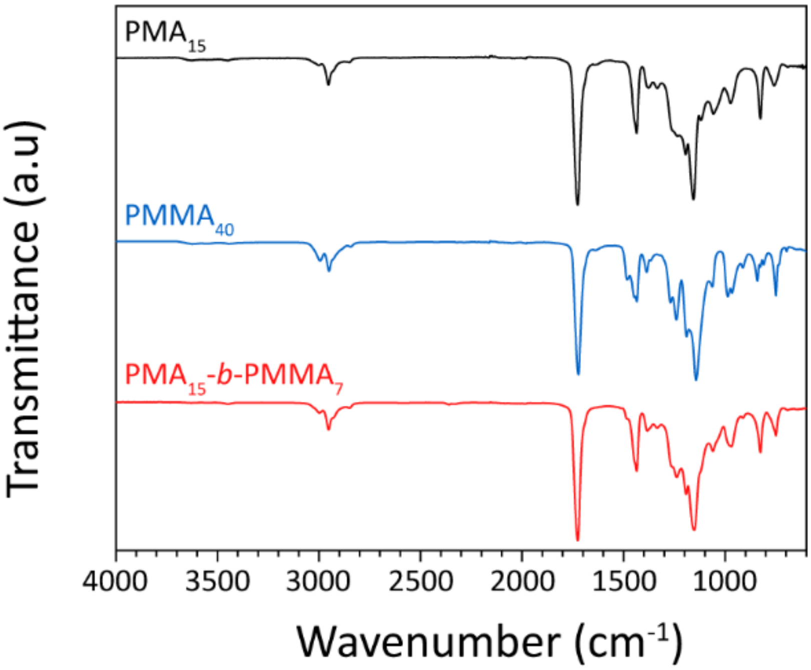

Figure S49. FT-IR spectra of PMA_{15}, $PMMA_{40}$, and PMA_{15}-*b*-$PMMA_7$.

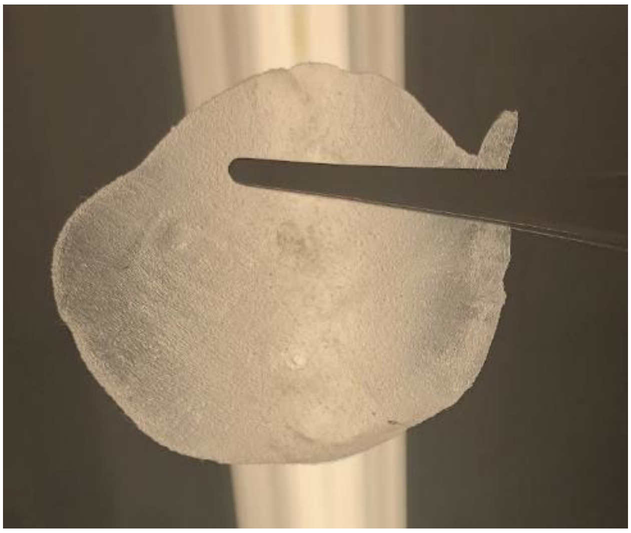

Figure S50. Picture of a film of CNC-*g*-PMA_{15} after hot pressing.

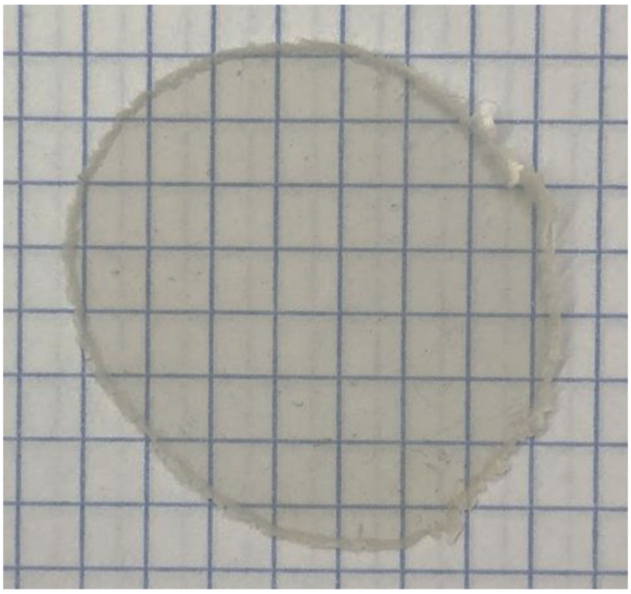

Figure S51. Picture of a film of CNC-*g*-PMA8-*b*-PMMA7 after hot pressing.

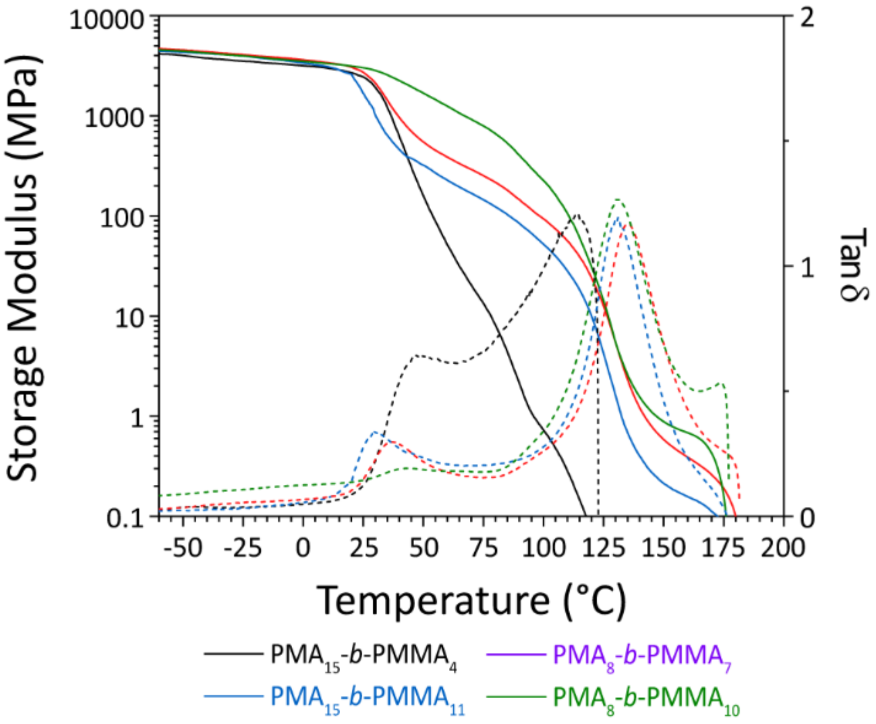

Figure S52. DMA traces of PMA-*b*-PMMA block copolymers.

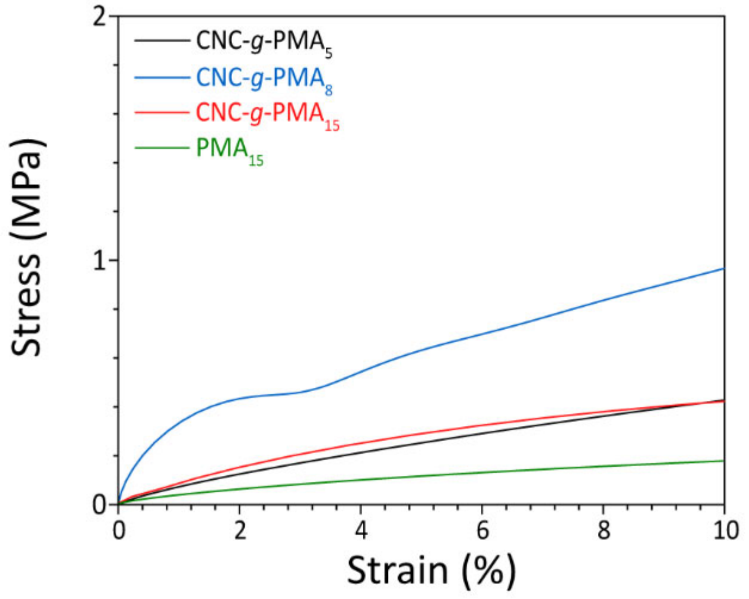

Figure S53. Tensile tests of CNC-*g*-PMA. The graph shows a magnification of the low- strain regime. Tests were conducted at 50 % min^{-1} at room temperature.

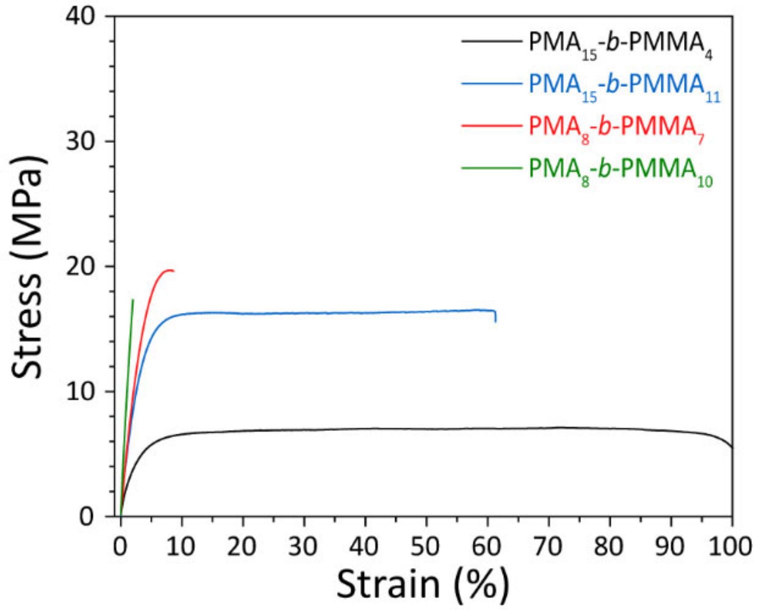

Figure S54. Tensile tests of the PMA-*b*-PMMA reference free-polymers conducted at 50 % min^{-1} and at room temperature.

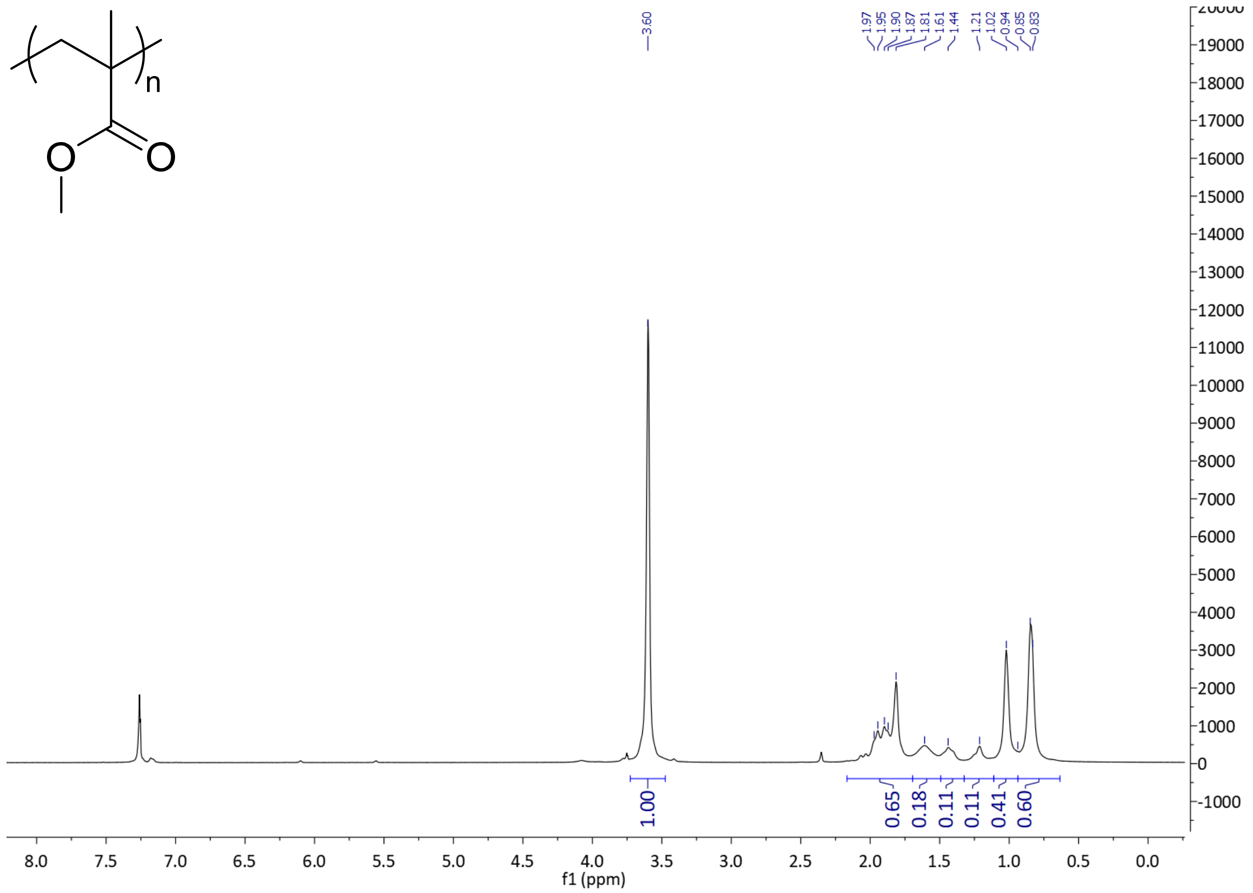

Figure S55. ^{1}H NMR spectrum of commercial PMMA (**PMMA$_{40}$**) in $CDCl_3$.

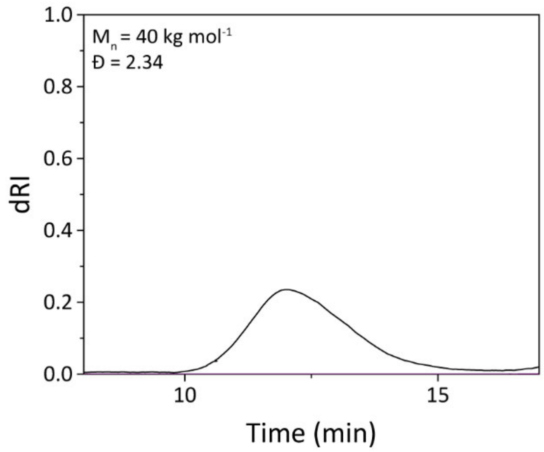

Figure S56. SEC trace of commercial PMMA (**PMMA$_{40}$**) in $CDCl_3$. THF was used as eluent.

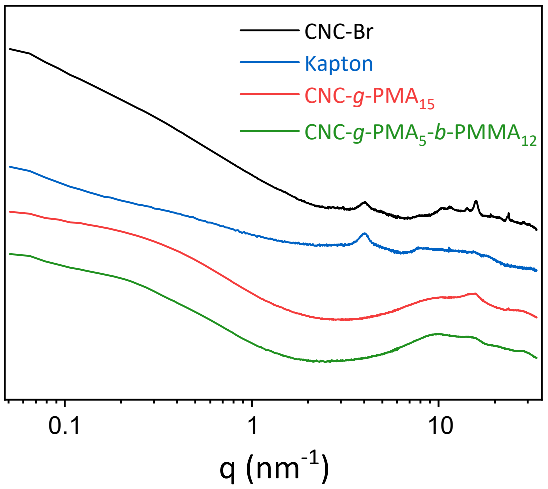

Figure S57. Characterization of the CNC-Br, CNC-*g*-PMA, and CNC-*g*-PMA-*b*-PMMA by X-ray scattering. Small- and wide-angle scattering profile of CNC-BR was recorded in a powder state with Kapton as the reference material. Measurements of CNC-*g*-PMA and CNC-*g*-PMA-*b*-PMMA were conducted after the nanocomposites were pressed into 250 µm thick films.

2 Supplementary Tables S1-3

Table S1. Reaction conditions and composition of PMA, PMMA, and PMA-*b*-PMMA reference polymers.

Sample Name	Reaction times PMA/PMMA block (h)	Equivalents MA/MMA in feed (-)	PMA/PMMA fraction in polymer[a] (mol / mol)	PMA/PMMA M_n NMR (kg mol^{-1})[b]	PMA/PMMA M_n SEC[c] (kg mol^{-1})
PMA_{15}	1 / -	200 / -	100 / 0	15.9	15.7
$PMMA_{40}$	-[d]	-[d]	0 / 100[d]	-	40.2
PMA_{15}-*b*-$PMMA_{4}$	1 / 1	200 / 400	75 / 25	15 / 4	15.5 / 41.8
PMA_{15}-*b*-$PMMA_{11}$	1 / 2	200 / 400	61 / 39	15 /11	16.1 / 51.2
PMA_{8}-*b*-$PMMA_{7}$	1 / 1	100 / 400	53 / 47	8 / 7	44.6
PMA_{8}-*b*-$PMMA_{10}$	1 / 2	(100 / 400)	43 / 57	8 / 10	29.7

[a]Determined from the ratio of the integrals of the methyl group signals of PMA (3.66 ppm) and PMMA (3.60 ppm) in the ^{1}H NMR spectrum of a sample taken from the reaction mixture just before quenching the reaction. [b]Calculated using Eqs. S1 and S2 from the monomer conversion, which was determined from the integrals of signals associated with monomer (6.36, 6.07, 5.75 ppm) and polymer (3.66 ppm) in the ^{1}H NMR spectrum of a sample taken from the reaction mixture just before quenching the reaction. [c]Measured by SEC of the isolated polymer. [d]Commercial sample.

Table S2. Mechanical and thermomechanical properties of PMA, PMMA, and PMA-*b*-PMMA reference polymers.

Sample	E_y[a] (MPa)	σ_{UTS}[a] (MPa)	ε_B[a] (%)	Tensile Toughness[a] (MJ m^{-3})	E' at -60 °C[b] (MPa)	$T_{g,1}$[b] (° C)	$T_{g,2}$[b] (° C)
PMA_{15}	9 ±0.6	0.15 ±0.02	265 ±29	0.17 ±0.0	2590 ±380	26.2 ±0.5	-
$PMMA_{40}$	1770 ±280	47 ±7	4 ±1	1.1 ±0.4	4700 ±120	-	120 ±1
PMA_{15}-*b*-$PMMA_{4}$	360 ±20	7 ±1	120 ±30	0.68 ±0.0	4150 ±430	46.5 ±3	n.d.[c]
PMA_{15}-*b*-$PMMA_{11}$	550 ±70	16 ±0.3	58 ±5	0.95 ±0.1	4300 ±370	31.4 ±3	131 ±2
PMA_{8}-*b*-$PMMA_{7}$	650 ±70	18 ±3	9 ±1	0.96 ±0.2	4450 ±290	37.6 ±1	134 ±3
PMA_{8}-*b*-$PMMA_{10}$	1190 ±150	17 ±0.3	2 ±0.2	0.02 ±0.0	4500 ±370	44.5 ±5	131 ±2

[a]Determined by tensile tests. [b]Determined by dynamic mechanical analysis; T_g values represent the maxima of the tanδ curves. Not detected, as the sample fails.

Table S3. Monomer conversion determined by ^{1}H NMR analysis through an internal standard (IS) versus comparing the monomer to polymer integrals.

Sample	Conversion based on IS[a] (%)	M_n (IS) (kg mol^{-1})[a]	Conversion based on Polymer Formed[b] (%)	M_n (Based on Polymer Formed) (kg mol^{-1})[b]
PMA_{15} (1h)	84	14.5	87	15.0
CNC-g-PMA_{15} (1h)	66	11.5	68	11.8
CNC-g-PMA_{15} (2h)	85	14.7	82	14.1

[a]Determined using equation S4 and S5. [b]Determined using equation S3 and S4.

3 Supplementary Equations S1-5

$$\text{SSA} = \frac{m_{CNC}}{\rho_{cellulose}} \frac{2\pi H + 4(W - H)}{\pi W H} \qquad \text{Eq. S1}$$

Equation S1. Equation for calculating the specific surface area (SSA) of CNCs. Further details can be found in the experimental section.

$$\sigma_i = \frac{\frac{w_i}{MW_i} \times N_A}{(1 - w_i) \times SSA \times 10^{18}} \qquad \text{Eq. S2}$$

Equation S2. Equation for calculating the initiator grafting density. Further details are can be found in the experimental section.

$$\% \, Conversion = \left(\frac{\frac{\int Polymer\ @\ t_x}{\#H,polymer}}{\frac{\int Polymer\ @\ t_x}{\#H,polymer} + \frac{\int Monomer\ @\ t_x}{\#H,monomer}} \right) \times 100 \qquad \text{Eq. S3}$$

Equation S3. The conversion of monomer into polymer was calculated on the basis of the integration of ^{1}H NMR signals associated with monomer and polymer. #H,polymer (3) and #H,monomer (3) indicate the number of protons associated with the signals observed at chemical shifts of 6.36, 6.07, and 5.75 ppm for the monomer and 3.66 ppm for the polymer. ∫ Polymer @ t_x = integral of the signal at 3.66 ppm, ∫ Monomer @ $t_{x=}$ integral of signals at 6.36, 6.07, and 5.75 ppm; this value was set to 1.

$$M_{n\,\text{NMR}} = M_{monomer} \times (Monomer\ Eq.) \times \left(\frac{\%\,Conversion}{100}\right) + M_{Initiator} \qquad \text{Eq. S4}$$

Equation S4. The $M_{n\,\text{NMR}}$ was calculated from the Conversion determined with Eq. S1 assuming living conditions. M_{monomer} and $M_{\text{initiator}}$ are the molecular weights of the monomer (MA, 85.01 g mol^{-1}) and initiator (195.05 g mol^{-1}). Monomer Eq. represents the equivalence of the monomer to the initiator.

$$\% \, Conversion = [1 - \left(\frac{\frac{\int Monomer\ @\ t_x}{\int IS\ @\ t_x}}{\frac{\int Monomer\ @\ t_0}{\int IS\ @\ t_0}} \right)] x\ 100 \qquad \text{Eq. S5}$$

Equation S5. The conversion of monomer into polymer was calculated on the basis of the integration of ^{1}H NMR signals associated with monomer and dichloromethane (DCM, 2.52 ppm) that was used as an internal standard. Aliquots of the reaction mixture were filtered through alumina before 5 µL of DCM were added for ^{1}H NMR analysis. ∫ Monomer @ t_x integral of signals at 6.37, 6.07, and 5.78 ppm. ∫ IS @ t_x and ∫ IS @ t_0 are integrals at 5.25 ppm that were set to 1.
